# The Persistence of Hydrogen Bonds in Pyrimidinones: From Solution to Crystal

**DOI:** 10.1021/acsorginorgau.4c00057

**Published:** 2024-09-05

**Authors:** Fellipe F. S. Farias, Mateus Mittersteiner, Amanda M. Kieling, Priscila S. V. Lima, Gustavo H. Weimer, Helio G. Bonacorso, Nilo Zanatta, Marcos A. P. Martins

**Affiliations:** Núcleo de Química de Heterociclos (NUQUIMHE), Department of Chemistry, Federal University of Santa Maria (UFSM), 97105-900 Santa Maria, Rio Grande do Sul, Brazil

**Keywords:** hydrogen bonds, pyrimidinones, crystallization mechanisms, intermolecular interactions, supramolecular cluster

## Abstract

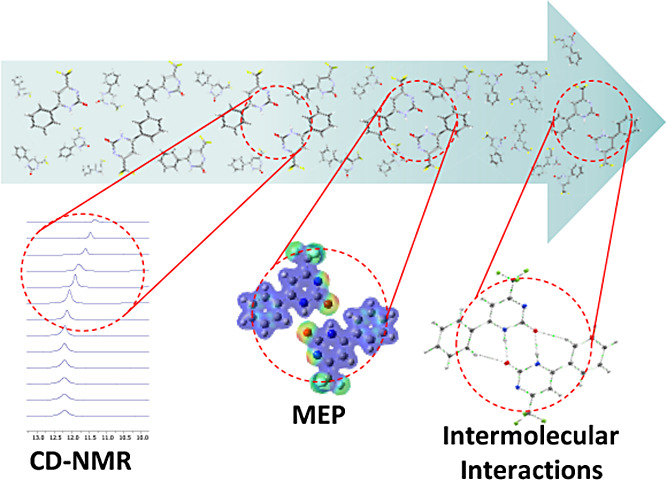

Pyrimidinone scaffolds are present in a wide array of molecules with synthetic and pharmacological utility. The inherent properties of these compounds may be attributed to intermolecular interactions analogous to the interactions that molecules tend to establish with active sites. Pyrimidinones and their fused derivatives have garnered significant interest due to their structural features, which resemble nitrogenous bases, the foundational building blocks of DNA and RNA. Similarly, pyrimidinones are predisposed to forming N–H···O hydrogen bonds akin to nitrogenous bases. Given this context, this study explored the supramolecular features and the predisposition to form hydrogen bonds in a series of 18 substituted 4-(trihalomethyl)-2(1*H*)-pyrimidinones. The formation of hydrogen bonds was observed in solution via nuclear magnetic resonance (NMR) spectroscopy experiments, and subsequently confirmed in the crystalline solid state. Hence, the 18 compounds were crystallized through crystallization assays by slow solvent evaporation, followed by single-crystal X-ray diffraction (SC-XRD). The supramolecular cluster demarcation was employed to evaluate all intermolecular interactions, and all crystalline structures exhibited robust hydrogen bonds, with an average energy of approximately −21.64 kcal mol^–1^ (∼19% of the total stabilization energy of the supramolecular clusters), irrespective of the substituents at positions 4, 5, or 6 of the pyrimidinone core. To elucidate the nature of these hydrogen bonds, an analysis based on the quantum theory of atoms in molecules (QTAIM) revealed that the predominant intermolecular interactions are N–H···O (average of −16.55 kcal mol^–1^) and C–H···O (average of −6.48 kcal mol^–1^). Through proposing crystallization mechanisms based on molecular stabilization energy data and contact areas between molecules and employing the supramolecular cluster and retrocrystallization concepts, it was determined that altering the halogen (F/Cl) at position 4 of the pyrimidinone nucleus modifies the crystallization mechanism pathway. Notably, the hydrogen bonds present in the initial proposed steps were confirmed by ^1^H NMR experiments using concentration-dependent techniques.

## Introduction

The pyrimidinone scaffold has been widely recognized as a versatile starting material for constructing target molecules that exhibit promising pharmacological and/or biological properties.^1−4^ This widespread application is strongly correlated with the structural features of pyrimidinones, which are similar in size, volume, and chemical structure to the nitrogenous bases present in DNA and RNA.^2,3^ Furthermore, analogous to the nitrogenous bases in DNA and RNA, pyrimidinones possess the ability to form strong hydrogen bonds, a key structural feature.^3,5,6^

The literature has shown the medicinal activity of pyrimidinones across various therapeutic segments.^2,3^ Their anticancer,^7−10^ anticonvulsant,^11,12^ antidiabetic,^13,14^ anti-inflammatory,^3,15^ antimicrobial,^16,17^ and antiviral^4,18−20^ properties are well-established and continue to advance. Likewise, the pyrimidinone core is present in drugs currently on the market, such as lamivudine, a retroviral agent for HIV treatment,^21,22^ and tegafur/uracil, a chemotherapy drug used in cancer treatment.^23,24^ The versatile properties of pyrimidinones are primarily attributed to two characteristics: (i) at the molecular level, the deactivation of the cyclic ring induced by the presence of nitrogen atoms confers high reactivity to the ring,^8,25^ and (ii) at the supramolecular level, strong hydrogen bonds are promoted between the pyrimidinone nucleus and the target sites.^26−28^ Additionally, due to its similarities with the nitrogenous bases of nucleic acids, the pyrimidinone scaffold ranks among the most extensively explored and studied organic nuclei in the literature to date.^29−33^

The bioactivity of pyrimidinones, akin to other bioactive molecules, may be associated with intermolecular interactions. The interactions that molecules form with target active sites often mirror those found in the crystal or in solution. These interactions tend to dissociate in self-association, for instance, to reestablish themselves with the target active site.^34−38^ Therefore, comprehending the nature, quality, and quantity of interactions in both the crystalline solid state and in solution is an essential step for future applications.

Understanding the crystalline organization and interpreting these supramolecular systems requires acknowledging that a crystal is a complex system. The parts (molecules, compounds, and ions) form the whole (the crystal lattice). As the adage suggests, “the whole is greater than the sum of its parts.″ When the parts possess inherent properties, these properties can be modulated when the parts are associated, and newly unique properties, known as emergent properties, may arise.^39,40^ This phenomenon influences the potential applications of crystals. Thus, understanding the crystallization process is crucial for effectively employing crystals and designing new molecules with predictable and desired properties.^40−43^

Recently, our research group synthesized a series of 4-(trihalomethyl)-2(1*H*)-pyrimidinones with various substituents at positions 5 and 6 of the pyrimidinone ring to evaluate their reactivity as building blocks for functionalized heterocycles.^44−46^ Both electron-donating and electron-withdrawing groups were introduced at different positions of the pyrimidinone nucleus, and the halogen at position 4 was also modulated. During these investigations, we identified a gap in the literature regarding these compounds’ characteristics and supramolecular crystalline organization. Consequently, we aimed to crystallize 4-(trihalomethyl)-2(1*H*)-pyrimidinones as models to assess their peculiarities in the crystalline solid state. These compounds hold significant relevance as models for synthetic chemists and researchers investigating their therapeutic properties.^1−4^

**Figure 1:**
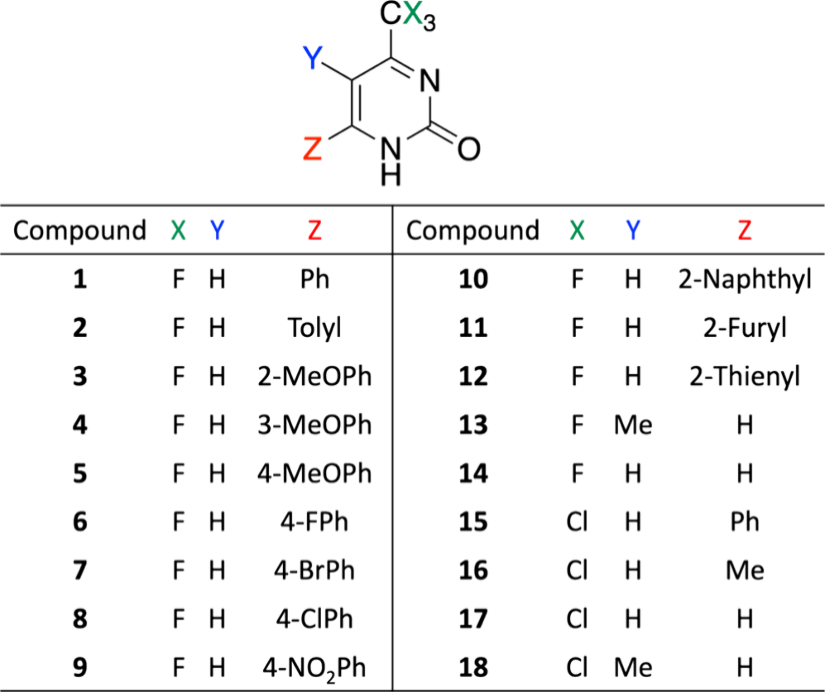
Pyrimidinones used in this study. Columns *X*, *Y*, and *Z* correspond to positions 4, 5, and 6 of the pyrimidinone nucleus, respectively.

To understand the formation process of crystals and the interactions within their lattices, this study aimed to investigate the intermolecular interactions present in 4-(trihalomethyl)-2(1*H*)-pyrimidinones both in solution and in the solid state and, more specifically, examine how different substituents at positions 4, 5, and 6 of the pyrimidinone ring influence these interactions. For this, we employed the supramolecular cluster as a demarcation tool along with the retrocrystallization phenomena, proposals for crystallization mechanisms were elucidated. Allied with these crystallization proposals, experimental methods, including concentration dependent ^1^H nuclear magnetic resonance (CD-NMR) spectroscopy, were utilized to simulate saturated solutions analogous to the initial stages of the crystallization process and shed more light on the intermolecular interactions present in solution. Therefore, we selected 18 pyrimidinone molecules along with their respective substituents at positions 4, 5, and 6 of the pyrimidinone ring, as illustrated in Figure 1.

## Experimental Section

### General Synthetic Procedure for 4-(Trihalomethyl)-2(1*H*)-pyrimidinones

**Scheme 1:**
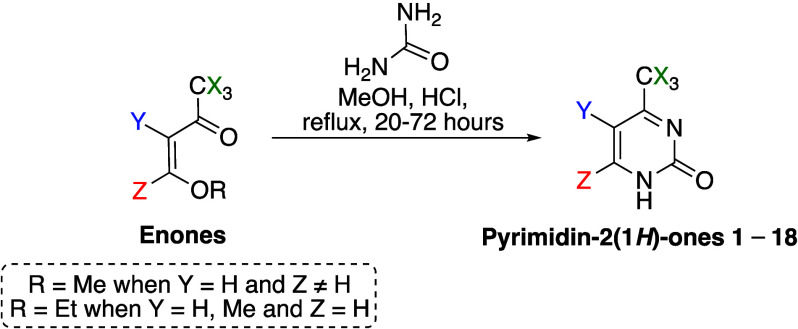
General Reaction for the Formation of the Pyrimidinones Used in This Study

A magnetically stirred solution of substituted trihalo-3-alken-2-ones (enones) (5 mmol) and urea (10 mmol, 0.6 g) in 10 mL of methanol at 20–25 °C received 1 mL of concentrated HCl. This mixture was then refluxed at 65 °C for 20–72 h. Distilled water (15 mL) was added to the cooled reaction mixture at 25 °C, and the products were allowed to crystallize by further cooling the solutions to 5–8 °C for 12 h. The solids were filtered, washed with cold water, and dried overnight in a desiccator at room temperature. Then, the products were recrystallized from dichloromethane/ethanol 1:1 mixture or from ethanol 96%. The general procedure is summarized in Scheme 1, further details can be found elsewhere.^47−54^

### Single-Crystal X-ray Diffraction

Single crystals of compounds **1**–**18** were obtained through slow evaporation, utilizing solvents listed in Supporting Information (SI), Table S1. Several attempts were made using a range of solvents for all compounds to accomplish different crystalline phases, however, single crystals were not obtained or were unsuitable for X-ray diffraction experiments in some solvents. Details on crystals obtained in different solvents are described in Table S1. It is worth noting that solvates forms were not obtained.

Diffraction measurements for compounds **2**–**18** were conducted on a Bruker D8 Venture equipped with a Photon 100 CMOS detector using graphite monochromatized Mo Kα radiation (λ = 0.71073 Å). The diffraction measurement of compound **1** was conducted on a Bruker D8 Quest diffractometer with Cu Kα radiation (λ = 1.54080 Å) and a KAPPA four-circle goniometer matched with a Photon 100 CMOS area detector. Both setups are located in the chemistry department of the Federal University of Santa Maria (UFSM). Measurements were made up to a resolution of (sin θ/λ) max = 0.60 Å^–1^ at 298 K or under N_2_ gas set at 100 K. Absorption corrections were applied using the multiscan method (SADABS).^55^ Integration of the frames was performed with the Bruker SAINT software package. Anisotropic displacement parameters were applied for non-hydrogen atoms.^56^ The structures were refined using the SHELXL software integrated with the Olex2 program, based on the full matrix least-squares method.^57^ Parameters for X-ray data collection and structure refinement are provided in Table S2 on SI.

Graphical representations were generated with the Mercury 2024 software (version 1.0), which illustrates spatial arrangement of the atoms and their respective directions of thermal vibration through the construction of ellipsoids. The ellipsoids reflect the probability of the atoms being in a given average region. For all compounds, ellipsoids with 50% thermal vibration were used.^58^ Crystallographic Information Files (.cif) for the studied compounds have been deposited at the Cambridge Crystallographic Data Center (CCDC) under the reference numbers: 2359091–2359097, 2359102, 2359103, 2359105, 2359107, 2359108, 2359112–2359114, 2359116–2359118.

### Contact Area, Stabilization Energy, Normalization Data, Crystallization Mechanism, Quantum Mechanical Calculations and Molecular Electrostatic Potential Surfaces

The supramolecular cluster^40^ is defined by considering all molecules that have contact area with the reference molecule (M1; typically having the symmetry code x,y,z). For this purpose, the contact area from the Voronoi–Dirichlet polyhedra (VDP) was determined using the ToposPro software.^59^ Once the supramolecular cluster has been constructed, the stabilization energy (*G*) and contact area (*C*) data are obtained for each dimer formed between the M1 molecule and all MN molecules (M1···M*N*). The VDP is used to ascertain the contact area value for each dimer (*C*_M1···M*N*_) considering a set of atomic VDP faces corresponding to the adjacent contact areas between the atoms of the two molecules. Here, we consider the contact area in Å^2^ provided by the software. The sum of the set of M*N* molecules is regarded as the molecular coordination number (*N*). The intermolecular stabilization energy of each dimer (*G*_M1···MN_) is determined according to eq 1.
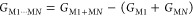
1Where *G*_M1+M*N*_ is the total stabilization energy of the considered dimer, *G*_M1_ is the stabilization energy of the M1 molecule, and *G*_M*N*_ is the stabilization energy of the considered monomer. The normalization of the raw contact area (NC_M1···M*N*_) and stabilization energy (NG_M1···M*N*_) data is determined as indicated in eqs 2 and 3, respectively.
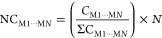
2
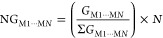
3

The quantitative stage parameters, *N*_CG_% and NG/NC,^60^ are determined using eqs 4 and 5, respectively.
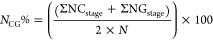
4
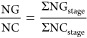
5Where *N*_CG_% corresponds to the contribution of the considered stage in relation to the total crystallization process in terms of stabilization energy and contact area; NG/NC coincides with the parameter that drives the considered stage. When NG/NC > 1.0, stabilization energy governs the considered stage, whereas when NG/NC < 1.0, contact area dominates.^40,60^

The intermolecular interactions energies of the compounds were determined through single-point calculations using geometries obtained from single-crystal X-ray diffraction. Density functional theory (DFT) calculations were conducted to determine the stabilization energy using the Gaussian 09 software package, and these calculations were performed in single-point mode at ωB97XD/cc-pVDZ theory level utilizing geometries derived from the SC-XRD without any molecular geometry optimization. The Boys and Bernardi counterpoise correction was utilized to minimize the basis set superposition error (BSSE).^61^ The energy derived (*G*) represents the electronic energy provided by Gaussian software. Wave functions for the QTAIM analysis were generated at the ωB97XD/cc-pVDZ level of theory; QTAIM analyses were conducted with the AIMAll program package.^62^ The presence of intermolecular interactions was indicated by a bond critical point (BCP) within the bond path, which connects two interacting atoms.^63^ Stabilization energy for each atom···atom interaction was obtained utilizing *G*_AI_ analysis^64−66^ (eq 6).
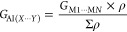
6

These results correlate the QTAIM data (ρ_BCP_) and the stabilization energy of the considered dimer from the supramolecular cluster.

The molecular electrostatic potential (MEP) surfaces were constructed on an electron density 0.01 au isosurface with GaussView software.

### Concentration-Dependent ^1^H Nuclear Magnetic Resonance (CD-NMR) Experiments

The CD NMR experiments were conducted on a Bruker Avance III (^1^H at 600.130 MHz) spectrometer in CDCl_3_ at 298 K using tetramethylsilane (TMS) as an internal standard. Compounds **1**, **3**, **11**, **13**, **15** and **16** were analyzed at varying concentrations, ranging from 0.006 to 0.200 mol L^–1^, according on their solubility.

### Solid-State Nuclear Magnetic Resonance

Solid-state nuclear magnetic resonance (SS-NMR) data were recorded using a Bruker Avance III spectrometer (600 MHz) with a Larmor frequency of 150.903 MHz for ^13^C NMR. The ^13^C CP/MAS spectra were obtained compacting the solid samples inside a 4 mm diameter rotor and the experiments were conducted using a PA BBO 600 S3 BBF-H-D-05 A SP head-probe, with a rotation frequency of 10 kHz. The contact time and recycle delay were 3 s each.

## Results and Discussion

The detailed synthesis and full characterization of this series of pyrimidinones have already been reported and described elsewhere.^47−80^ However, the Experimental Section, Scheme 1, depicts the general reaction scheme for the synthesizing the pyrimidinones used in this work.

Initially, crystallization assays were conducted by slow solvent evaporation. Previous studies have indicated the low solubility of these compounds in various organic solvents. Consequently, a screening was carried out using commonly employed organic solvents to determine solubility, which would facilitate future solution-based experiments (e.g., NMR spectroscopy). The objective was also to obtain crystals in different crystalline forms and phases, including solvates and polymorphs.

It was found that most of the studied pyrimidinones were soluble in acetonitrile and acetone and slightly soluble in other solvents, such as chloroform, dichloromethane and dimethylformamide. Despite their low solubility, crystal formation was not impeded in these solvents, resulting in the formation of either small crystalline quantities or small crystals. In most of the solvents evaluated, single crystals were formed. The highest quality crystals, as assessed macroscopically and confirmed via microscopy, were selected for diffraction. The crystallographic data employed were those with the fewest crystallographic alerts, as elucidated by the Checkcif software.^67^

Furthermore, we noted that these compounds do not tend to form solvates under the crystallization conditions used (i.e., slow evaporation at 25 °C), even in polar solvents and those that typically form hydrogen bonds, such as water or dimethyl sulfoxide. Moreover, although crystals were formed in different solvents, the crystal structure and structural conformation of the compounds remained consistent regardless of the solvent used for crystallization. Detailed information about the assays conducted with a range of solvents and the resulting crystals for all compounds is provided in the SI.

**Figure 2:**
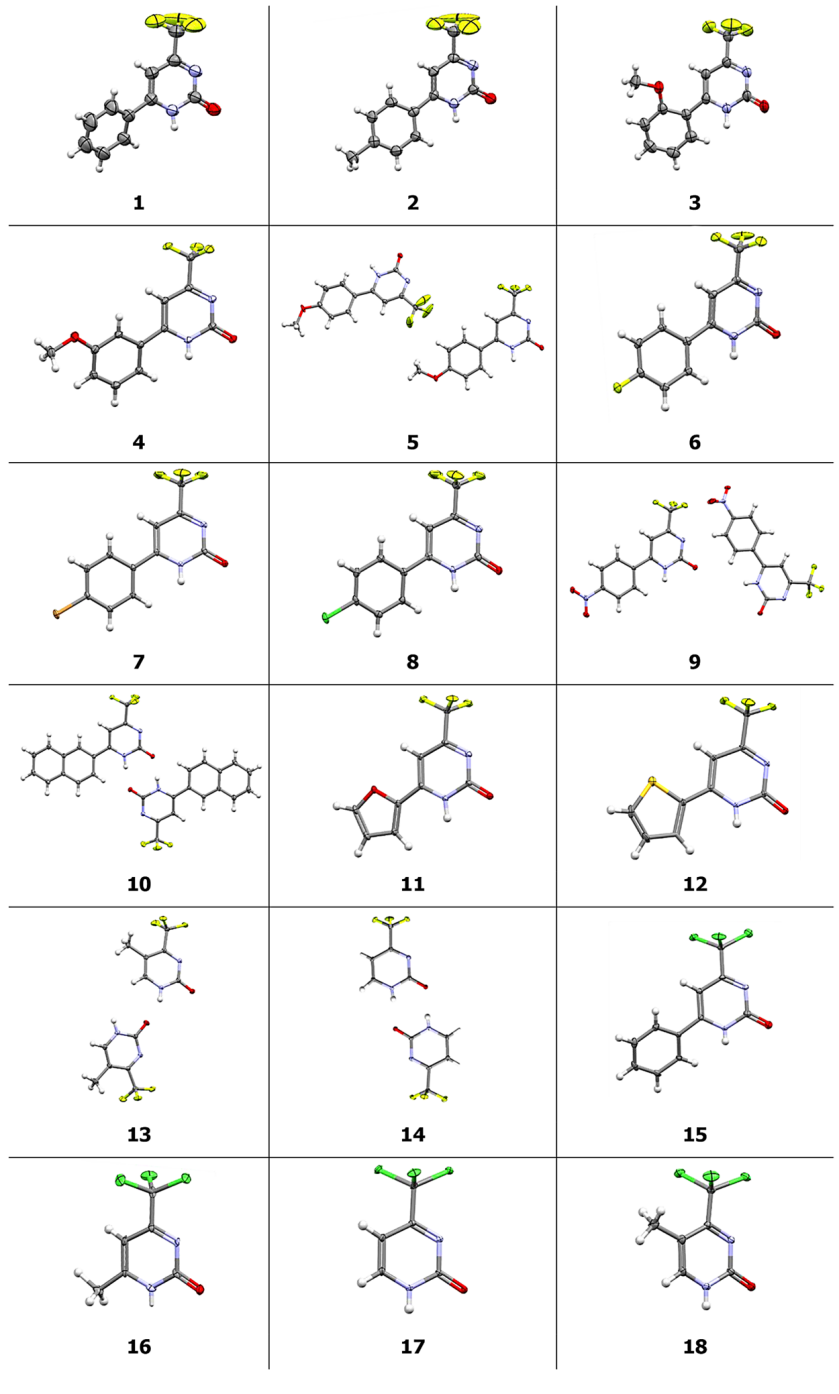
Thermal displacement ellipsoids of structures **1**–**18** at the 50% probability level. The hydrogen atoms are represented by spheres with arbitrary radii.

Crystals suitable for SC-XRD were obtained for all 18 compounds. The corresponding thermal ellipsoids are displayed in Figure 2. The crystallographic data and structural refinement parameters are summarized in Tables S2 and S3 in the SI. Most of the crystals exhibit only one structure in the asymmetric unit: *Z*′ = 1, namely compounds **1**–**4**, **6**–**8**, **11**, **12**, **17**, and **18**. The remaining compounds, **5**, **9**, **10**, **13**, and **14**, contain two independent structures in the asymmetric unit, *Z*′ = 2. This phenomenon in crystallography is known as conformational isomorphism.^64,68,69^ To distinguish between the two conformations, the nomenclatures A and B were adopted, for instance, **5A** and **5B** for the two different conformations of compound **5**. Details of all conformational isomorphs are provided in the SI.

**Figure 3:**
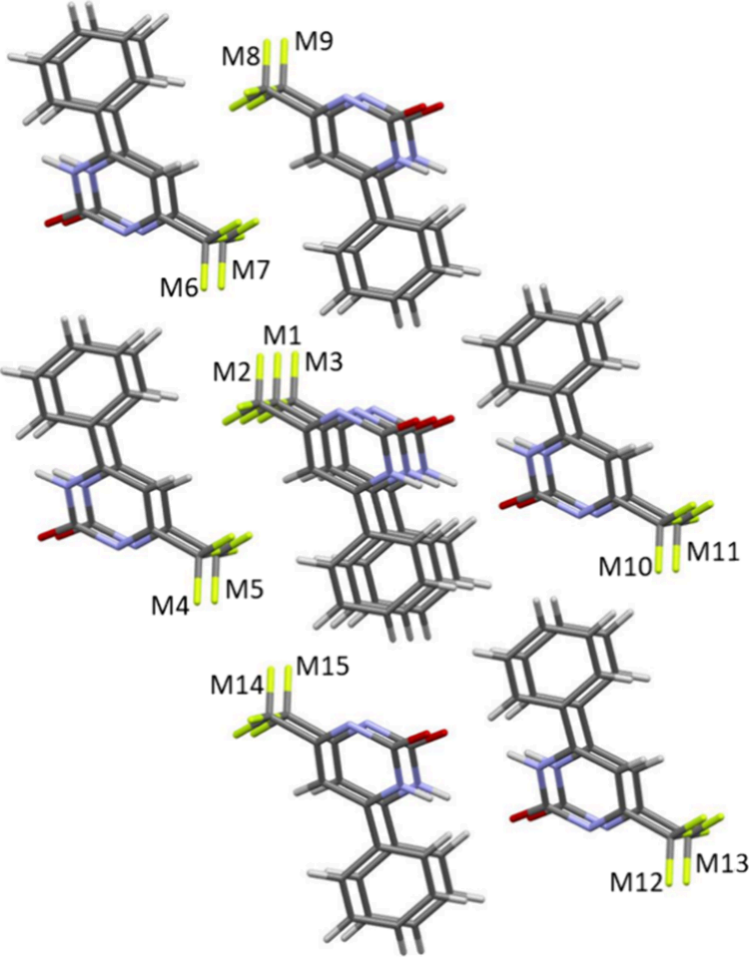
Supramolecular cluster for compound **1**.

By using SC-XRD data, we sought to understand the supramolecular organization of these compounds in the crystalline solid state; this was achieved by employing the supramolecular cluster as a demarcation tool.^40,70−72^ The supramolecular cluster involves identifying the smallest portion of the crystal lattice that contains all the information regarding the stabilization energy between molecules and their topological complementarity. For compounds featuring more than one structure in the asymmetric unit, two supramolecular clusters were demarcated from each of the independent molecules, following the method previously reported by our research group.^36,64,70,73^ Furthermore, in addition to the computational methodology, complementary experiments in saturated solutions using CD-NMR were conducted to bolster our findings derived from the supramolecular clusters data.^39,57,69^ As an illustrative example, compound **1** was chosen to demonstrate a supramolecular cluster (Figure 3), while the supramolecular clusters pertaining to the remaining 17 compounds can be found in the SI.

Based on our hypothesis that the initial nuclei formed in solution persist and permeate the crystallization process, we utilized SC-XRD data to track and quantify these nuclei. By applying the fundamentals of retrocrystallization and employing the supramolecular cluster as a demarcation tool, we assessed the stability of these nuclei in terms of the stabilization energies between molecules and their contact areas. Consequently, we propose a plausible pathway via a crystallization mechanism.

Once we obtained the SC-XRD data, we initiated an analysis of how the crystal formed by examining the crystal lattice in conjunction with data from the supramolecular clusters. Given that nearly all organic molecules do not have identical contact area with all neighboring molecules, and, consequently, do not interact with the same stabilization energy with all of them, a set of parameters was delineated from the ideal contribution of 1/*N* of the stabilization energy and 1/*N* of the contact area. As NG_M1···M*N*_ and NC_M1···M*N*_ represent the normalized stabilization energy and the normalized contact area, respectively, in a supramolecular cluster with *N* molecules, the robustness of the interactions between M1 and any molecule M*N* can be estimated from the contributions of these normalized parameters.^40^

**Figure 4:**
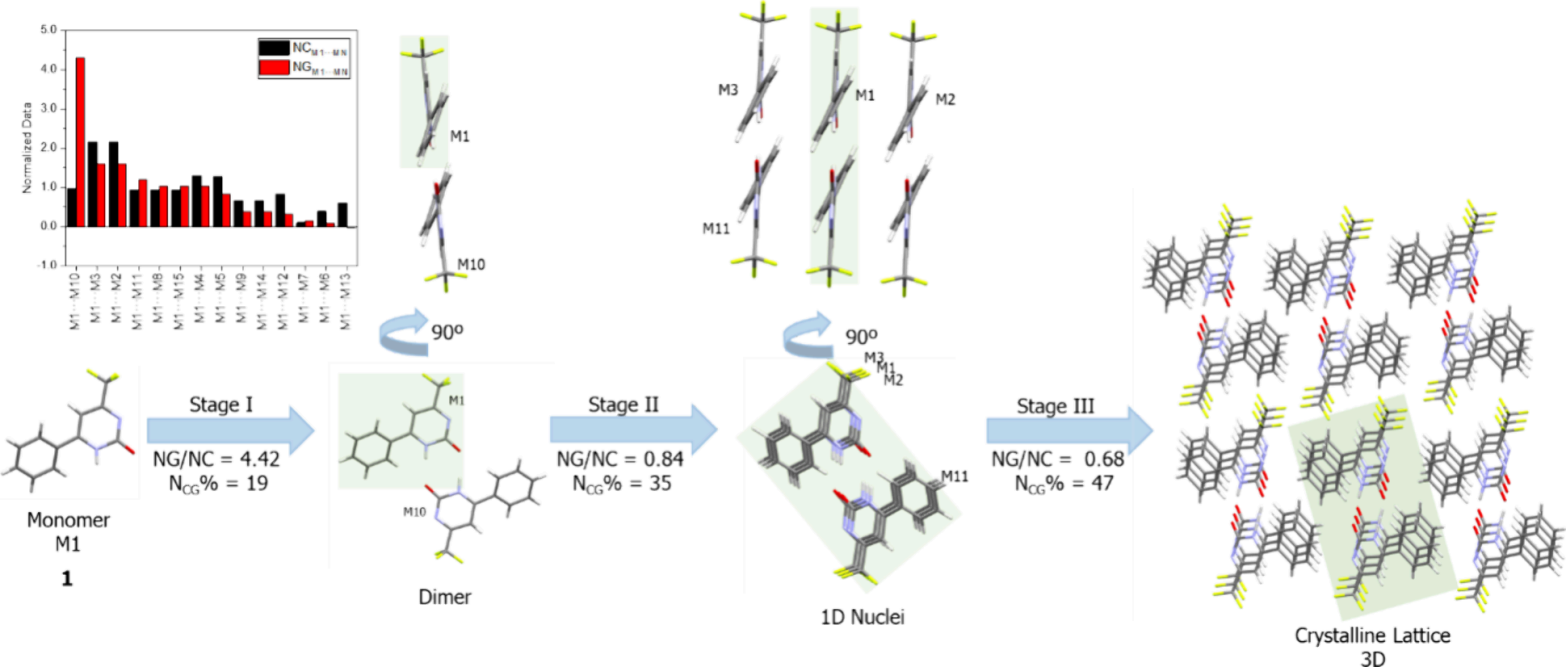
Proposed crystallization mechanism for compound **1**. The shaded area highlights the portion of the previous step.

Normalizing all the data allows for verifying and identifying which molecular aggregates or dimers are most likely to form first and quantifying each aggregate’s contribution to crystal formation. Aggregates that are more energetically stable and topologically complementary tend to form initially and persist in the crystallization process. To elucidate this approach, we continued with compound **1** as an example. Figure 4 displays the proposed crystallization mechanism based on normalized data.

An analysis of the normalized bar graph (highlighted in red and black in the upper left corner) reveals that one dimer, formed with the M10 molecule, is prominent. Energetically, this dimer stands out due to a ‘possible’ double hydrogen bond formed by the pyrimidinone core. This intermolecular interaction contributes to a high-energy stabilizing factor and a more nuanced contact area. Therefore, it is very likely this dimer is the first to form in solution (Stage I, Figure 4). Additional parameters, such as NG/NC and *N*_CG_%, leverage the normalized data to elucidate which features dominate the stage and the percentage contribution of the stage to crystal formation.^60,64,71,72^

The first step is predominantly governed by the stabilization energy factor attributable to the double hydrogen bond, accounting for 19% of the crystal formation. Continuing our systematic analysis, we observed that dimers M2 and M3 are significant. By analyzing the crystal lattice, we can infer that dimers analogous to the one formed by M1···M10 aggregate, implying that M2 and M3 approach similar dimers; subsequently, M3···M11 and M2···M*N* come together, forming a one-dimensional block (Stage II). This stage is characterized by the topology of the molecules (NG/NC = 0.84) and has an *N*_CG_% value of 35. Ultimately, when other one-dimensional nuclei like this one are formed, they converge and contribute to the formation of the crystal lattice, depicted in Stage III. Typically, this final step is frequently governed by molecular topology.

In our pursuit of aligning computational insights with experimental observations, we conducted CD-NMR experiments on compound **1**. This technique offers a window into the intermolecular interactions within a solution, thereby validating the proposed stages in our crystallization mechanisms.^74−78^ By simulating the initial stages of crystallization in a saturated solution, CD-NMR mimics the conditions where the solute concentration surpasses that of the solvent, allowing nuclei to form and persist during molecular self-assembly. Consequently, these nuclei cooperate and adapt throughout the crystallization process. The CD-NMR experiment enables the observation of changes in the chemical shifts of certain peaks in the spectra, providing crucial insights into the underlying molecular dynamics.

**Figure 5:**
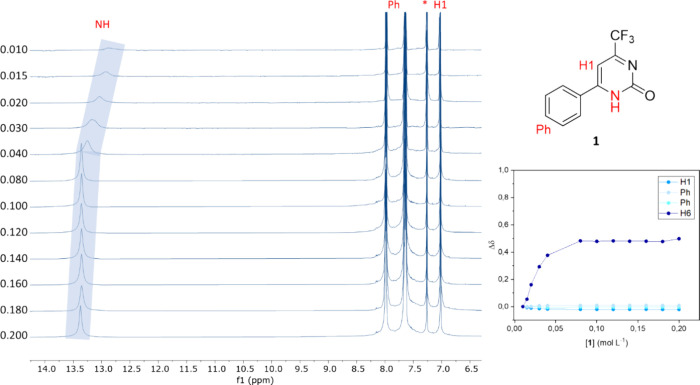
^1^H NMR experiments with variable concentrations of compound **1** were carried out in CDCl_3_ at 298 K, and the correlation between the Δδ of the H1, phenyl hydrogens and the N–H of the pyrimidinone.

In the case of compound **1** (Figure 5), the phenyl substituent at position 6 of the pyrimidinone ring and the hydrogen at position 5 did not undergo any significant change, and their peaks remained stable throughout the experiment. Conversely, the N–H moiety positioned at position 1 of the pyrimidinone ring displays notable changes with varying concentrations. Specifically, the peaks show a deshielding trend as the concentration increases, as depicted from top to bottom in the spectra of Figure 5. This behavior strongly suggested the formation of a hydrogen bond in solution.^76^ To further elucidate this phenomenon, the change in chemical shift is graphically depicted in the Δδ × [**1**] plot within Figure 5, providing a clearer visualization of the observed variations.

**Figure 6:**
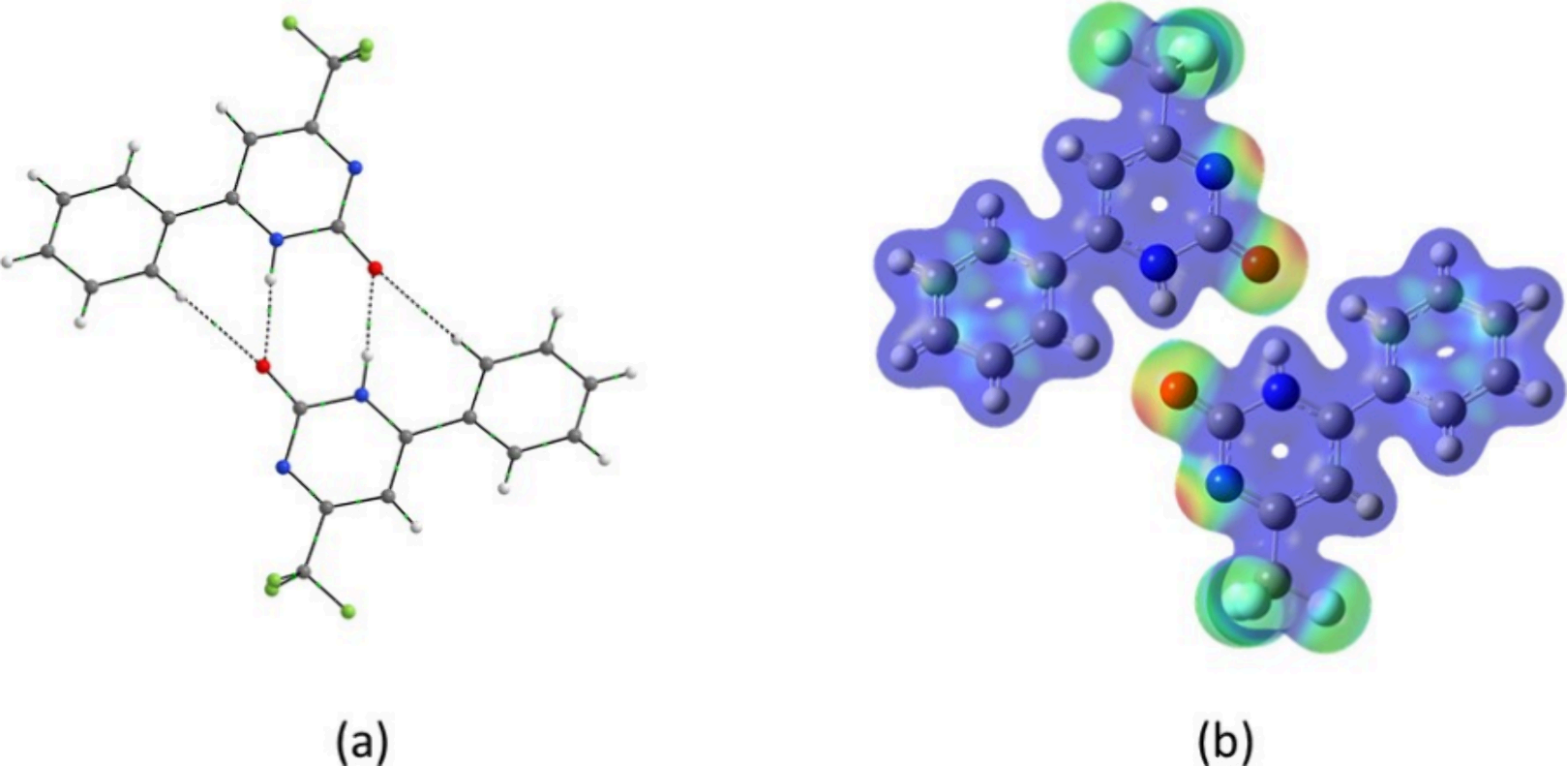
Dimer M1···M10 of compound **1**. (a) Intermolecular interaction data obtained by QTAIM, (b) MEP surface: red and blue with −0.07 and 0.09 au, respectively.

The information obtained from the CD-NMR experiments allowed us to closely examine the intermolecular interactions within the M1···M10 dimer, confirming the existence of the hydrogen bond in the crystalline solid state as well. To achieve this objective, we conducted a *G*_AI_ analysis and calculated its MEP surface area. The *G*_AI_ analysis unveiled two N–H···O and two C–H···O interactions, contributing a total energy of −21.72 kcal mol^–1^, which accounts for 19% of the overall stabilization energy of the crystal (Figure 6). Upon analyzing each interaction, it was established that the two N–H···O interactions had a stabilization energy of −16.50 kcal mol^–1^, while the C–H···O interactions contributed with −5.22 kcal mol^–1^. The MEP surface analysis of the dimer demonstrated electrostatic complementarity between the positive (displayed in red) and negative (displayed in blue) parts of the monomers’ surface.

The *G*_AI_ analysis revealed that the first nucleus formed during the crystallization process is a dimer with relatively strong intermolecular interactions, primarily governed by hydrogen bonds. Qualitatively, we observed this phenomenon was visualized on the MEP surface. Thus, the crystallization mechanism, which begins with the formation of dimers, was deemed plausible, supporting both the experimental data in solution and the computational calculations.

**Figure 7:**
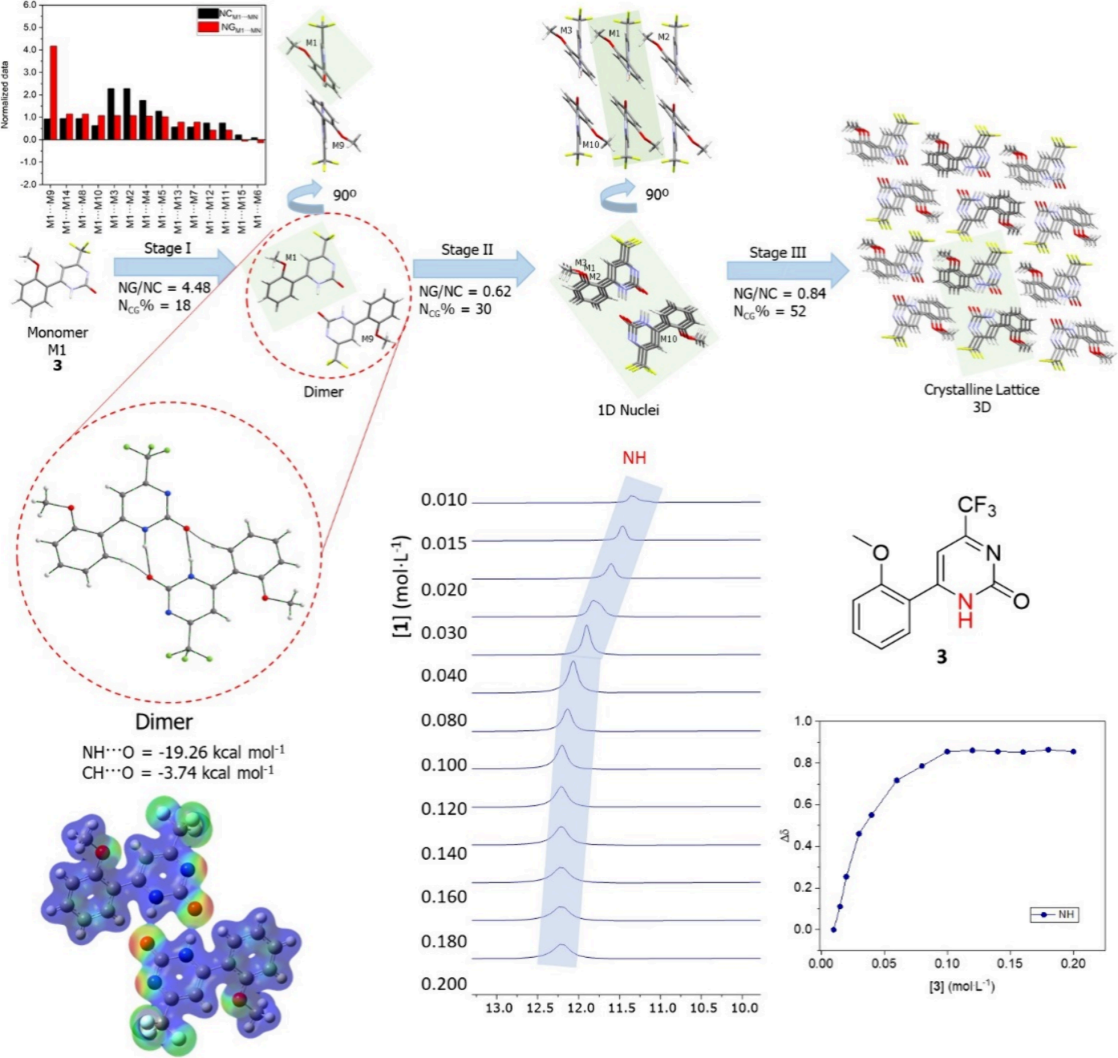
Proposed crystallization mechanism of compound **3**, with the shaded area highlighting the portion described in the preceding step. The *G*_AI_ analysis, in this case, primarily focus on the first stage — dimer formation — and elucidates the strength of the hydrogen bond and identify the atoms involved. CD-NMR experiments, performed in CDCl_3_ at 298 K, disclosed the association between the Δδ of the N–H hydrogen in pyrimidinone. The MEP surface is depicted in red and blue, with −0.07 and 0.09 au, respectively.

This approach was then applied to the other molecules in this study. However, due to the low solubility in deuterated and low-polarity aprotic solvents, such as CDCl_3_, only a few compounds could confirm dimer formation in solution. For instance, compound **3** exhibits behavior analogous to compound **1** in terms of the proposed crystallization mechanism, CD-NMR experiments, and the correlation with the QTAIM data, *G*_AI_ analysis, and MEP data (Figure 7).

We observed a trend where, as the concentration increased, the hydrogen peak of the N–H in the pyrimidinone ring shifted away from TMS, resulting in signal deshielding. Notably, the C–H···O interaction was not observed in the CD-NMR experiments. Additional experiments conducted on other compounds in this study can be found in the SI.

**Figure 8:**
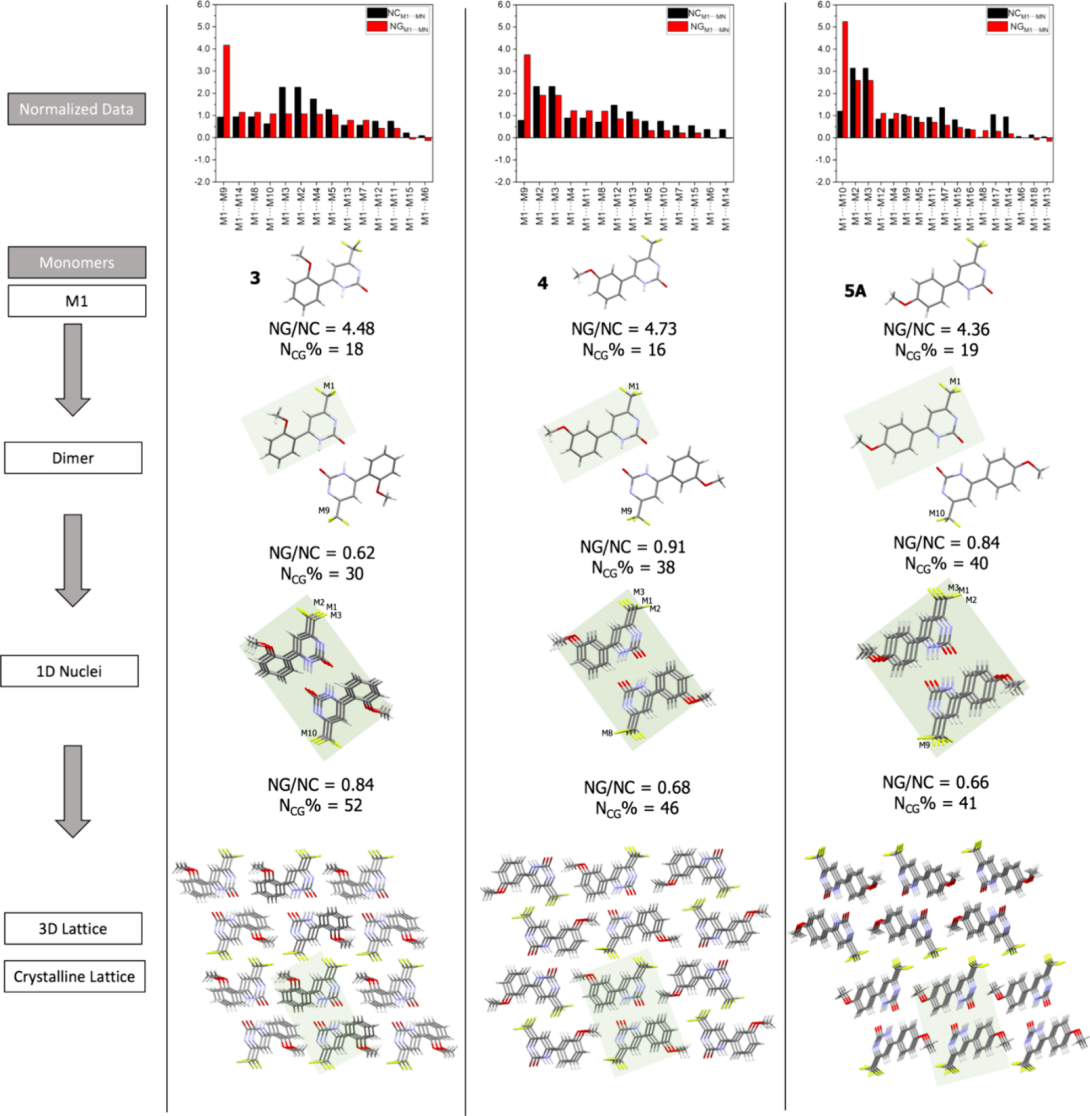
Proposed crystallization mechanism for compounds **3**, **4,** and **5A**. The shaded area highlights the portion of the previous step.

Another observation was related to the proposed crystallization mechanisms. When the substituents at positions 5 and 6 of the ring are modified, the mechanism involves the initial formation of a dimer, followed by the approach of analogous dimers to form one-dimensional blocks. These blocks then approach each other and stabilize topologically, culminating in crystal formation. Although the NG and NC values changed with different substituents, the proposed mechanism remained consistent based on these data. This is illustrated in Figure 8 for compounds **3**, **4**, and **5A**, where position 5 has a hydrogen atom and position 6 has a MeOPh as a substituent. The MeO group was introduced at positions 2, 3, and 4 positions of the phenyl ring.

**Figure 9:**
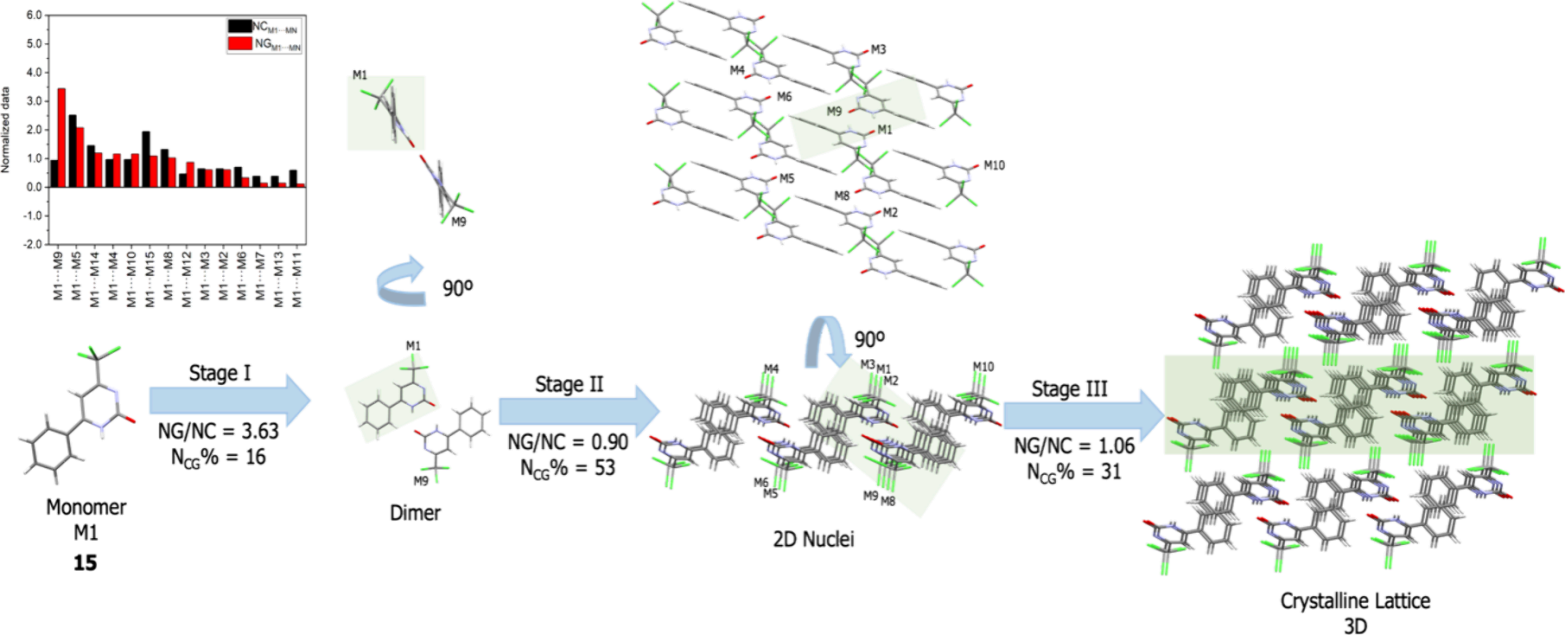
Proposed crystallization mechanism for compound **15**. The shaded area highlights the portion of the previous step.

However, when the halomethyl substituent at position 4 is modified, transitioning from CF_3_ to CCl_3_, there is a significant alteration in the normalized data values from the supramolecular clusters, leading to a different conclusion regarding the crystallization mechanisms proposals. To exemplify, Figure 9 shows the proposed mechanism for compound **15**. Compound **15** differs from compound **1** only in the halogen atoms at position 4, yet the values of intermolecular stabilization energies and contact areas exhibit notable variations.

The proposed mechanism for compound **15** also initiates with the formation of a dimer, driven by a double hydrogen bond, hence, strongly influenced by energetic factors. Previously, the formation of a one-dimensional dimer was observed in the first stage. Nonetheless, in this instance, we observe the emergence of a two-dimensional block governed by topological factors. This ultimately culminates in the formation of the crystal, comprising analogous two-dimensional blocks. This phenomenon was similarly observed for compounds **16**–**18**, which also feature CCl_3_ at position 4. All proposed crystallization mechanisms are detailed in the SI.

**Figure 10:**
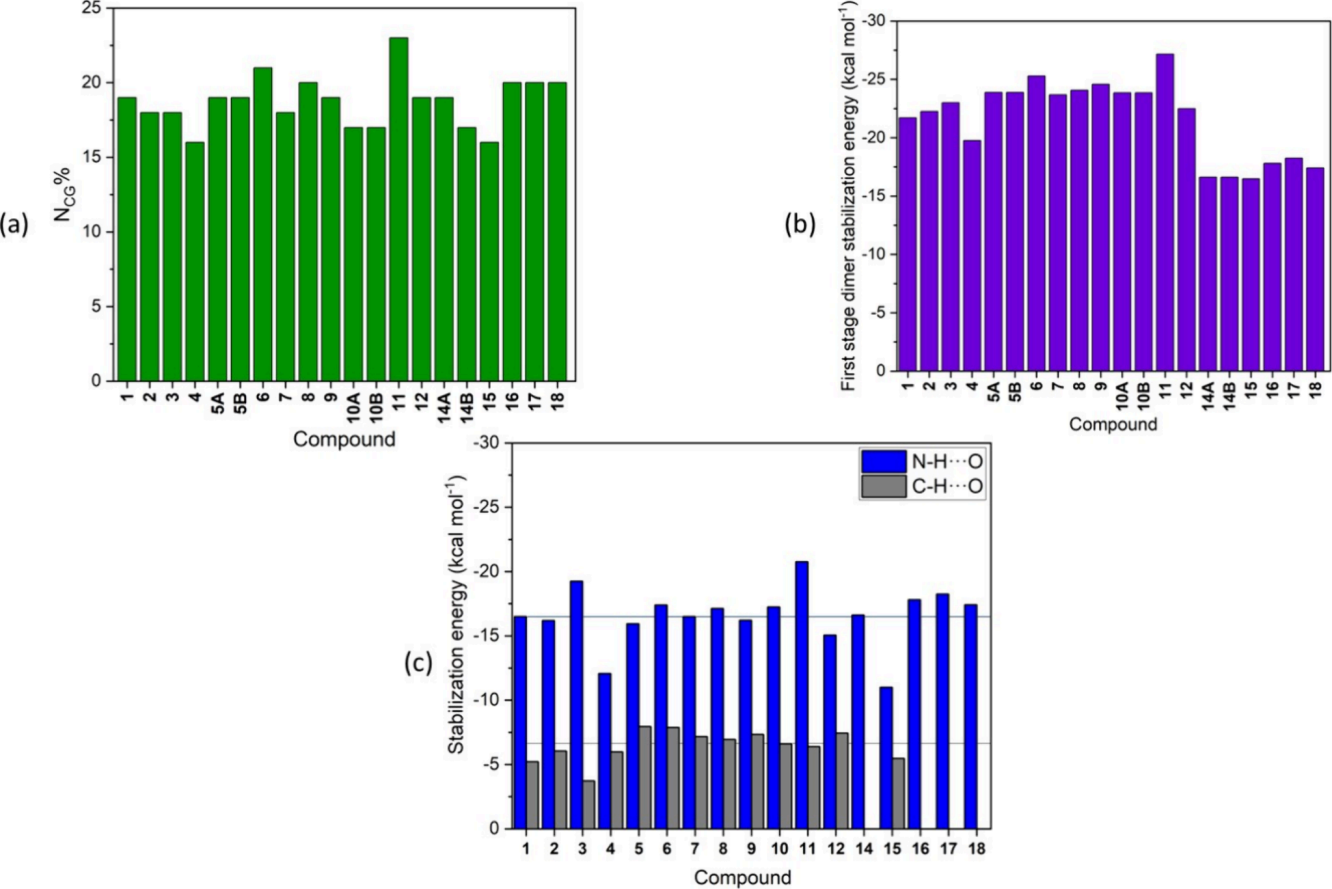
Bar graphs detailing the hydrogen bonds in compounds **1**–**18**. (a) *N*_CG_% contribution of the initial dimer of each supramolecular cluster, demonstrating the extent to which hydrogen bonding contributes to the assemble of the crystal; (b) raw stabilization energy of first stage dimer in kcal mol^–1^; (c) interactions present in the hydrogen bonds of each compound. The lines represent the mean values of the interactions, dark blue for N–H···O and gray for C–H···O, respectively.

Instigated by the hydrogen bonding of the initial dimers, each compound underwent *G*_AI_ analysis, confirming the robustness of hydrogen bond formation, independent of the substituents present. Figure 10 presents three bar graphs that provide details about the hydrogen bonds in the models studied. Figure 10a exhibits the amount, in *N*_CG_%, of the energetic contribution of the hydrogen bond in terms of intermolecular stabilization energies, and, topologically, in relation to the contact area. This percentage reflects the extent to which these intermolecular interactions contribute to the overall construction of the crystal. On average, the contribution from just two molecules is 19%, reaching remarkable values as high as 23% of crystal formation. In the raw data, this value equates to approximately −27.17 kcal mol^–1^, as for compound **11** (Figure 10b). Figure 10c shows this hydrogen bonding in a fragmented manner, presenting the types of interactions and the atoms involved in the interactions based on *G*_AI_ analysis data. Notably, the N–H···O interactions display exceptionally high values, with an average of −16.55 kcal mol^–1^. However, the C–H···O interactions also contributes considerably to these systems (an average of −6.48 kcal mol^–1^), a factor that cannot and should not be disregarded. The C–H···O was not considered for compounds **14,** and **16**–**18,** due to the nature of their respective substituents at position 6, which makes the presence of this interaction unfeasible.

Compounds **3**, **4**, and **5** were used as examples for solid-state NMR experiments, contrasting with solution NMR experiments. Given that compound **5** exhibits a *Z*′ = 2, it is sometimes feasible to observe multiple signals for the equivalent nucleus when the magnetic environments of chemically identical nuclei diverge sufficiently to cause an observable change in the NMR spectrum.^80,64,79^ Given that, compounds **3**, **4**, and **5** share a similar substituent at position 6, MeOPh, the impact of altering the position of the MeO group (at position 2, 3, or 4) within the Ph group at position 6 of the pyrimidinone ring on the chemical shift, was explored both in solution and in the solid state. Figure S72 delineates these spectra in solution and in the solid state.

As observed, the most pronounced shifts occurred in the region where sp^2^ carbons are typically located. For compound **3**, bearing the MeO group at position 2, four distinct carbon signals were expected, and indeed, the expectation was met, with four distinct carbons signals emerging in this region both in solution and in the solid state. A similar outcome was observed for compound **4**. In the case of compound **5**, which possesses conformational isomers, two sp^2^ carbon signals were observed in solution. Nevertheless, in the solid state, four peaks appeared, likely attributable to the presence of four carbons in different magnetic environments, prompting discernible changes. Regrettably, sidebands in the solid-state spectra, coupled with impurities possibly stemming from the decomposition of molecules in solution, obscured our observations and hampered definitive conclusions.

**Figure 11:**
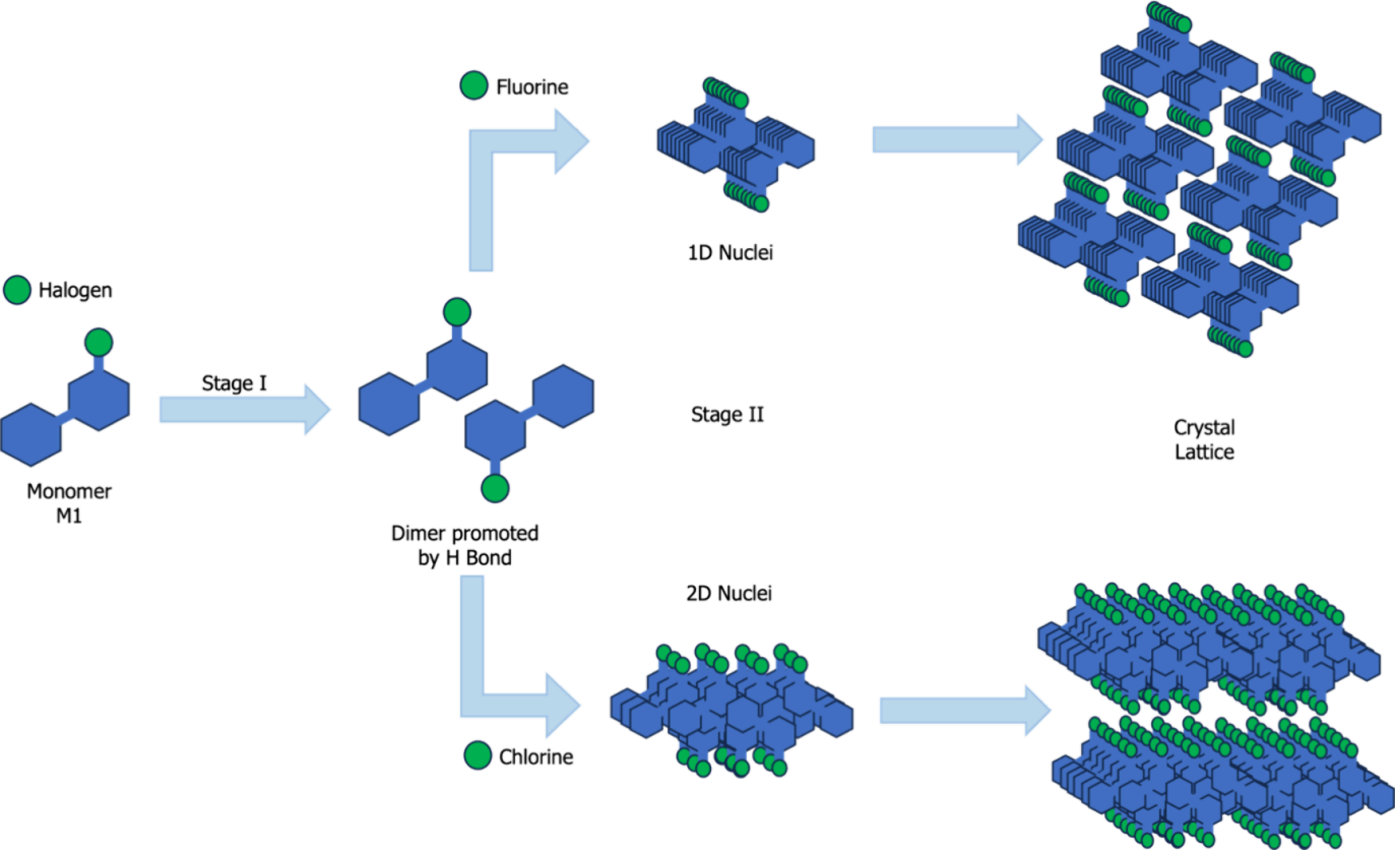
Proposed crystallization mechanisms associated with the changes in halogen atoms.

The pyrimidinone models investigated herein displayed distinct profiles and proposed crystallization mechanisms, primarily influenced upon the halogen substituent at position 4 of the pyrimidinone ring. The proposed crystallization mechanisms are summarized in Figure 11 for a comprehensive understanding. At their core, both mechanisms commence with the formation of a dimer in the initial stage, highlighting the persistence of the hydrogen bond in these models. Following this, the variation in the halogen leads to a change in the proposed mechanism. For pyrimidinones with fluorine substituent at position 4, similar dimers approach and stabilize, successively forming a one-dimensional block. These one-dimensional blocks then aggregate, culminating in the formation of a crystal. Conversely, for pyrimidinones with chlorine substituent at position 4, the creation of a dimer in Stage I is succeeded by stabilization through the formation of a two-dimensional block. These two-dimensional blocks subsequently aggregate to form the crystal.

## Conclusions

This study explored the supramolecular environment both in the crystalline solid state and in solution for a series of 18 4-(trihalomethyl)-2(1*H*)-pyrimidinones molecules with different substituents at positions 4, 5, and 6 of the pyrimidinone ring. Crystals suitable for SC-XRD were successfully obtained for each compound studied, resulting in 18 new crystalline phases.

By applying the demarcation of the supramolecular cluster, we conducted a study to propose crystallization mechanisms. It was observed that hydrogen bonds play a crucial role in the intermolecular stabilization of the initial dimers formed, which in turn guides the crystallization process. The recurrent robust hydrogen bond contributes to approximately 19% of the total crystal formation, on average, a significant figure for just one dimer. The initial dimers were also confirmed through concentration-dependent NMR experiments. Simulating saturated solutions similar to those in the crystallization process, the formation of the dimers led to significant shifts in the NMR spectra, indicative of hydrogen bond formation in solution.

A comparison of the proposed crystallization mechanisms revealed that replacing the halogen at position 4 of the pyrimidinone ring (F/Cl) alters the profiles of the proposed crystallization mechanisms. Pyrimidinones containing fluorine undergo dimer formation, followed by the formation of a one-dimensional block, whereas pyrimidinones with chlorine at the same position proceed from dimer formation to the creation of a two-dimensional block.

Lastly, the initial dimers were evaluated using QTAIM and *G*_AI_ analysis to determine the predominant intermolecular interactions and their significance. We observed that the persistence of the hydrogen bond remains irrespective of the substituents at positions 4, 5, or 6. The primary and most energetically significant interaction between the dimers is N–H···O, with an average value of −16.55 kcal mol^–1^, followed by the C–H···O interaction, with an average of −6.48 kcal mol^–1^.

We hope our findings can guide researchers exploring the properties of pyrimidinones and their therapeutic effects. The parallels with the nitrogenous bases of DNA and RNA underscore the wider relevance of hydrogen bonds beyond mere structural aspects. Therefore, understanding the nature and robustness of these intermolecular interactions is crucial for the effective and strategic application of pyrimidinones and other similar molecules.

## Data Availability

The data underlying this study are available in the published article and its Supporting Information.

## Supporting Information Available

General synthesis information, crystallographic data, supramolecular cluster data (contact area and stabilization energy), proposals for crystallization mechanism, QTAIM data, MEP surfaces, and CD-NMR Data (PDF)
