# Supplementary material for: The Persistence of Hydrogen Bonds in Pyrimidinones: From Solution to Crystal
Source: ACS Org Inorg Au. 2024 Sep 5;4(5):557–70. doi: 10.1021/acsorginorgau.4c00057 (PMC11450830; doi:10.1021/acsorginorgau.4c00057)
Supplement: Supplementary file 1 — gg4c00057_si_001.pdf [file gg4c00057_si_001.pdf]

SUPPORTING INFORMATION

The Persistence of Hydrogen Bonds in Pyrimidinones: From Solution to Crystal

Fellipe F. S. Farias[a,*], Mateus Mittersteiner[a], Amanda M. Kieling[a], Priscila S. V. Lima[a], Gustavo H. Weimer[a], Helio G. Bonacorso[a], Nilo Zanatta[a] and Marcos A. P. Martins[a,*]

[a]*Núcleo de Química de Heterociclos* (NUQUIMHE), Department of Chemistry, Federal University of Santa Maria (UFSM), 97105-900, Santa Maria, RS, Brazil.

*Corresponding authors: fellipefreire@hotmail.com ; marcos.nuquimhe@gmail.com

Summary

1. GENERAL SYNTHESIS REACTION SCHEME 1
2. CRYSTALLOGRAPHIC DATA 2
3. CONFORMATIONAL ISOMORPHS 7
4. SUPRAMOLECULAR CLUSTER DATA 9
5. CRYSTALLIZATION MECHANISMS 42
6. QTAIM DATA 52
7. MEP SURFACES 61
8. CD NMR DATA 64
9. SOLID STATE NUCLEAR MAGNETIC RESONANCE 68
10. THERMAL DISPLACEMENT ELLIPSOIDS 68

1. General synthesis reaction scheme

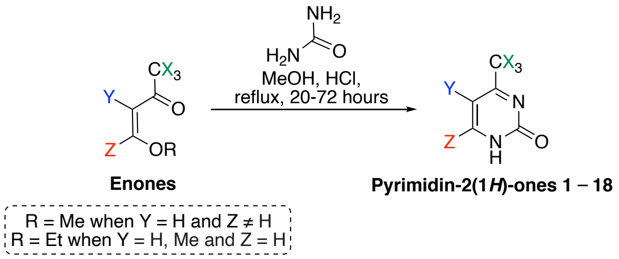

Scheme S1. General reaction scheme for the formation of the pyrimidinones used in this work.

2. Crystallographic data

Table S1. Crystallization assays with different solvents. Marked with an X are the solvents where single crystals were observed and in **X** (in bold and red) was the one that presented the best crystals and finest crystallographic data, these data were deposited in the CCDC and used in this study.

C \ S	MeCN	MeOH	$CHCl_3$	DCM	DMF	DMSO	THF	H_2O	Ace	EtOAc	$PhNH_2$	EtOH	IPA	Cy	PEE	Et_2O	Hx	Py	Tol
**1**	**X**	X	X	X	X	X	X		X			X						X	
**2**	**X**			X	X	X	X	X			X								
**3**	**X**		X	X			X		X				X			X			
**4**	**X**			X	X	X	X	X	X	X			X	X				X	
**5**	X	X	X	X	**X**		X		X	X			X					X	
**6**	**X**	X			X	X	X	X	X		X	X	X	X		X			
**7**	X	X	X	X		X		X		**X**			X	X		X		X	
**8**	X	**X**							X										
**9**	X		**X**			X			X										
**10**	X	X			**X**		X		X										
**11**	X	X	X				**X**		X		X			X					
**12**	**X**		X	X	X								X	X	X				
**13**	**X**	X							X			X	X						
**14**	X	**X**			X	X					X							X	
**15**	**X**		X	X					X	X						X			
**16**	X	X		**X**	X	X		X	X									X	
**17**	X	**X**					X					X	X		X	X			
**18**	**X**	X		X			X		X	X			X			X		X	

C = compound; S = solvent. MeCN = Acetonitrile; MeOH = Methanol; $CHCl_3$ = Chloroform; DCM = Dichloromethane; DMF = Dimethylformamide; DMSO = Dimethyl Sulfoxide; THF = Tetrahydrofuran; H2O = Water; Ace = acetone; EtOAc = Ethyl Acetate; $PhNH_2$ = Aniline; EtOH = Ethanol; IPA = Isopropyl alcohol; Cy = Cyclohexane; PEE = Petroleum ether; Et_2O = Diethyl ether; Hx = Hexane; Py = Piridine; Tol = Toluene.

Table S2. X-ray data collection and structure refinement parameters for compounds **1–9**.

Compound	**1**	**2**	**3**	**4**	**5**	**6**	**7**	**8**	**9**
CCDC Number	2359091	2359092	2359093	2359095	2359094	2359096	2359097	2359103	2359102
Formula	$C_{11}H_7F_3N_2O$	$C_{12}H_9F_3N_2O$	$C_{12}H_8F_3N_2O_2$	$C_{12}H_9F_3N_2O_2$	$C_{12}H_9F_3N_2O_2$	$C_{11}H_6F_4N_2O$	$C_{11}H_6BrF_3N_2O$	$C_{11}H_6ClF_3N_2O$	$C_{11}H_6F_3N_3O_3$
Formula weight	240.19	254.21	269.205	270.21	270.21	258.18	319.09	274.63	285.19
Temperature (K)	293.14	297.38	297.58	100.00	100.0	100.0	100.00	100.04	100.01
Crystal system	Triclinic	Monoclinic	Monoclinic	Monoclinic	Monoclinic	Monoclinic	Triclinic	Triclinic	Monoclinic
Space group	P-1	C2/c	C2/c	$P2_1/n$	$P2_1$	C2/c	P-1	P-1	$P2_1/c$
a (Å)	4.7971(14)	30.299(11)	26.510(9)	4.5955(13)	5.0342(14)	29.335(12)	4.8681(2)	4.7892(14)	8.6250(3)
b (Å)	9.062(4)	4.8977(12)	5.017(2)	12.923(4)	25.383(10)	4.9767(13)	8.8719(4)	8.719(3)	18.1540(7)
c (Å)	13.014(3)	17.324(8)	18.117(7)	19.020(6)	8.992(3)	15.132(5)	12.9809(5)	13.021(4)	14.2994(5)
α (°)	95.30(3)	90	90	90	90	90	90.2150(10)	92.481(11)	90
β (°)	90.349(19)	120.25(3)	103.083(13)	90.680(10)	96.521(10)	118.816(17)	94.1530(10)	93.045(13)	96.8580(10)
γ (°)	113.15(2)	90	90	90	90	90	99.6640(10)	99.397(7)	90
Cell volume ($Å^3$)	517.4(3)	2220.25(3)	2346.9(16)	1129.4(6)	1141.6(7)	1935.6(12)	551.16(4)	534.9(3)	2222.96(14)
Z	2	8	8	4	4	8	2	2	8
Calculated density ($g\ cm^{-3}$)	1.542	1.521	1.524	1.589	1.572	1.772	1.923	1.705	1.704
μ (mm^{-1})	1.213	0.133	0.137	0.142	0.141	0.169	3.758	0.387	0.159
F(000)	244.0	1040.0	1097.1	552	552	1040.0	312.0	276.0	1152.0
Crystal size (mm^3)	0.427×0.349×0.224	0.915×0.085×0.06	1.04×0.281×0.175	0.554×0.149×0.07	0.466×0.217×0.114	0.354×0.168×0.134	0.403×0.161×0.063	0.235×0.113×0.108	0.438×0.284×0.218
Wavelength (Å)	Cu Kα (λ = 1.54178)	Mo Kα (λ = 0.71073)	Mo Kα (λ = 0.71073)	Mo Kα (λ = 0.71073)	Mo Kα (λ = 0.71073)	Mo Kα (λ = 0.71073)	Mo Kα (λ = 0.71073)	Mo Kα (λ = 0.71073)	Mo Kα (λ = 0.71073)
θ range for data collection (°)	6.828 to 136.866	4.716 to 54.322	4.62 to 54.28	4.284 to 54.196	4.56 to 54.224	5.388 to 54.244	4.658 to 54.396	4.742 to 56.646	4.488 to 56.64

Index ranges	-5 ≤ h ≤ 5, -10 ≤ k ≤ 10, -15 ≤ l ≤ 15	-38 ≤ h ≤ 36, -5 ≤ k ≤ 6, -21 ≤ l ≤ 22	-32 ≤ h ≤ 32, -6 ≤ k ≤ 6, -23 ≤ l ≤ 20	-5 ≤ h ≤ 3, -16 ≤ k ≤ 16, -23 ≤ l ≤ 23	-4 ≤ h ≤ 6, -31 ≤ k ≤ 32, -11 ≤ l ≤ 11	-37 ≤ h ≤ 36, -4 ≤ k ≤ 6, -17 ≤ l ≤ 19	-5 ≤ h ≤ 6, -11 ≤ k ≤ 11, -16 ≤ l ≤ 16	-6 ≤ h ≤ 4, -11 ≤ k ≤ 11, -17 ≤ l ≤ 15	-11 ≤ h ≤ 11, -21 ≤ k ≤ 24, -19 ≤ l ≤ 19
Collected reflections	7045	19273	17289	11659	18886	19163	17644	13867	52804
Independent reflections	1809 [R_{int} = 0.0255, R_{sigma} = 0.0220]	2392 [R_{int} = 0.0644, R_{sigma} = 0.0750]	2508 [R_{int} = 0.0543, R_{sigma} = 0,0557]	2169 [R_{int} = 0.0389, R_{sigma} = 0.0418]	4510 [R_{int} = 0.0467. R_{sigma} = 0.0489]	2090 [R_{int} = 0.0526, R_{sigma} = 0.0395]	2440 [R_{int} = 0.0241, R_{sigma} = 0.0146]	2319 [R_{int} = 0.0463, R_{sigma} = 0.0476]	5522 [R_{int} = 0.0375, R_{sigma} = 0.0214]
Data/restraints/parameters	1809/0/158	2392/0/168	2508/0/173	2169/0/177	4510/1/415	2090/0/167	2440/0/163	2319/0/167	5522/0/361
Goodness-of-fit (F^2)	1.136	1.106	1.214	1.106	1.061	1.140	1.074	1.043	1.030
R_1 (I > 2σ(I))	R_1 = 0.0722, wR_2 = 0.1999	R_1 = 0.1206, wR_2 = 0.3062	R_1 = 0,0622, wR_2 = 0.1647	R_1 = 0.0581, wR_2 = 0.1334	R_1 = 0.0499. wR_2 = 0.1159	R_1 = 0.0702, wR_2 = 0.1633	R_1 = 0.0210, wR_2 = 0.0532	R_1 = 0.0416, wR_2 = 0.0867	R_1 = 0.0418, wR_2 = 0.1016
wR_2	R_1 = 0.0803, wR_2 = 0.2087	R_1 = 0.2087, wR_2 = 0.3491	R_1 = 0.1221, wR_2 = 0.1912	R_1 = 0.0832, wR_2 = 0.1427	R_1 = 0.0743, wR_2 = 0.1256	R_1 = 0.0975, wR_2 = 0.1740	R_1 = 0.0227, wR_2 = 0.0543	R_1 = 0.0678, wR_2 = 0.0948	R_1 = 0.0558, wR_2 = 0.1091
$\Delta \rho_{máx.}$, $\Delta \rho_{min.}$ (e $Å^{-3}$)	0.59/-0.40	0.94/-0.66	0.63/-0.52	0.27/-0.38	0.42/-0.43	0.84/-0.70	0.61/-0.54	0.37/-0.35	0.74/-0.81

Table S3. X-ray data collection and structure refinement parameters for compounds **10–18**.

Compound	**10**	**11**	**12**	**13**	**14**	**15**	**16**	**17**	**18**
CCDC Number	2359107	2359105	2359112	2359113	2359118	2359108	2359114	2359117	2359116
Formula	$C_{30}H_{18}F_6N_4O_2$	$C_9H_5F_3N_2O_2$	$C_9H_5F_3N_2OS$	$C_{12}H_{10}F_6N_4O_2$	$C_5H_3F_3N_2O$	$C_{11}H_7Cl_2N_2O$	$C_6H_5Cl_3N_2O$	$C_5H_3Cl_3N_2O$	$C_6H_5Cl_3N_2O$
Formula weight	580.48	230.15	246.21	356.24	164.09	289.54	227.47	213.44	227.47
Temperature (K)	100.0	100.00	100.00	100.0	100.0	99.99	100.01	100.00	100.01
Crystal system	Triclinic	Triclinic	Triclinic	Monoclinic	Triclinic	Triclinic	Monoclinic	Triclinic	Monoclinic
Space group	P-1	P-1	P-1	C2/c	P-1	P-1	$P2_1/c$	P-1	$P2_1/c$
a (Å)	9.6918(6)	5.0081(2)	4.9123(2)	14.8320(6)	6.0119(6)	7.5843(4)	15.338(9)	6.2116(3)	9.4856(5)
b (Å)	9.7079(6)	7.0337(4)	7.4145(3)	15.7643(6)	8.9108(11)	8.5237(4)	5.818(3)	7.2494(3)	8.3246(4)
c (Å)	13.4178(9)	12.6787(6)	13.2297(5)	12.2697(4)	11.4058(13)	10.2223(10)	10.320(5)	8.8062(4)	12.0956(6)
α (°)	93.649(2)	91.813(2)	88.2100(10)	90	85.845(4)	70.1600(10)	90	85.3060(10)	90
β (°)	99.543(2)	92.320(2)	83.8640(10)	105.4530(10)	78.277(4)	69.6830(10)	108.796(17)	87.6110(10)	109.3740(10)
γ (°)	98.134(2)	105.040(2)	73.8950(10)	90	84.837(4)	76.2980(10)	90	76.6010(10)	90
Cell volume ($Å^3$)	1227.53(14)	430.55(4)	460.29(3)	2765.14(18)	594.93(12)	377.61(5)	871.9(8)	384.35(3)	901.03(8)
Z	2	2	2	8	4	2	4	2	4
Calculated density ($g\ cm^{-3}$)	1.570	1.775	1.776	1.711	1.832	1.665	1.733	1.844	1.677
μ (mm^{-1})	0.132	0.170	0.376	0.173	0.192	0.774	0.999	1.127	0.967
F(000)	592.0	232.0	248.0	1440.0	328.0	292	456.0	212.0	456.0
Crystal size (mm^3)	0.806×0.29×0.216	0.433×0.125×0.083	0.541×0.23×0.12	0.644×0.101×0.089	0.605×0.215×0.082	0.573×0.072×0.069	0.239×0.222×0.124	0.171×0.152×0.115	0.609×0.188×0.115
Wavelength (Å)	Mo Kα (λ = 0.71073)	Mo Kα (λ = 0.71073)	Mo Kα (λ = 0.71073)	Mo Kα (λ = 0.71073)	Mo Kα (λ = 0.71073)	Mo Kα (λ = 0.71073)	Mo Kα (λ = 0.71073)	Mo Kα (λ = 0.71073)	Mo Kα (λ = 0.71073)
θ range for data collection (°)	4.256 to 56.624	6.004 to 56.594	5.718 to 56.62	3.846 to 56.628	4.596 to 56.746	4.43 to 56.67	5.612 to 53.012	4.642 a 56.604	4.552 a 56.618

Index ranges	-11 ≤ h ≤ 12, -12 ≤ k ≤ 12, -17 ≤ l ≤ 17	-6 ≤ h ≤ 5, -9 ≤ k ≤ 9, -16 ≤ l ≤ 16	-6 ≤ h ≤ 5, -9 ≤ k ≤ 9, -17 ≤ l ≤ 17	-19 ≤ h ≤ 19, -20 ≤ k ≤ 20, -14 ≤ l ≤ 16	-7 ≤ h ≤ 8, -11 ≤ k ≤ 11, -15 ≤ l ≤ 15	-10 ≤ h ≤ 10, -11 ≤ k ≤ 11, -13 ≤ l ≤ 13	-18 ≤ h ≤ 19, -7 ≤ k ≤ 6, -12 ≤ l ≤ 12	-8 ≤ h ≤ 8, -9 ≤ k ≤ 9, -11 ≤ l ≤ 11	-12 ≤ h ≤ 12, -10 ≤ k ≤ 11, -13 ≤ l ≤ 16
Collected reflections	59803	15103	14518	32748	17488	26608	9816	12113	27217
Independent reflections	6085 [R_{int} = 0.0329, R_{sigma} = 0.0160]	2139 [R_{int} = 0.0294, R_{sigma} = 0.0155]	2281 [R_{int} = 0.0237, R_{sigma} = 0.0153]	3454 [R_{int} = 0.0492, R_{sigma} = 0.0238]	2892 [R_{int} = 0.0493, R_{sigma} = 0.0317]	2868 [R_{int} = 0.0224, R_{sigma} = 0.0108]	1749 [R_{int} = 0.1412, R_{sigma} = 0.0955]	1904 [R_{int} = 0.0194, R_{sigma} = 0.0119	2233 [R_{int} = 0.0357, R_{sigma} = 0.0163]
Data/restraints/parameters	6085/0/387	2139/0/149	2281/0/161	3454/0/227	2892/0/223	2868/0/158	1749/0/129	1904/0/104	2233/0/114
Goodness-of-fit (F^2)	1.065	1.046	1.076	1.043	1.182	1.043	1.109	1.114	1.176
R_1 (I > 2σ(I))	R_1 = 0.0381, wR_2 = 0.1114	R_1 = 0.0336, wR_2 = 0.0894	R_1 = 0.0509, wR_2 = 0.1506	R_1 = 0.0355, wR_2 = 0.0815	R_1 = 0.1035, wR_2 = 0.2884	R_1 = 0.0602, wR_2 = 0.1613	R_1 = 0.0939, wR_2 = 0.2442	R_1 = 0.0189, wR_2 = 0.0485	R_1 = 0.0260, wR_2 = 0.0820
wR_2	R_1 = 0.0470, wR_2 = 0.1171	R_1 = 0.0403, wR_2 = 0.0932	R_1 = 0.0550, wR_2 = 0.1552	R_1 = 0.0499, wR_2 = 0.0870	R_1 = 0.1187, wR_2 = 0.2942	R_1 = 0.0632, wR_2 = 0.1637	R_1 = 0.1406, wR_2 = 0.2724	R_1 = 0.0202, wR_2 = 0.0491	R_1 = 0.0280, wR_2 = 0.0832
$\Delta\rho_{máx.}$, $\Delta\rho_{min.}$ (e $Å^{-3}$)	0.46/-0.32	0.44/-0.33	1.40/-0.76	0.40/-0.33	0.92/-0.71	1.83/-0.80	1.08/-0.67	0.39/-0.26	0.36/-0.45

3. Conformational isomorphs

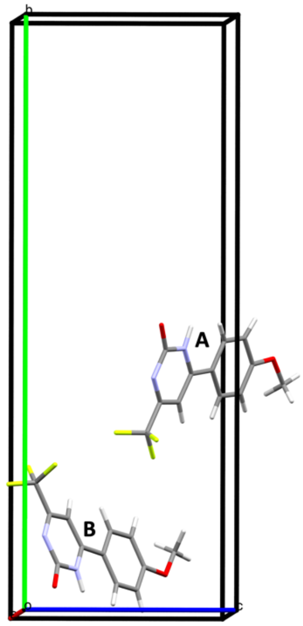

Figure S1 – Independent conformational isomorphs of compound **5** (**5A** and **5B**).

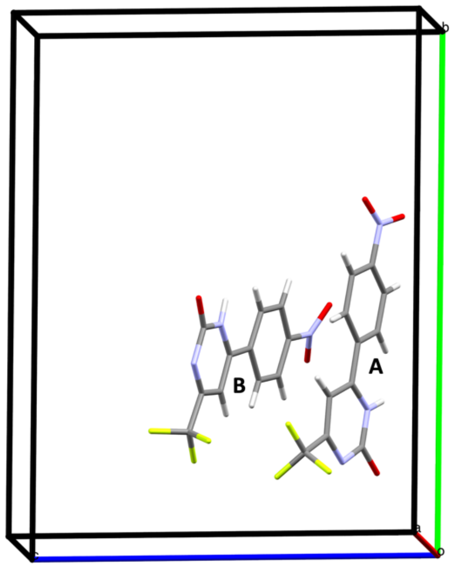

Figure S2 – Independent conformational isomorphs of compound **9** (**9A** and **9B**).

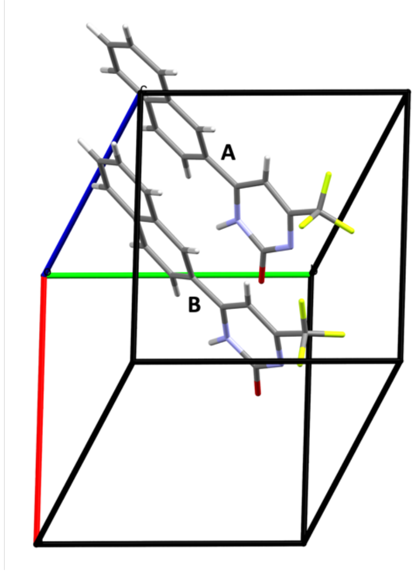

Figure S3 – Independent conformational isomorphs of compound **10** (**10A** and **10B**).

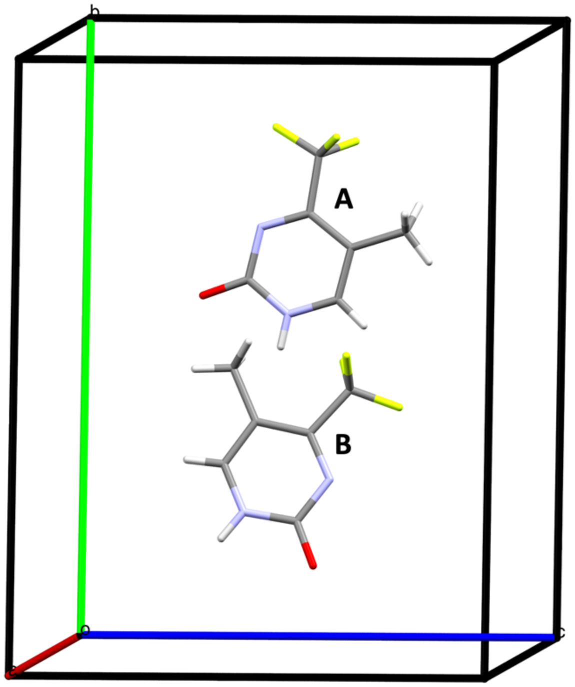

Figure S4 – Independent conformational isomorphs of compound **13** (**13A** and **13B**).

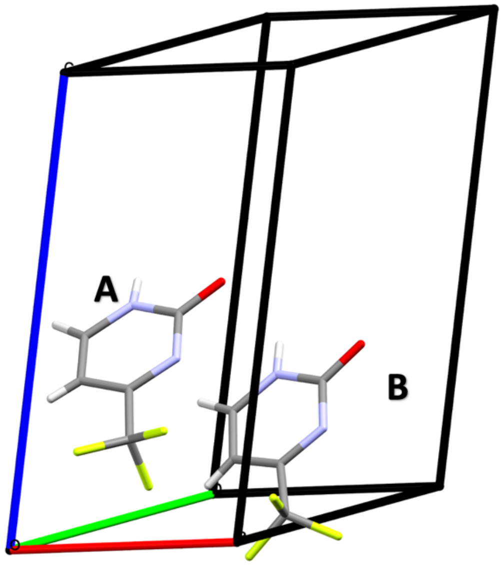

Figure S5 – Independent conformational isomorphs of compound **14** (**14A** and **14B**).

4. Supramolecular Cluster Data

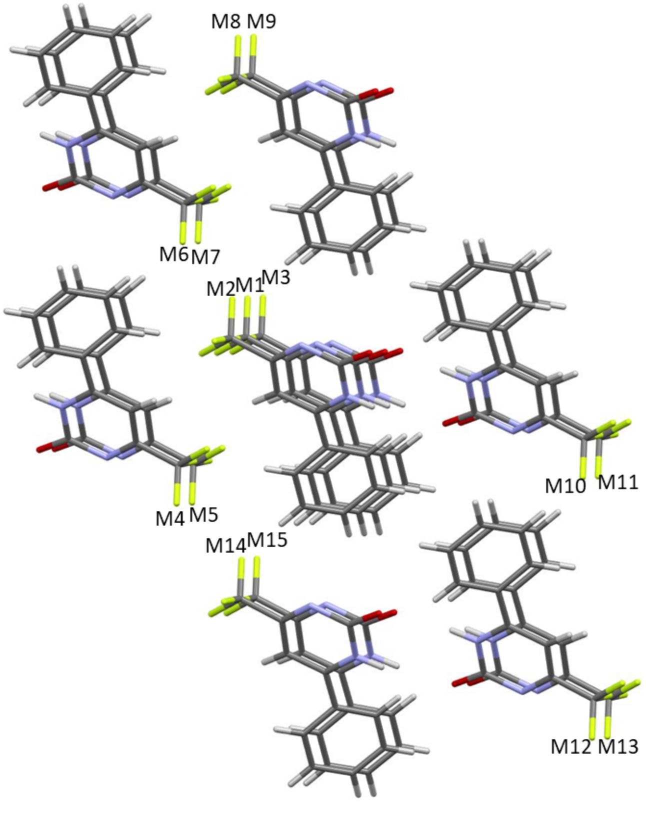

Monomer	Symmetry code	Monomer	Symmetry code
M1	x,y,z	M9	1+x,1+y,z
M2	1+x,y,z	M10	2-x,1-y,-z
M3	-1+x,y,z	M11	1-x,1-y,-z
M4	2-x,1-y,1-z	M12	-x,-y,-z
M5	1-x,1-y,1-z	M13	-1-x,-y,-z
M6	3-x,2-y,1-z	M14	-1+x,-1+y,z
M7	2-x,2-y,1-z	M15	-2+x,-1+y,z
M8	2+x,1+y,z		

Figure S6. Supramolecular cluster and symmetry codes for the monomers of compound **1**.

Table S4. Contact area ($C_{M1\cdots MN}$), stabilization energy ($G_{M1\cdots MN}$), normalized contact area and stabilization energy data ($NC_{M1\cdots MN}$ and $NG_{M1\cdots MN}$) between the dimers of the supramolecular cluster for **1**.

Dimer	$C_{M1\cdots MN}$ (Å^2)	$G_{M1\cdots MN}$ (kcal mol^{-1})	$NC_{M1\cdots MN}$	$NG_{M1\cdots MN}$
M1···M2	41.88	-8.13	2.16	1.61
M1···M3	41.88	-8.13	2.16	1.61
M1···M4	25.29	-5.25	1.30	1.04
M1···M5	24.85	-4.21	1.28	0.83
M1···M6	7.66	-0.53	0.40	0.11
M1···M7	2.14	-0.75	0.11	0.15
M1···M8	18.23	-5.26	0.94	1.04
M1···M9	13.06	-1.95	0.67	0.39
M1···M10	18.9	-21.72	1.97	4.31
M1···M11	18.17	-6.00	0.94	1.19
M1···M12	16.26	-1.61	0.84	0.32
M1···M13	11.85	0.13	0.61	-0.03
M1···M14	13.06	-1.95	0.67	0.39
M1···M15	18.23	-5.26	0.94	1.04
Total	271.46	-70.62	14.00	14.00

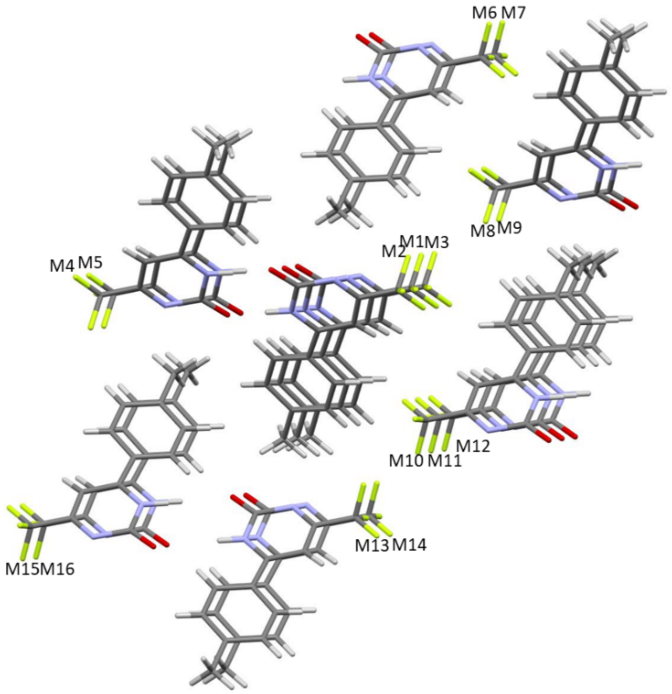

Monomer	Symmetry code	Monomer	Symmetry code
M1	x,y,z	M9	1-x,1-y,-z
M2	x,1+y,z	M10	1-x,1+y,1/2-z
M3	x,-1+y,z	M11	1-x,y,1/2-z
M4	-x,1-y,-z	M12	1-x,-1+y,1/2-z
M5	1/2-x,1/2-y,-z	M13	x,1-y,1/2+z
M6	x,1-y,-1/2+z	M14	x,-y,1/2+z
M7	x,-y,-1/2+z	M15	1/2-x,1/2+y,1/2-z
M8	1-x,2-y,-z	M16	1/2-x,-1/2+y,1/2-z

Figure S7. Supramolecular cluster and symmetry codes for the monomers of compound **2**.

Table S5. Contact area ($C_{M1\cdots MN}$), stabilization energy ($G_{M1\cdots MN}$), normalized contact area and stabilization energy data ($NC_{M1\cdots MN}$ and $NG_{M1\cdots MN}$) between the dimers of the supramolecular cluster for **2**.

Dimer	$C_{M1\cdots MN}$ (Å^2)	$G_{M1\cdots MN}$ (kcal mol^{-1})	$NC_{M1\cdots MN}$	$NG_{M1\cdots MN}$
M1···M2	48.26	-10.54	2.47	2.07
M1···M3	48.26	-10.54	2.47	2.07
M1···M4	18.23	-22.25	0.93	4.38

M1···M5	15.53	-5.30	0.80	1.04
M1···M6	20.61	-5.68	1.06	1.12
M1···M7	17.58	-3.44	0.90	0.68
M1···M8	3.48	0.04	0.18	-0.01
M1···M9	5.29	-0.37	0.27	0.07
M1···M10	14.64	-2.30	0.75	0.45
M1···M11	21.5	-3.41	1.10	0.67
M1···M12	14.64	-2.30	0.75	0.45
M1···M13	20.61	-5.68	1.06	1.12
M1···M14	17.58	-3.44	0.90	0.68
M1···M15	13.27	-0.53	0.68	0.10
M1···M16	13.27	-0.53	0.68	0.10
Total	292.75	-76.28	15.00	15.00

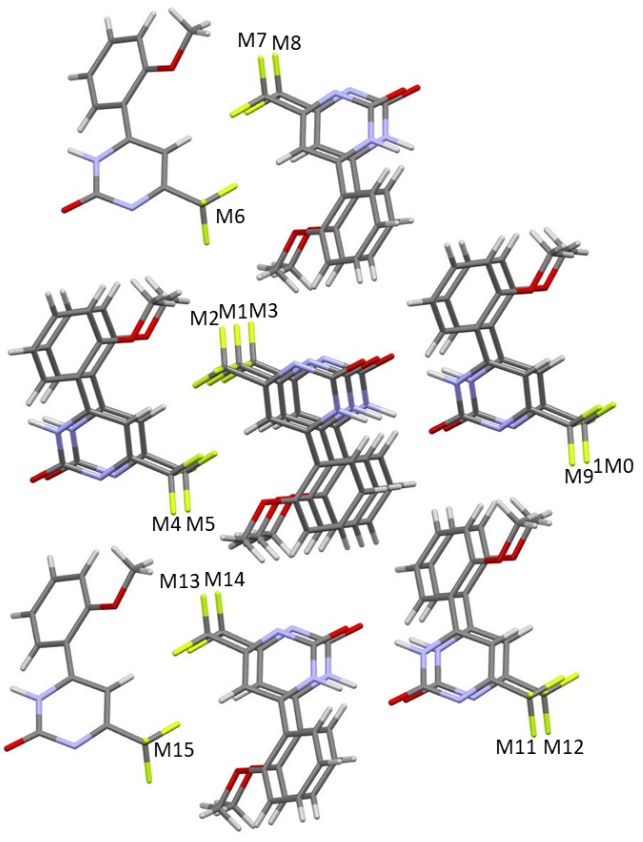

Monomer	Symmetry code	Monomer	Symmetry code
M1	x,y,z	M9	1/2-x,1/2-y,-z
M2	x,1+y,z	M10	1/2-x,-1/2-y,-z
M3	x,-1+y,z	M11	1/2-x,1/2+y,1/2-z
M4	-x,1-y,-z	M12	1/2-x,-1/2+y,1/2-z
M5	-x,-y,-z	M13	x,1-y,1/2+z
M6	-x,y,-1/2-z	M14	x,-y,1/2+z
M7	x,1-y,-1/2+z	M15	-x,y,1/2-z
M8	x,-y,-1/2+z		

Figure S8. Supramolecular cluster and symmetry codes for the monomers of compound **3**.

Table S6. Contact area ($C_{M1\cdots MN}$), stabilization energy ($G_{M1\cdots MN}$), normalized contact area and stabilization energy data ($NC_{M1\cdots MN}$ and $NG_{M1\cdots MN}$) between the dimers of the supramolecular cluster for **3**.

Dimer	$C_{M1\cdots MN}$ (Å^2)	$G_{M1\cdots MN}$ (kcal mol^{-1})	$NC_{M1\cdots MN}$	$NG_{M1\cdots MN}$
M1···M2	49.11	-5.93	2.28	1.08
M1···M3	49.11	-5.93	2.28	1.08
M1···M4	37.72	-5.84	1.75	1.06
M1···M5	27.48	-5.66	1.28	1.03
M1···M6	2.04	0.78	0.09	-0.14
M1···M7	12.23	-4.34	0.57	0.79
M1···M8	20.35	-6.31	0.95	1.15
M1···M9	20.04	-23.00	0.93	4.17
M1···M10	13.62	-5.94	0.63	1.08
M1···M11	16.14	-2.36	0.75	0.43
M1···M12	16.14	-2.36	0.75	0.43
M1···M13	12.23	-4.34	0.57	0.79
M1···M14	20.35	-6,31	0.95	1.15
M1···M15	4.74	0.40	0.22	-0.07
Total	301.3	-77.14	14.00	14.00

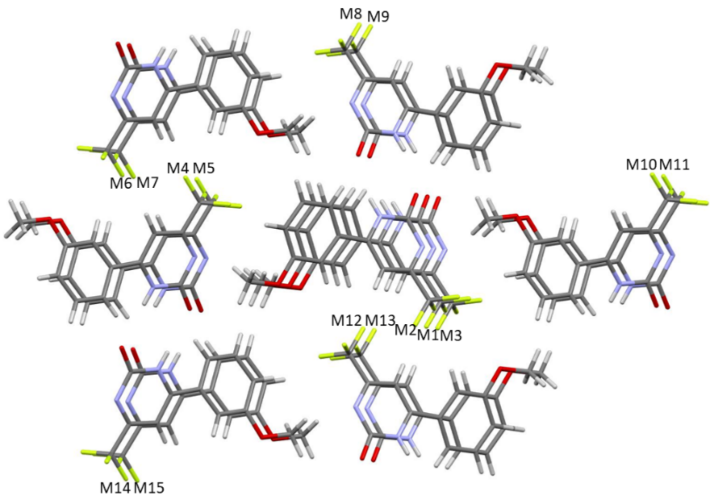

Monomer	Symmetry code	Monomer	Symmetry code
M1	x,y,z	M9	-1-x,1-y,-z
M2	1+x,y,z	M10	-1/2+x,1/2-y,-1/2+z
M3	-1+x,y,z	M11	-1.5+x,1/2-y,-1/2+z
M4	1.5+x,1/2-y,1/2+z	M12	-x,-y,-z
M5	1/2+x,1/2-y,1/2+z	M13	-1-x,-y,-z
M6	1.5-x,1/2+y,1/2-z	M14	1.5-x,-1/2+y,1/2-z
M7	1/2-x,1/2+y,1/2-z	M15	1/2-x,-1/2+y,1/2-z
M8	-x,1-y,-z		

Figure S9. Supramolecular cluster and symmetry codes for the monomers of compound **4**.

Table S7. Contact area ($C_{M1\cdots MN}$), stabilization energy ($G_{M1\cdots MN}$), normalized contact area and stabilization energy data ($NC_{M1\cdots MN}$ and $NG_{M1\cdots MN}$) between the dimers of the supramolecular cluster for **4**.

Dimer	$C_{M1\cdots MN}$ (Å^2)	$G_{M1\cdots MN}$ (kcal mol^{-1})	$NC_{M1\cdots MN}$	$NG_{M1\cdots MN}$
M1···M2	50.07	-10.11	2.33	1.92
M1···M3	50.07	-10.11	2.33	1.92
M1···M4	19.29	-6.46	0.90	1.23
M1···M5	16.15	-1.75	0.75	0.33
M1···M6	8.21	0.14	0.38	-0.33
M1···M7	11.93	-1.17	0.55	0.22

M1···M8	15.42	-6.34	0.72	1.20
M1···M9	17.05	-19.76	0.79	3.75
M1···M10	16.15	-1.75	0.75	0.33
M1···M11	19.29	-6.46	0.90	1.23
M1···M12	31.86	-4.51	1.48	0.86
M1···M13	25.43	-4.41	1.18	0.84
M1···M14	8.21	0.14	0.38	-0.03
M1···M15	11.93	-1.17	0.55	0.22
Total	301.06	-73.72	14.00	14.00

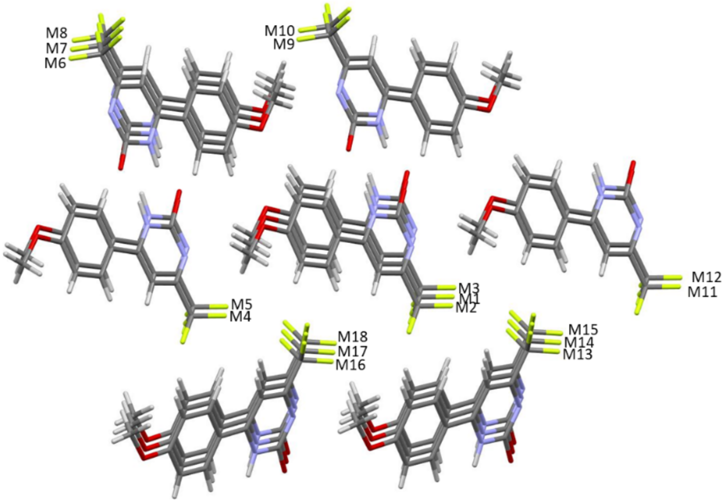

Monomer	Symmetry code	Monomer	Symmetry code
M1	x,y,z	M10	-x,1/2+y,1-z
M2	1+x,y,z	M11	-1+x,y,-1+z
M3	-1+x,y,z	M12	-2+x,y,-1+z
M4	2+x,y,1+z	M13	x,y,z
M5	1+x,y,1+z	M14	-1+x,y,z
M6	3-x,1/2+y,2-z	M15	-2+x,y,z
M7	2-x,1/2+y,2-z	M16	1+x,y,1+z
M8	1-x,1/2+y,2-z	M17	x,y,1+z
M9	1-x,1/2+y,1-z	M18	-1+x,y,1+z

Figure S10. Supramolecular cluster and symmetry codes for the monomers of compound **5A**.

Table S8. Contact area ($C_{M1\cdots MN}$), stabilization energy ($G_{M1\cdots MN}$), normalized contact area and stabilization energy data ($NC_{M1\cdots MN}$ and $NG_{M1\cdots MN}$) between the dimers of the supramolecular cluster for **5A**.

Dimer	$C_{M1\cdots MN}$ (Å^2)	$G_{M1\cdots MN}$ (kcal mol^{-1})	$NC_{M1\cdots MN}$	$NG_{M1\cdots MN}$
M1···M2	57.06	-11.80	3.15	2.59
M1···M3	57.06	-11.80	3.15	2.59
M1···M4	15.45	-5.05	0.85	1.11
M1···M5	16.83	-3.22	0.93	0.71
M1···M6	1.19	-0.01	0.07	0.00
M1···M7	24.8	-2.60	1.37	0.57
M1···M8	0.49	-1.49	0.03	0.33
M1···M9	19.14	-4.46	1.06	0.98
M1···M10	21.8	-23.89	1.20	5.25
M1···M11	16.83	-3.22	0.93	0.71
M1···M12	15.45	-5.05	0.85	1.11
M1···M13	0.92	0.73	0.05	-0.16
M1···M14	17.23	-0.82	0.95	0.18
M1···M15	14.9	-2.16	0.82	0.47
M1···M16	7.34	-1.65	0.40	0.36
M1···M17	19.16	-1.35	1.06	0.30
M1···M18	2.51	0.45	0.14	-0.10
Total	308.16	-77.39	17.00	17.00

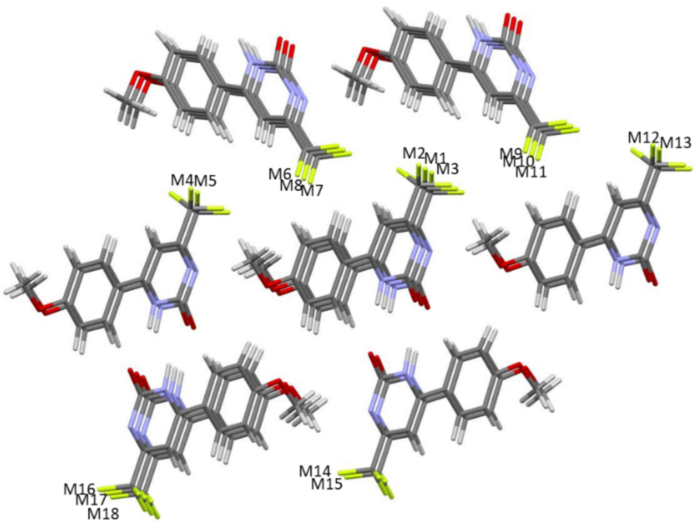

Monomer	Symmetry code	Monomer	Symmetry code
M1	x,y,z	M10	x,y,-1+z
M2	1+x,y,z	M11	-1+x,y,-1+z
M3	-1+x,y,z	M12	-1+x,y,-1+z
M4	2+x,y,1+z	M13	-2+x,y,-1+z
M5	1+x,y,1+z	M14	-x,-1/2+y,1-z
M6	2+x,y,z	M15	-1-x,-1/2+y,1-z
M7	1+x,y,z	M16	3-x,-1/2+y,2-z
M8	x,y,z	M17	2-x,-1/2+y,2-z
M9	1+x,y,-1+z	M18	1-x,-1/2+y,2-z

Figure S11. Supramolecular cluster and symmetry codes for the monomers of compound **5B**.

Table S9. Contact area ($C_{M1\cdots MN}$), stabilization energy ($G_{M1\cdots MN}$), normalized contact area and stabilization energy data ($NC_{M1\cdots MN}$ and $NG_{M1\cdots MN}$) between the dimers of the supramolecular cluster for **5B**.

Dimer	$C_{M1\cdots MN}$ (Å^2)	$G_{M1\cdots MN}$ (kcal mol^{-1})	$NC_{M1\cdots MN}$	$NG_{M1\cdots MN}$
M1···M2	56.09	-12.08	3.05	2.82
M1···M3	56.09	-12.08	3.05	2.82
M1···M4	15.98	-5.04	0.87	1.18
M1···M5	19.31	-2.98	1.05	0.70
M1···M6	14.90	-2.16	0.81	0.50
M1···M7	17.23	-0.82	0.94	0.19
M1···M8	0.92	0.73	0.05	-0.17
M1···M9	2.51	0.45	0.14	-0.11
M1···M10	19.16	-1.35	1.04	0.31
M1···M11	7.34	-1.65	0.40	0.39
M1···M12	19.31	-2.98	1.05	0.70
M1···M13	15.98	-5.04	0.87	1.18
M1···M14	19.14	-23.89	1.04	5.57
M1···M15	21.8	0.10	1.19	-0.02
M1···M16	1.19	-0.01	0.06	0.00
M1···M17	24.8	-2.60	1.35	0.61
M1···M18	0.49	-1.49	0.03	0.35

Total	312.24	-72.88	17.00	17.00

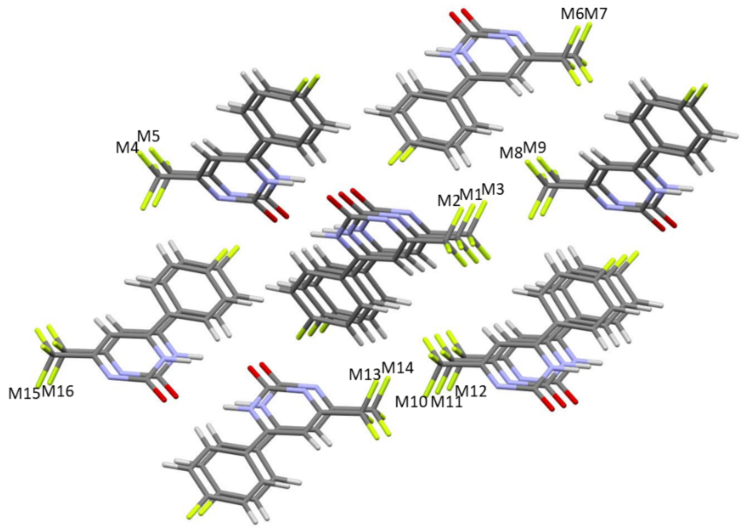

Monomer	Symmetry code	Monomer	Symmetry code
M1	x,y,z	M9	1-x,1-y,-z
M2	x,1+y,z	M10	1-x,1+y,1/2-z
M3	x,-1+y,z	M11	1-x,y,1/2-z
M4	1/2-x,1.5-y,-z	M12	1-x,-1+y,1/2-z
M5	1/2-x,1/2-y,-z	M13	x,1-y,1/2+z
M6	x,1-y,-1/2+z	M14	x,-y,1/2+z
M7	x,-y,-1/2+z	M15	1/2-x,1/2+y,1/2-z
M8	1-x,2-y,-z	M16	1/2-x,-1/2+y,1/2-z

Figure S12. Supramolecular cluster and symmetry codes for the monomers of compound **6**.

Table S10. Contact area ($C_{M1\cdots MN}$), stabilization energy ($G_{M1\cdots MN}$), normalized contact area and stabilization energy data ($NC_{M1\cdots MN}$ and $NG_{M1\cdots MN}$) between the dimers of the supramolecular cluster for **6**.

Dimer	$C_{M1\cdots MN}$ (Å^2)	$G_{M1\cdots MN}$ (kcal mol^{-1})	$NC_{M1\cdots MN}$	$NG_{M1\cdots MN}$
M1···M2	39.15	-9.80	2.21	1.97
M1···M3	39.15	-9.80	2.21	1.97
M1···M4	19.09	-25.30	1.08	5.08
M1···M5	15.70	-4.84	0.89	0.97
M1···M6	19.92	-4.63	1.12	0.93

M1···M7	15.55	-2.06	0.88	0.41
M1···M8	4.36	-0.37	0.25	0.07
M1···M9	6.82	-0.78	0.38	0.16
M1···M10	12.36	-2.47	0.70	0.50
M1···M11	19.09	-2.76	1.08	0.55
M1···M12	12.36	-2.47	0.70	0.50
M1···M13	19.92	-4.63	1.12	0.93
M1···M14	15.55	-2.06	0.88	0.41
M1···M15	13.49	-1.38	0.76	0.28
M1···M16	13.49	-1.38	0.76	0.28
Total	266	-74.75	15.00	15.00

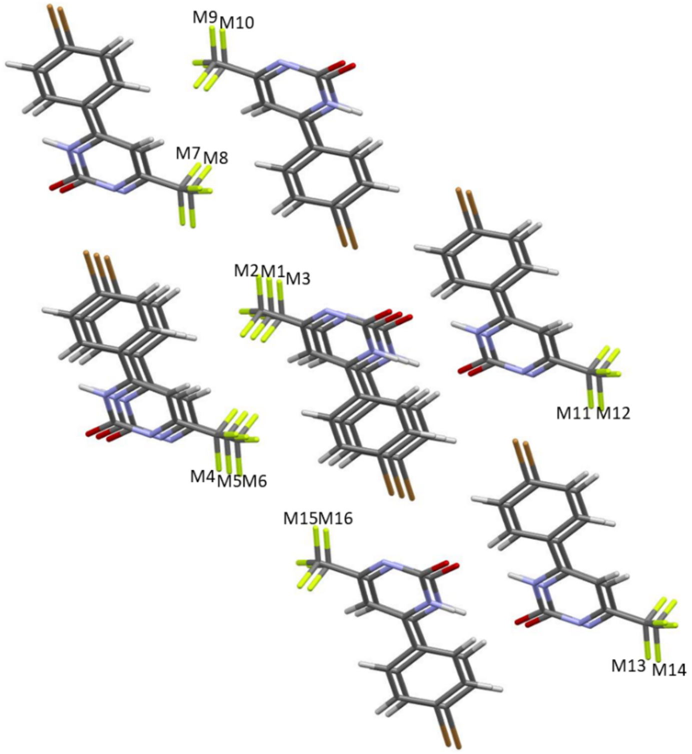

Monomer	Symmetry code	Monomer	Symmetry code
M1	x,y,z	M9	2+x,1+y,z
M2	1+x,y,z	M10	1+x,1+y,z
M3	-1+x,y,z	M11	2-x,1-y,-z
M4	3-x,1-y,1-z	M12	1-x,1-y,-z
M5	2-x,1-y,1-z	M13	-x,-y,-z

M6	1-x,1-y,1-z	M14	-1-x,-y,-z
M7	3-x,2-y,1-z	M15	-1+x,-1+y,z
M8	2-x,2-y,1-z	M16	-2+x,-1+y,z

Figure S13. Supramolecular cluster and symmetry codes for the monomers of compound **7**.

Table S11. Contact area ($C_{M1\cdots MN}$), stabilization energy ($G_{M1\cdots MN}$), normalized contact area and stabilization energy data ($NC_{M1\cdots MN}$ and $NG_{M1\cdots MN}$) between the dimers of the supramolecular cluster for **7**.

Dimer	$C_{M1\cdots MN}$ (Å^2)	$G_{M1\cdots MN}$ (kcal mol^{-1})	$NC_{M1\cdots MN}$	$NG_{M1\cdots MN}$
M1···M2	51.11	-12.10	2.64	2.22
M1···M3	51.11	-12.10	2.64	2.22
M1···M4	0.38	-1.05	0.02	0.19
M1···M5	24.43	-5.85	1.26	1.07
M1···M6	24.46	-3.52	1.26	0.64
M1···M7	4.5	-0.19	0.23	0.03
M1···M8	1.74	-0.71	0.09	0.13
M1···M9	13.79	-3.21	0.71	0.59
M1···M10	19.38	-4.00	1.00	0.73
M1···M11	19.43	-23.69	1.00	4.34
M1···M12	21.37	-4.86	1.10	0.89
M1···M13	20.59	-2.71	1.06	0.50
M1···M14	5	-0.71	0.26	0.13
M1···M15	19.38	-4.00	1.00	0.73
M1···M16	13.79	-3.21	0.71	0.59
Total	290.46	-81.92	15.00	15.00

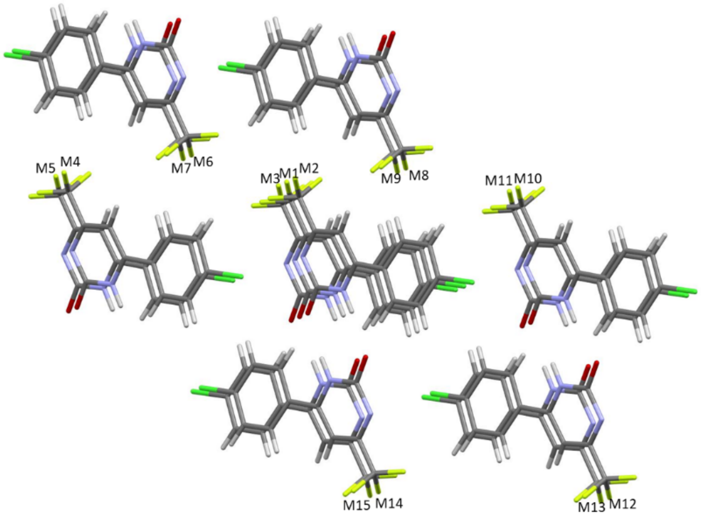

Monomer	Symmetry code	Monomer	Symmetry code
M1	x,y,z	M9	3-x,1-y,1-z
M2	-1+x,y,z	M10	-2+x,-1+y,z
M3	1+x,y,z	M11	-1+x,-1+y,z
M4	1+x,1+y,z	M12	-1-x,-y,-z
M5	2+x,1+y,z	M13	-x,-y,-z
M6	2-x,2-y,1-z	M14	1-x,1-y,-z
M7	3-x,2-y,1-z	M15	2-x,1-y,-z
M8	2-x,1-y,1-z		

Figure S14. Supramolecular cluster and symmetry codes for the monomers of compound **8**.

Table S12. Contact area ($C_{M1\cdots MN}$), stabilization energy ($G_{M1\cdots MN}$), normalized contact area and stabilization energy data ($NC_{M1\cdots MN}$ and $NG_{M1\cdots MN}$) between the dimers of the supramolecular cluster for **8**.

Dimer	$C_{M1\cdots MN}$ (Å^2)	$G_{M1\cdots MN}$ (kcal mol^{-1})	$NC_{M1\cdots MN}$	$NG_{M1\cdots MN}$
M1···M2	51.18	-11.27	2.53	2.13
M1···M3	51.18	-11.27	2.53	2.13
M1···M4	18.44	-3.84	0.91	0.73
M1···M5	12.98	-2.09	0.64	0.40
M1···M6	3.47	-0.82	0.17	0.15
M1···M7	4.57	-0.23	0.23	0.04
M1···M8	23.09	-5.60	1.14	1.06

M1···M9	23.41	-1.04	1.15	0.20
M1···M10	12.98	-2.09	0.64	0.40
M1···M11	18.44	-3.84	0.91	0.73
M1···M12	4.56	-0.58	0.22	0.11
M1···M13	20.10	-2.21	0.99	0.42
M1···M14	20.33	-4.96	1.00	0.94
M1···M15	19.04	-24.08	0.94	4.56
Total	283.77	-73.92	14.00	14.00

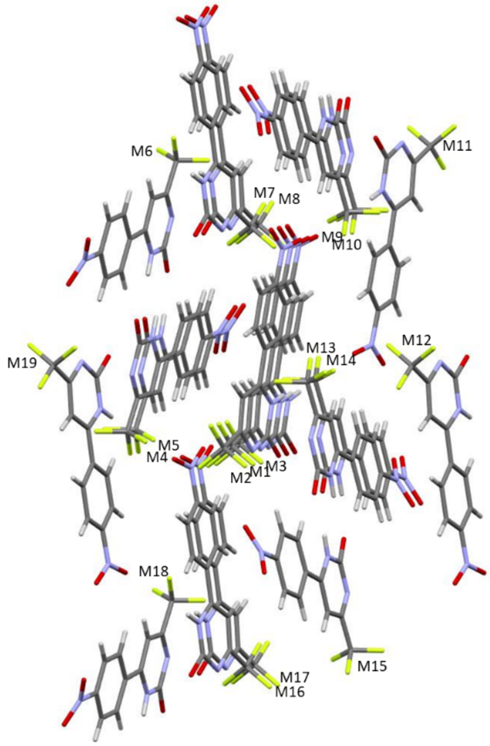

Monomer	Symmetry code	Monomer	Symmetry code
M1	x,y,z	M11	1-x,1-y,-z
M2	1+x,y,z	M12	-1+x,1/2-y,-1/2+z
M3	-1+x,y,z	M13	x,1/2-y,-1/2+z
M4	1+x,y,z	M14	-1+x,1/2-y,-1/2+z
M5	x,y,z	M15	1-x,-1/2+y,1/2-z
M6	2-x,1-y,1-z	M16	2-x,-1/2+y,1/2-z
M7	2-x,1/2+y,1/2-z	M17	1-x,-1/2+y,1/2-z
M8	1-x,1/2+y,1/2-z	M18	2-x,-y,1-z
M9	2-x,1/2+y,1/2-z	M19	1+x,1/2-y,1/2+z

M10	1-x,1/2+y,1/2-z

Figure S15. Supramolecular cluster and symmetry codes for the monomers of compound **9A**.

Table S13. Contact area ($C_{M1\cdots MN}$), stabilization energy ($G_{M1\cdots MN}$), normalized contact area and stabilization energy data ($NC_{M1\cdots MN}$ and $NG_{M1\cdots MN}$) between the dimers of the supramolecular cluster for **9A**.

Dimer	$C_{M1\cdots MN}$ (Å^2)	$G_{M1\cdots MN}$ (kcal mol^{-1})	$NC_{M1\cdots MN}$	$NG_{M1\cdots MN}$
M1···M2	0.85	1.25	0.05	-0.40
M1···M3	0.85	1.25	0.05	-0.40
M1···M4	19.14	-5.82	1.14	1.84
M1···M5	53.13	-8.61	3.17	2.72
M1···M6	1.34	-1.60	0.08	0.51
M1···M7	13.09	-2.18	0.78	0.69
M1···M8	22.09	-6.40	1.32	2.03
M1···M9	6.24	-0.13	0.37	0.04
M1···M10	21.05	-5.40	1.26	1.71
M1···M11	29.88	-5.79	1.78	1.83
M1···M12	0.1	-1.08	0.01	0.34
M1···M13	48.12	-8.83	2.87	2.79
M1···M14	28.5	3.44	1.70	-1.09
M1···M15	21.56	-7.45	1.29	2.36
M1···M16	13.09	-2.18	0.78	0.69
M1···M17	22.09	-6.40	1.32	2.03
M1···M18	0.55	0.09	0.03	-0.03
M1···M19	0.1	-1.08	0.01	0.34
Total	301.77	-56.92	18.00	18.00

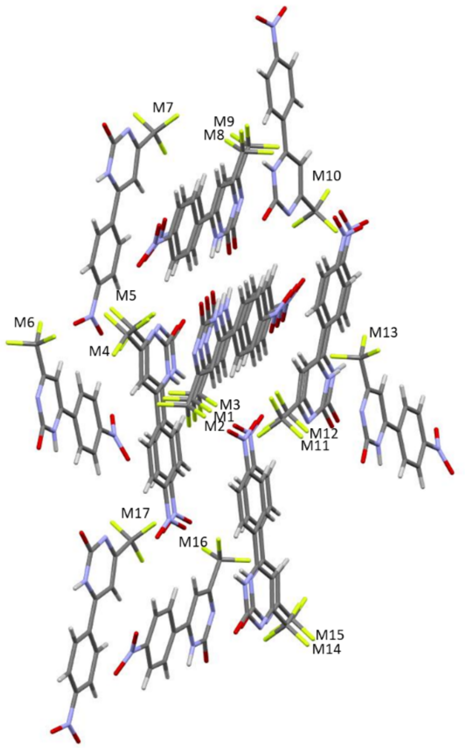

Monomer	Symmetry code	Monomer	Symmetry code
M1	x,y,z	M10	1-x,1/2+y,1/2-z
M2	1+x,y,z	M11	x,y,z
M3	-1+x,y,z	M12	-1+x,y,z
M4	1+x,1/2-y,1/2+z	M13	-1+x,1/2-y,-1/2+z
M5	x,1/2-y,1/2+z	M14	2-x,-1/2+y,1/2-z
M6	1+x,1/2-y,1/2+z	M15	1-x,-1/2+y,1/2-z
M7	2-x,1-y,1-z	M16	2-x,-y,1-z
M8	2-x,1-y,1-z	M17	2-x,-y,1-z
M9	1-x,1-y,1-z		

Figure S16. Supramolecular cluster and symmetry codes for the monomers of compound **9B**.

Table S14. Contact area ($C_{M1\cdots MN}$), stabilization energy ($G_{M1\cdots MN}$), normalized contact area and stabilization energy data ($NC_{M1\cdots MN}$ and $NG_{M1\cdots MN}$) between the dimers of the supramolecular cluster for **9B**.

Dimer	$C_{M1\cdots MN}$ (Å^2)	$G_{M1\cdots MN}$ (kcal mol^{-1})	$NC_{M1\cdots MN}$	$NG_{M1\cdots MN}$
M1···M2	19.37	-8.55	1.04	1.69
M1···M3	19.37	-8.55	1.04	1.69

M1···M4	28.5	3.44	1.53	-0.68
M1···M5	48.12	-8.83	2.58	1.75
M1···M6	9.21	-1.95	0.49	0.39
M1···M7	1.34	-1.60	0.07	0.32
M1···M8	23.14	-24.58	1.24	4.87
M1···M9	18.4	-0.38	0.99	0.08
M1···M10	21.56	-7.45	1.16	1.48
M1···M11	53.13	-8.61	2.85	1.71
M1···M12	19.14	-5.82	1.03	1.15
M1···M13	9.21	-1.95	0.49	0.39
M1···M14	6.24	-0.13	0.33	0.03
M1···M15	21.05	-5.40	1.13	1.07
M1···M16	0.26	-0.48	0.01	0.10
M1···M17	0.55	0.09	0.03	-0.02
Total	398.59	-80.77	16.00	16.00

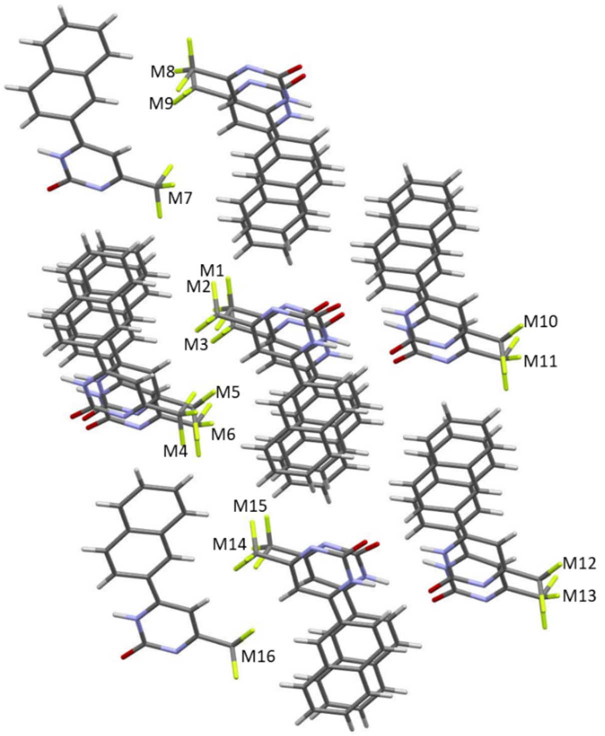

Monomer	Symmetry code	Monomer	Symmetry code
M1	x,y,z	M9	x,1+y,z

M2	x,y,z	M10	1-x,1-y,1-z
M3	-1+x,y,z	M11	-x,1-y,1-z
M4	1-x,1-y,2-z	M12	-x,-y,1-z
M5	1-x,1-y,2-z	M13	-1-x,-y,1-z
M6	-x,1-y,2-z	M14	-1+x,-1+y,z
M7	1-x,2-y,2-z	M15	-1+x,-1+y,z
M8	1+x,1+y,z	M16	-x,-y,2-z

Figure S17. Supramolecular cluster and symmetry codes for the monomers of compound **10A**.

Table S15. Contact area ($C_{M1\cdots MN}$), stabilization energy ($G_{M1\cdots MN}$), normalized contact area and stabilization energy data ($NC_{M1\cdots MN}$ and $NG_{M1\cdots MN}$) between the dimers of the supramolecular cluster for **10A**.

Dimer	$C_{M1\cdots MN}$ (Å^2)	$G_{M1\cdots MN}$ (kcal mol^{-1})	$NC_{M1\cdots MN}$	$NG_{M1\cdots MN}$
M1···M2	59.63	-13.55	2.80	2.41
M1···M3	51.05	-13.49	2.80	2.40
M1···M4	2.77	-1.49	0.13	0.25
M1···M5	35.69	-7.39	1.67	1.31
M1···M6	14.69	-2.64	0.69	0.47
M1···M7	1.12	-0.06	0.05	0.01
M1···M8	18.47	-4.38	0.87	0.78
M1···M9	17.1	-2.87	0.80	0.51
M1···M10	20.23	-23.86	0.95	4.24
M1···M11	26.97	-6.37	1.26	1.13
M1···M12	22.88	-2.59	1.07	0.46
M1···M13	12.89	0.01	0.60	0.00
M1···M14	11.48	-1.51	0.54	0.27
M1···M15	18.47	-4.38	0.87	0.78
M1···M16	6.53	0.01	0.31	0.00
Total	319.97	-84.46	15.00	15.00

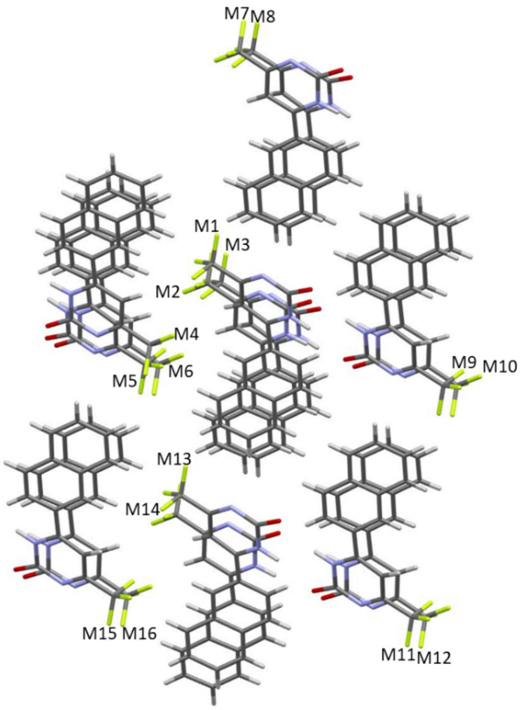

Monomer	Symmetry code	Monomer	Symmetry code
M1	x,y,z	M9	1-x,1-y,1-z
M2	1+x,y,z	M10	1-x,1-y,1-z
M3	x,y,z	M11	-x,-y,1-z
M4	2-x,1-y,2-z	M12	-x,-y,1-z
M5	1-x,1-y,2-z	M13	x,-1+y,z
M6	1-x,1-y,2-z	M14	-1+x,-1+y,z
M7	1+x,1+y,z	M15	-x,-y,2-z
M8	1+x,1+y,z	M16	-x,-y,2-z

Figure S18. Supramolecular cluster and symmetry codes for the monomers of compound **10B**.

Table S16. Contact area ($C_{M1\cdots MN}$), stabilization energy ($G_{M1\cdots MN}$), normalized contact area and stabilization energy data ($NC_{M1\cdots MN}$ and $NG_{M1\cdots MN}$) between the dimers of the supramolecular cluster for **10B**.

Dimer	$C_{M1\cdots MN}$ (Å^2)	$G_{M1\cdots MN}$ (kcal mol^{-1})	$NC_{M1\cdots MN}$	$NG_{M1\cdots MN}$
M1···M2	51.05	-13.49	2.40	2.32
M1···M3	59.63	-13.55	2.80	2.33
M1···M4	5.61	-1.83	0.26	0.31

M1···M5	35.69	-7.39	1.67	1.27
M1···M6	23.39	-4.09	1.10	0.70
M1···M7	17.79	-5.02	0.83	0.86
M1···M8	11.48	-1.51	0.54	0.26
M1···M9	20.23	-23.86	0.95	4.11
M1···M10	15.98	-6.24	0.75	1.07
M1···M11	22.88	-2.59	1.07	0.45
M1···M12	14.23	0.02	0.67	0.00
M1···M13	17.79	-2.87	0.83	0.49
M1···M14	17.1	-5.02	0.80	0.86
M1···M15	6.53	0.01	0.31	0.00
M1···M16	0.29	0.26	0.01	-0.04
Total	319.67	-87.16	15.00	15.00

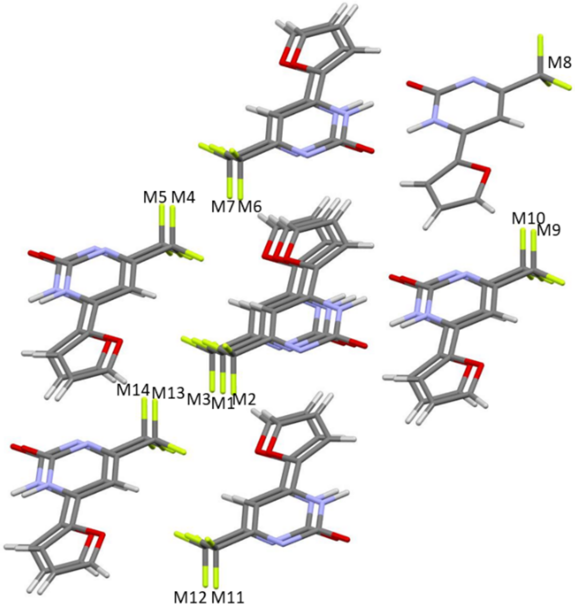

Monomer	Symmetry code	Monomer	Symmetry code
M1	x,y,z	M8	1-x,2-y,-z
M2	1+x,y,z	M9	2-x,1-y,-z
M3	-1+x,y,z	M10	1-x,1-y,-z
M4	2-x,1-y,1-z	M11	1+x,-1+y,z
M5	1-x,1-y,1-z	M12	x,-1+y,z
M6	x,1+y,z	M13	2-x,-y,1-z

M7	-1+x,1+y,z	M14	1-x,-y,1-z

Figure S19. Supramolecular cluster and symmetry codes for the monomers of compound **11**.

Table S18. Contact area ($C_{M1\cdots MN}$), stabilization energy ($G_{M1\cdots MN}$), normalized contact area and stabilization energy data ($NC_{M1\cdots MN}$ and $NG_{M1\cdots MN}$) between the dimers of the supramolecular cluster for **11**.

Dimer	$C_{M1\cdots MN}$ (Å^2)	$G_{M1\cdots MN}$ (kcal mol^{-1})	$NC_{M1\cdots MN}$	$NG_{M1\cdots MN}$
M1···M2	35.32	-6.60	1.86	1.17
M1···M3	35.32	-6.60	1.86	1.17
M1···M4	19.08	-3.76	1.01	0.66
M1···M5	16.88	-3.01	0.89	0.53
M1···M6	20.86	-4.01	1.10	0.71
M1···M7	18.55	-7.42	0.98	1.31
M1···M8	12.32	1.40	0.65	-0.25
M1···M9	22.07	-27.17	1.16	4.80
M1···M10	11.2	-4.44	0.59	0.78
M1···M11	18.55	-7.42	0.98	1.31
M1···M12	20.86	-4.01	1.10	0.71
M1···M13	11.06	0.45	0.58	-0.08
M1···M14	4.29	-1.00	0.23	0.18
Total	246.36	-73.72	13.00	13.00

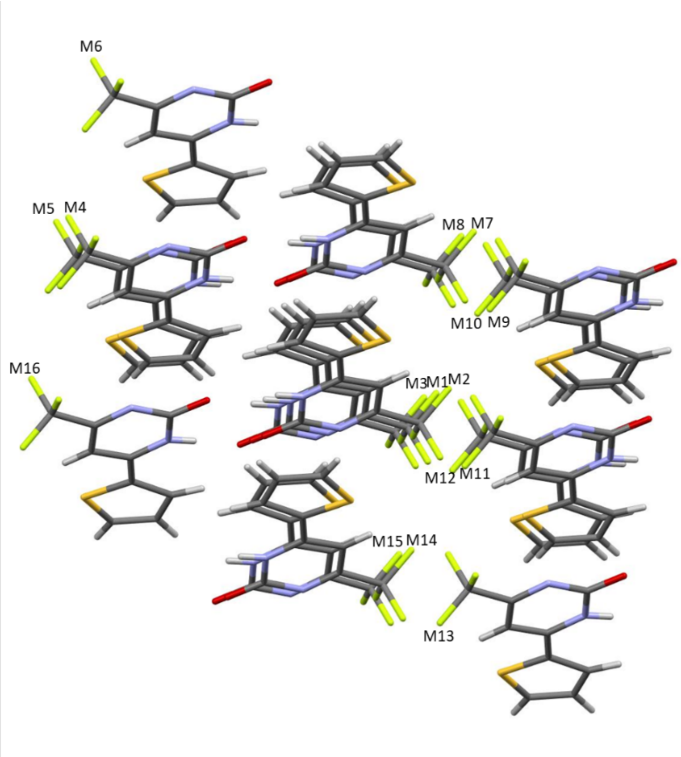

Monomer	Symmetry code	Monomer	Symmetry code
M1	x,y,z	M9	2-x,1-y,1-z
M2	x,1+y,z	M10	2-x,-y,1-z
M3	x,-1+y,z	M11	1-x,1-y,1-z
M4	2-x,2-y,-z	M12	1-x,-y,1-z
M5	2-x,1-y,-z	M13	-x,1-y,1-z
M6	3-x,2-y,-z	M14	-1+x,y,z
M7	1+x,1+y,z	M15	-1+x,-1+y,z
M8	1+x,y,z	M16	1-x,1-y,-z

Figure S20. Supramolecular cluster and symmetry codes for the monomers of compound **12**.

Table S18. Contact area ($C_{M1\cdots MN}$), stabilization energy ($G_{M1\cdots MN}$), normalized contact area and stabilization energy data ($NC_{M1\cdots MN}$ and $NG_{M1\cdots MN}$) between the dimers of the supramolecular cluster for **12**.

Dimer	$C_{M1\cdots MN}$ (Å^2)	$G_{M1\cdots MN}$ (kcal mol^{-1})	$NC_{M1\cdots MN}$	$NG_{M1\cdots MN}$
M1···M2	16.66	-3.14	0.98	0.65
M1···M3	16.66	-3.14	0.98	0.65
M1···M4	14.34	0.30	0.85	-0.06
M1···M5	19.29	-6.02	1.14	1.24

M1···M6	2.58	0.80	0.15	-0.17
M1···M7	16.41	-6.79	0.97	1.40
M1···M8	40.45	-8.50	2.39	1.75
M1···M9	8.4	-2.44	0.50	0.50
M1···M10	2.92	-0.88	1.17	0.18
M1···M11	23.09	-4.55	1.36	0.94
M1···M12	14.3	0.09	0.86	-0.02
M1···M13	2.76	-0.77	0.16	0.16
M1···M14	40.45	-8.50	2.39	1.75
M1···M15	16.41	-6.79	0.97	1.40
M1···M16	19.52	-22.50	1.15	4.63
Total	254.24	-72.81	15.00	15.00

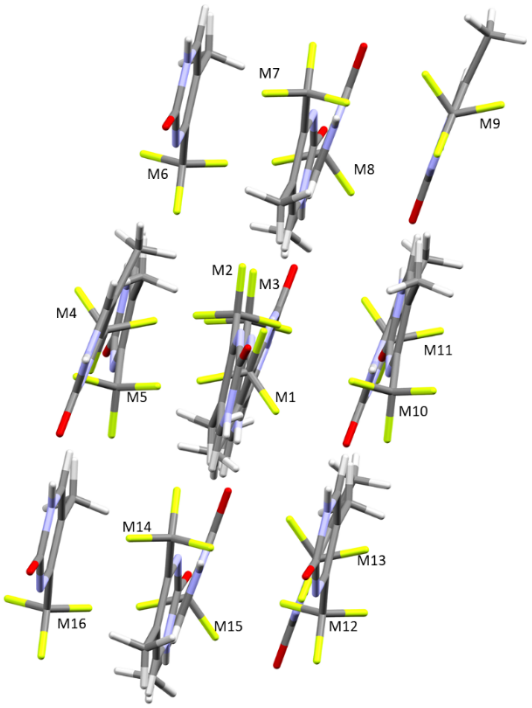

Monomer	Symmetry code	Monomer	Symmetry code
M1	x,y,z	M9	1-x,y,1/2-z
M2	1/2-x,1.5-y,1-z	M10	1/2+x,1/2+y,z
M3	1/2-x,1/2-y,1-z	M11	1-x,1-y,1-z
M4	1/2-x,1.5-y,1-z	M12	1/2+x,1.5-y,1/2+z
M5	x,y,z	M13	1-x,y,1.5-z

M6	x,1-y,-1/2+z	M14	1/2-x,1/2+y,1.5-z
M7	1/2-x,1/2+y,1/2-z	M15	x,1-y,1/2+z
M8	x,1-y,-1/2+z	M16	x,1-y,1/2+z

Figure S21. Supramolecular cluster and symmetry codes for the monomers of compound **13A**.

Table S19. Contact area ($C_{M1\cdots MN}$), stabilization energy ($G_{M1\cdots MN}$), normalized contact area and stabilization energy data ($NC_{M1\cdots MN}$ and $NG_{M1\cdots MN}$) between the dimers of the supramolecular cluster for **13A**.

Dimer	$C_{M1\cdots MN}$ (Å^2)	$G_{M1\cdots MN}$ (kcal mol^{-1})	$NC_{M1\cdots MN}$	$NG_{M1\cdots MN}$
M1···M2	14.44	-0.61	1.11	0.16
M1···M3	8.26	-5.29	0.63	1.40
M1···M4	28.71	-5.69	2.20	1.50
M1···M5	13.27	-2.78	1.02	0.73
M1···M6	4.13	-0.34	0.32	0.09
M1···M7	20.01	-10.83	1.53	2.86
M1···M8	8.26	-4.89	0.63	1.29
M1···M9	1.14	2.60	0.09	-0.69
M1···M10	22.82	-9.83	1.75	2.59
M1···M11	11.96	-6.67	0.92	1.76
M1···M12	0.28	-0.69	0.02	0.18
M1···M13	25.52	-1.96	1.95	0.52
M1···M14	19.17	-4.64	1.47	1.22
M1···M15	8.26	-4.89	0.63	1.29
M1···M16	9.65	-0.34	0.74	0.09
Total	195.88	-56.84	15.00	15.00

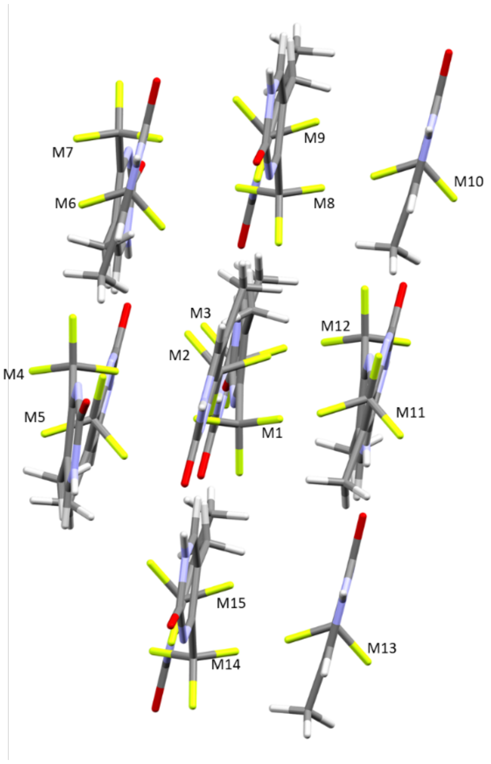

Monomer	Symmetry code	Monomer	Symmetry code
M1	x,y,z	M9	1/2-x,-1/2+y,1/2-z
M2	1/2-x,1.5-y,1-z	M10	x,1-y,-1/2+z
M3	1/2-x,1/2-y,1-z	M11	x,y,z
M4	-x,1-y,1-z	M12	1/2-x,1/2-y,1-z
M5	-1/2+x,-1/2+y,z	M13	x,1-y,1/2+z
M6	-1/2+x,1.5-y,-1/2+z	M14	x,1-y,1/2+z
M7	-x,y,1/2-z	M15	1/2-x,-1/2+y,1.5-z
M8	x,1-y,-1/2+z		

Figure S22. Supramolecular cluster and symmetry codes for the monomers of compound **13B**.

Table S20. Contact area ($C_{M1\cdots MN}$), stabilization energy ($G_{M1\cdots MN}$), normalized contact area and stabilization energy data ($NC_{M1\cdots MN}$ and $NG_{M1\cdots MN}$) between the dimers of the supramolecular cluster for **13B**.

Dimer	$C_{M1\cdots MN}$ (Å^2)	$G_{M1\cdots MN}$ (kcal mol^{-1})	$NC_{M1\cdots MN}$	$NG_{M1\cdots MN}$
M1···M2	14.44	-0.61	1.04	0.16
M1···M3	8.26	-5.29	0.59	1.39
M1···M4	8.77	-1.41	0.63	0.37

M1···M5	22.82	-9.83	1.64	2.58
M1···M6	0.28	-0.69	0.02	0.18
M1···M7	19.01	-3.10	1.37	0.81
M1···M8	12.01	-1.27	0.86	0.33
M1···M9	20.01	-10.83	1.44	2.84
M1···M10	9.65	-0.34	0.69	0.09
M1···M11	13.27	-2.78	0.95	0.73
M1···M12	30.90	-11.04	2.22	2.89
M1···M13	4.13	-0.34	0.30	0.09
M1···M14	12.01	-1.27	0.86	0.33
M1···M15	19.17	-4.64	1.38	1.22
Total	194.73	-53.43	14.00	14.00

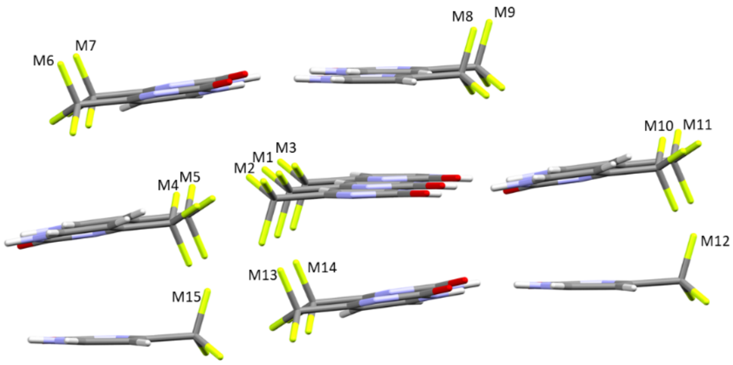

Monomer	Symmetry code	Monomer	Symmetry code
M1	x,y,z	M9	-x,-y,1-z
M2	1+x,y,z	M10	2-x,1-y,1-z
M3	-1+x,y,z	M11	1-x,1-y,1-z
M4	2-x,-y,-z	M12	1-x,1-y,1-z
M5	1-x,-y,-z	M13	x,y,z
M6	x,-1+y,z	M14	-1+x,y,z
M7	-1+x,-1+y,z	M15	1-x,-y,-z
M8	1-x,-y,1-z		

Figure S23. Supramolecular cluster and symmetry codes for the monomers of compound **14A**.

Table S21. Contact area ($C_{M1\cdots MN}$), stabilization energy ($G_{M1\cdots MN}$), normalized contact area and stabilization energy data ($NC_{M1\cdots MN}$ and $NG_{M1\cdots MN}$) between the dimers of the supramolecular cluster for **14A**.

Dimer	$C_{M1\cdots MN}$ (Å^2)	$G_{M1\cdots MN}$ (kcal mol^{-1})	$NC_{M1\cdots MN}$	$NG_{M1\cdots MN}$
M1···M2	17.9	-9.20	1.41	2.31
M1···M3	17.9	-9.20	1.41	2.31
M1···M4	12.69	-0.58	1.00	0.15
M1···M5	6.79	-0.88	0.53	0.22
M1···M6	10.89	-0.11	0.86	0.03
M1···M7	1.54	0.01	0.12	0.00
M1···M8	24.17	-10.38	1.90	2.60
M1···M9	15.12	-2.10	1.19	0.53
M1···M10	0.85	1.86	0.07	-0.57
M1···M11	16.13	-16.63	1.27	4.17
M1···M12	0.39	-2.65	0.03	0.66
M1···M13	27.94	-1.52	2.20	0.38
M1···M14	20.69	-3.61	1.63	0.91
M1···M15	4.71	-0.81	0.37	0.20
Total	177.71	-55.81	14.00	14.00

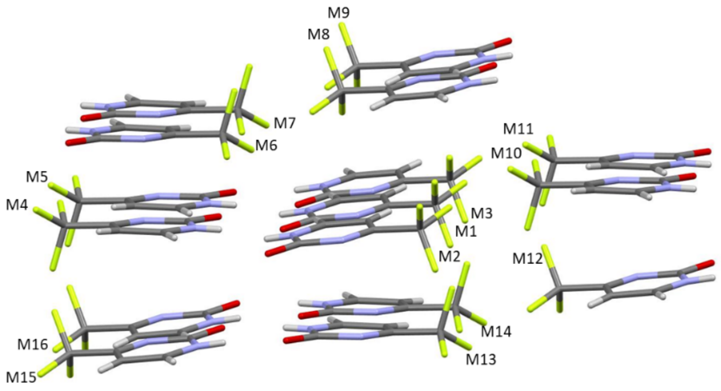

Monomer	Symmetry code	Monomer	Symmetry code
M1	x,y,z	M9	1-x,1-y,-z
M2	1+x,y,z	M10	2-x,-y,-z
M3	-1+x,y,z	M11	1-x,-y,-z

M4	1-x,1-y,1-z	M12	2-x,-y,-z
M5	-x,1-y,1-z	M13	1+x,y,z
M6	1+x,1+y,z	M14	x,y,z
M7	x,1+y,z	M15	2-x,1-y,1-z
M8	2-x,1-y,-z	M16	1-x,1-y,1-z

Figure S24. Supramolecular cluster and symmetry codes for the monomers of compound **14B**.

Table S22. Contact area ($C_{M1\cdots MN}$), stabilization energy ($G_{M1\cdots MN}$), normalized contact area and stabilization energy data ($NC_{M1\cdots MN}$ and $NG_{M1\cdots MN}$) between the dimers of the supramolecular cluster for **14B**.

Dimer	$C_{M1\cdots MN}$ (Å^2)	$G_{M1\cdots MN}$ (kcal mol^{-1})	$NC_{M1\cdots MN}$	$NG_{M1\cdots MN}$
M1···M2	18.5	-8.88	1.54	2.65
M1···M3	18.5	-8.88	1.54	2.65
M1···M4	16.13	-16.63	1.35	4.96
M1···M5	0.85	1.77	0.07	-0.53
M1···M6	1.54	0.01	0.13	0.00
M1···M7	10.89	-0.11	0.91	0.03
M1···M8	24.1	-5.77	2.01	1.72
M1···M9	11.04	-2.13	0.92	0.64
M1···M10	12.69	-0.58	1.06	0.17
M1···M11	6.79	-0.88	0.57	0.26
M1···M12	8.28	-0.52	0.69	0.16
M1···M13	20.69	-3.61	1.73	1.08
M1···M14	27.94	-1.52	2.33	0.45
M1···M15	1.03	0.16	0.08	-0.05
M1···M16	0.76	-2.69	0.06	0.80
Total	179.73	-50.29	15.00	15.00

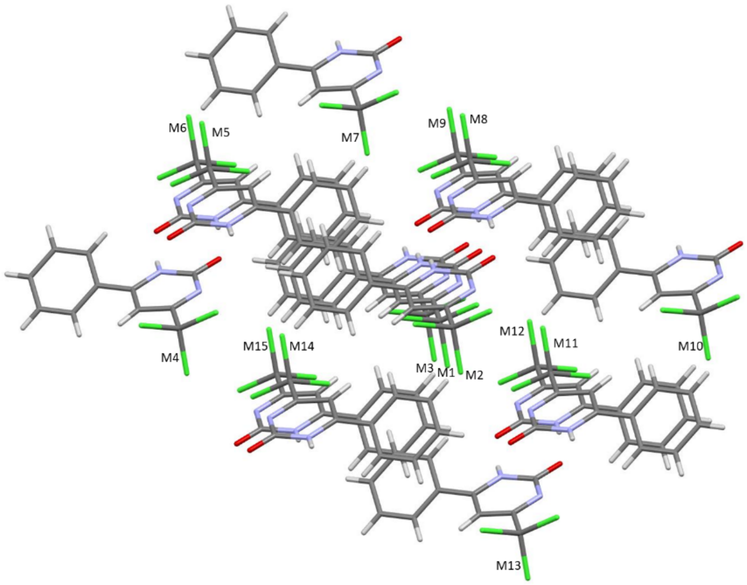

Monomer	Symmetry code	Monomer	Symmetry code
M1	x,y,z	M9	-x,2-y,-z
M2	1+x,y,z	M10	1+x,y,-1+z
M3	-1+x,y,z	M11	2-x,1-y,-z
M4	-1+x,y,1+z	M12	1-x,1-y,-z
M5	-x,2-y,1-z	M13	1+x,-1+y,z
M6	-1-x,2-y,1-z	M14	1-x,1-y,1-z
M7	-1+x,1+y,z	M15	-x,1-y,1-z
M8	1-x,2-y,-z		

Figure S25. Supramolecular cluster and symmetry codes for the monomers of compound **15**.

Table S23. Contact area ($C_{M1\cdots MN}$), stabilization energy ($G_{M1\cdots MN}$), normalized contact area and stabilization energy data ($NC_{M1\cdots MN}$ and $NG_{M1\cdots MN}$) between the dimers of the supramolecular cluster for **15**.

Dimer	$C_{M1\cdots MN}$ (Å^2)	$G_{M1\cdots MN}$ (kcal mol^{-1})	$NC_{M1\cdots MN}$	$NG_{M1\cdots MN}$
M1···M2	13.93	-2.93	0.65	0.61
M1···M3	13.93	-2.93	0.65	0.61
M1···M4	20.81	-5.58	0.97	1.16
M1···M5	54.07	-9.95	2.52	2.08
M1···M6	15.17	-1.59	0.71	0.33
M1···M7	8.34	-0.69	0.39	0.14

M1···M8	28.44	-4.95	1.33	1.03
M1···M9	20.33	-16.49	0.95	3.44
M1···M10	20.81	-5.58	0.97	1.16
M1···M11	12.82	-0.59	0.60	0.12
M1···M12	10.01	-4.16	0.47	0.87
M1···M13	8.34	-0.69	0.39	0.14
M1···M14	31.31	-5.73	1.46	1.20
M1···M15	41.72	-5.73	1.95	1.09
Total	300.03	-67.09	14.00	14.00

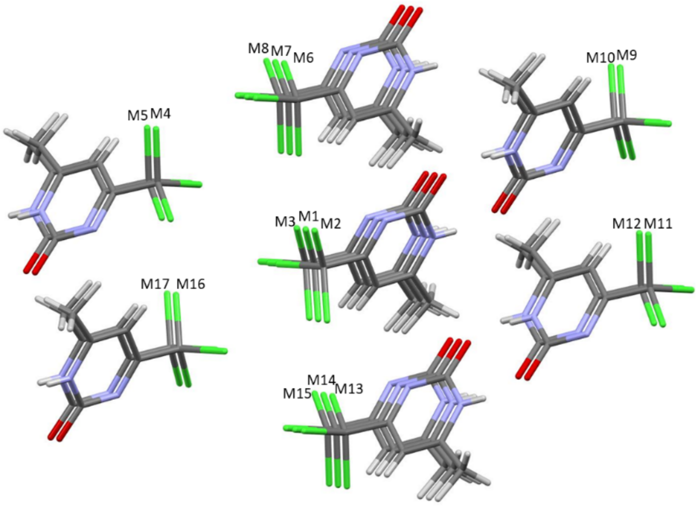

Monomer	Symmetry code	Monomer	Symmetry code
M1	x,y,z	M10	x,-1/2-y,-1/2+z
M2	x,1+y,z	M11	1-x,1-y,-z
M3	x,-1+y,z	M12	1-x,-y,-z
M4	-x,1-y,-z	M13	1-x,1/2+y,1/2-z
M5	-x,-y,-z	M14	1-x,-1/2+y,1/2-z
M6	-x,1/2+y,-1/2-z	M15	x,1.5-y,1/2+z
M7	-x,-1/2+y,-1/2-z	M16	x,1/2-y,1/2+z
M8	x,1.5-y,-1/2+z	M17	x,-1/2-y,1/2+z
M9	x,1/2-y,-1/2+z		

Figure S26. Supramolecular cluster and symmetry codes for the monomers of compound **16**.

Table S24. Contact area ($C_{M1\cdots MN}$), stabilization energy ($G_{M1\cdots MN}$), normalized contact area and stabilization energy data ($NC_{M1\cdots MN}$ and $NG_{M1\cdots MN}$) between the dimers of the supramolecular cluster for **16**.

Dimer	$C_{M1\cdots MN}$ (Å^2)	$G_{M1\cdots MN}$ (kcal mol^{-1})	$NC_{M1\cdots MN}$	$NG_{M1\cdots MN}$
M1···M2	26.27	-3.29	1.79	1.05
M1···M3	26.27	-3.29	1.79	1.05
M1···M4	15.53	-1.15	1.06	0.37
M1···M5	8.77	-1.41	0.60	0.45
M1···M6	7.53	-0.52	0.51	0.17
M1···M7	7.53	-0.52	0.51	0.17
M1···M8	4.13	0.29	0.28	-0.09
M1···M9	33.66	0.29	2.29	-0.09
M1···M10	13.46	-8.92	0.92	2.84
M1···M11	0.72	-2.48	0.05	0.79
M1···M12	17.45	-17.81	1.19	5.65
M1···M13	11.31	-0.98	0.77	0.31
M1···M14	11.31	-0.98	0.77	0.31
M1···M15	4.13	-0.98	0.28	0.31
M1···M16	33.66	0.29	2.29	-0.09
M1···M17	13.46	-8.92	0.92	2.84
Total	235.19	-50.31	16.00	16.00

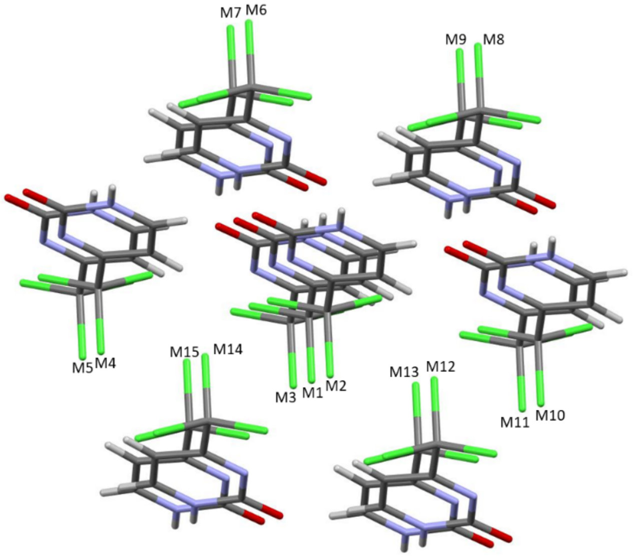

Monomer	Symmetry code	Monomer	Symmetry code
M1	x,y,z	M9	2-x,1-y,-z
M2	x,1+y,z	M10	1+x,y,z
M3	x,-1+y,z	M11	1+x,-1+y,z
M4	-1+x,1+y,z	M12	2-x,1-y,1-z
M5	-1+x,y,z	M13	2-x,-y,1-z
M6	1-x,2-y,-z	M14	1-x,1-y,1-z
M7	1-x,1-y,-z	M15	1-x,-y,1-z
M8	2-x,2-y,-z		

Figure S27. Supramolecular cluster and symmetry codes for the monomers of compound **17**.

Table S25. Contact area ($C_{M1\cdots MN}$), stabilization energy ($G_{M1\cdots MN}$), normalized contact area and stabilization energy data ($NC_{M1\cdots MN}$ and $NG_{M1\cdots MN}$) between the dimers of the supramolecular cluster for **17**.

Dimer	$C_{M1\cdots MN}$ (Å^2)	$G_{M1\cdots MN}$ (kcal mol^{-1})	$NC_{M1\cdots MN}$	$NG_{M1\cdots MN}$
M1···M2	13.81	-1.34	0.94	0.32
M1···M3	13.81	-1.34	0.94	0.32
M1···M4	2.9	-0.81	0.20	0.19
M1···M5	22.39	-9.04	1.52	2.17
M1···M6	14.46	-18.25	1.05	4.38

M1···M7	21.43	-6.79	1.45	1.63
M1···M8	0.36	2.13	0.02	-0.51
M1···M9	23.07	-4.97	1.57	1.19
M1···M10	22.39	-9.04	1.52	2.17
M1···M11	2.9	-0.81	0.20	0.19
M1···M12	17.34	-2.37	1.18	0.57
M1···M13	13.47	-0.68	0.91	0.16
M1···M14	24.98	-4.16	1.70	1.00
M1···M15	11.89	-0.83	0.81	0.20
Total	206.2	-58.30	14.00	14.00

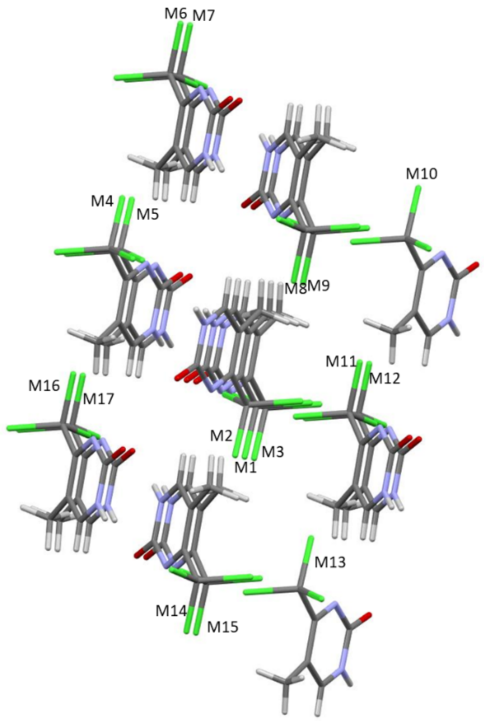

Monomer	Symmetry code	Monomer	Symmetry code
M1	x,y,z	M10	1-x,-y,-z
M2	x,1+y,z	M11	1-x,1/2+y,1/2-z
M3	x,-1+y,z	M12	1-x,-1/2+y,1/2-z
M4	-x,1-y,-z	M13	1-x,-y,1-z
M5	-x,-y,-z	M14	x,1/2-y,1/2+z
M6	-x,1/2+y,-1/2-z	M15	x,-1/2-y,1/2+z
M7	-x,-1/2+y,-1/2-z	M16	-x,1/2+y,1/2-z

M8	x,1/2-y,-1/2+z	M17	-x,-1/2+y,1/2-z
M9	x,-1/2-y,-1/2+z		

Figure S28. Supramolecular cluster and symmetry codes for the monomers of compound **18**.

Table S26. Contact area ($C_{M1\cdots MN}$), stabilization energy ($G_{M1\cdots MN}$), normalized contact area and stabilization energy data ($NC_{M1\cdots MN}$ and $NG_{M1\cdots MN}$) between the dimers of the supramolecular cluster for **18**.

Dimer	$C_{M1\cdots MN}$ (Å^2)	$G_{M1\cdots MN}$ (kcal mol^{-1})	$NC_{M1\cdots MN}$	$NG_{M1\cdots MN}$
M1···M2	5.33	-0.69	0.37	0.21
M1···M3	5.33	-0.69	0.37	0.21
M1···M4	17.94	-17.42	1.25	5.28
M1···M5	39.29	-6.72	2.73	2.04
M1···M6	0.24	0.69	0.02	-0.21
M1···M7	0.24	0.69	0.02	-0.21
M1···M8	23.88	-8.59	1.66	2.60
M1···M9	15.23	-0.97	1.06	0.29
M1···M10	22.16	-2.22	1.54	0.67
M1···M11	21.11	-3.52	1.47	1.07
M1···M12	21.11	-3.52	1.47	1.07
M1···M13	11.26	-0.38	0.78	0.12
M1···M14	23.88	-8.59	1.66	2.60
M1···M15	15.23	-0.97	1.06	0.29
M1···M16	3.97	0.04	0.28	-0.01
M1···M17	3.97	0.04	0.28	-0.01
Total	230.17	-52.82	16.00	16.00

5. Crystallization mechanisms

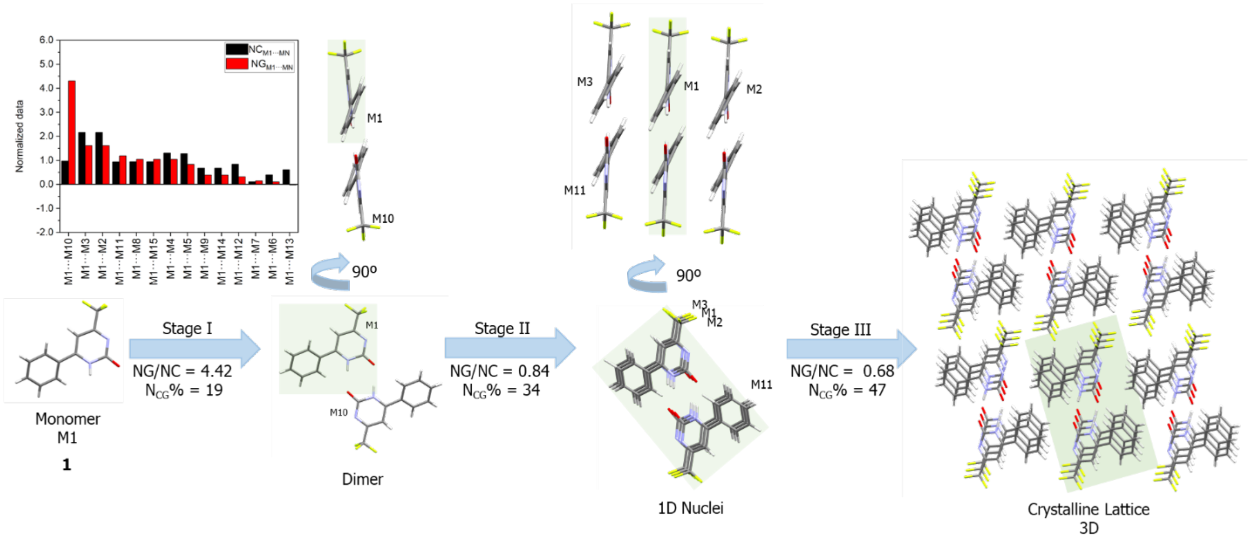

Figure S29. Normalized data and proposed crystallization mechanism for compound **1**.

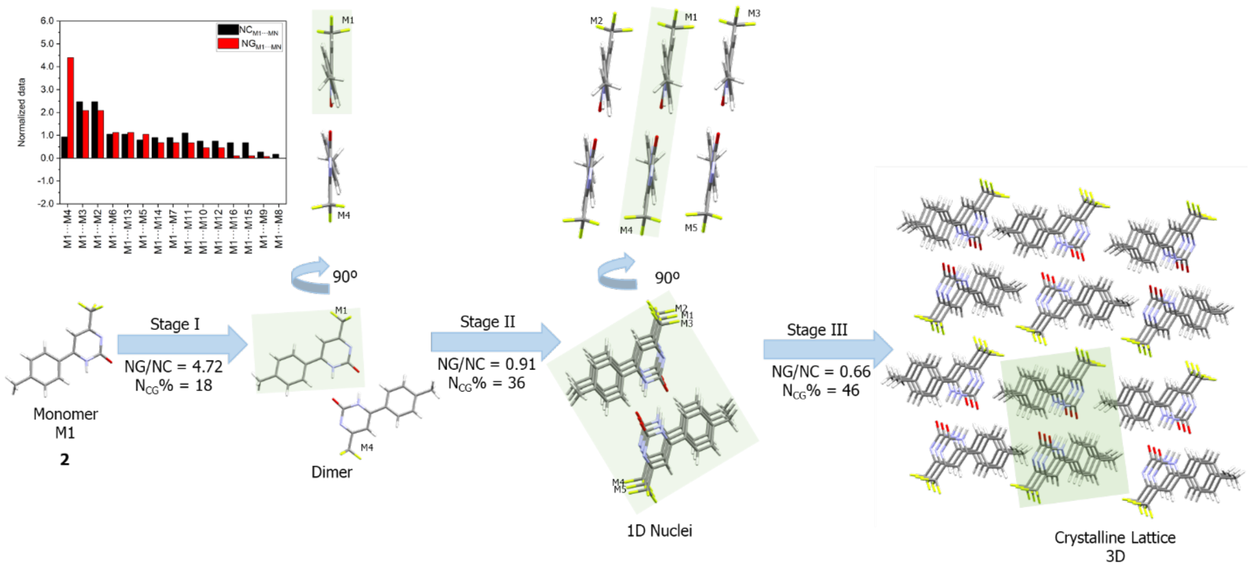

Figure S30. Normalized data and proposed crystallization mechanism for compound **2**.

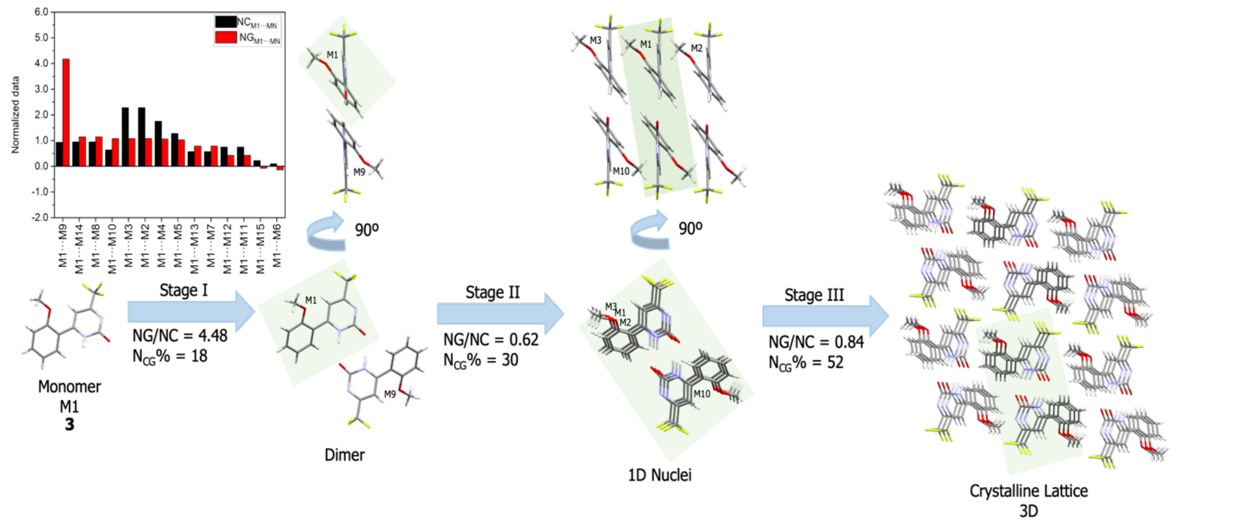

Figure S31. Normalized data and proposed crystallization mechanism for compound **3**.

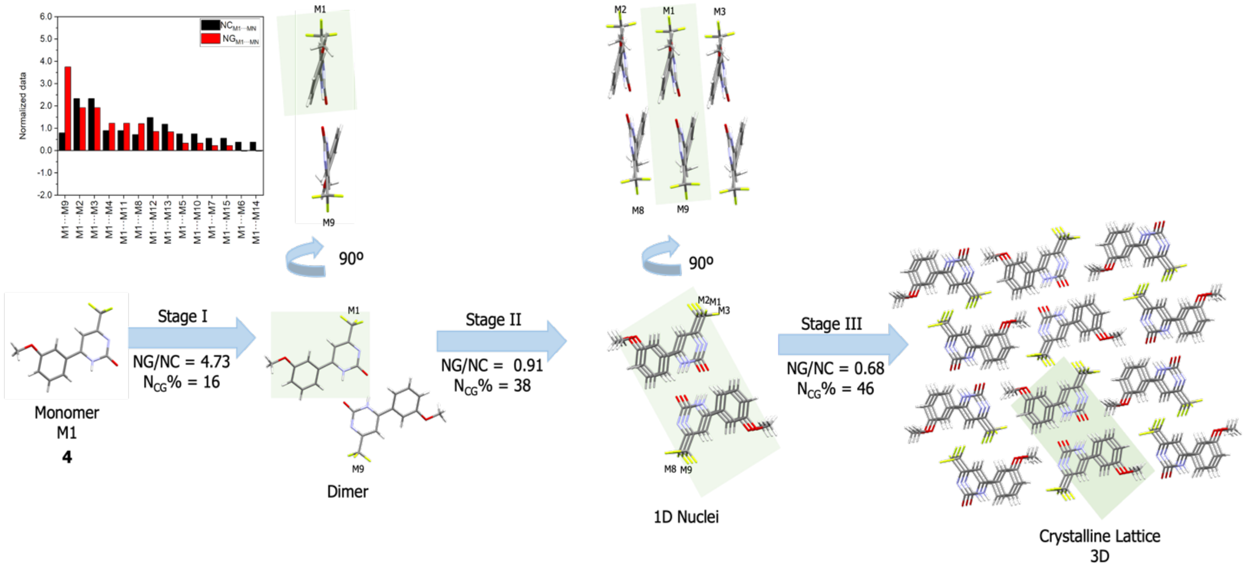

Figure S32. Normalized data and proposed crystallization mechanism for compound **4**.

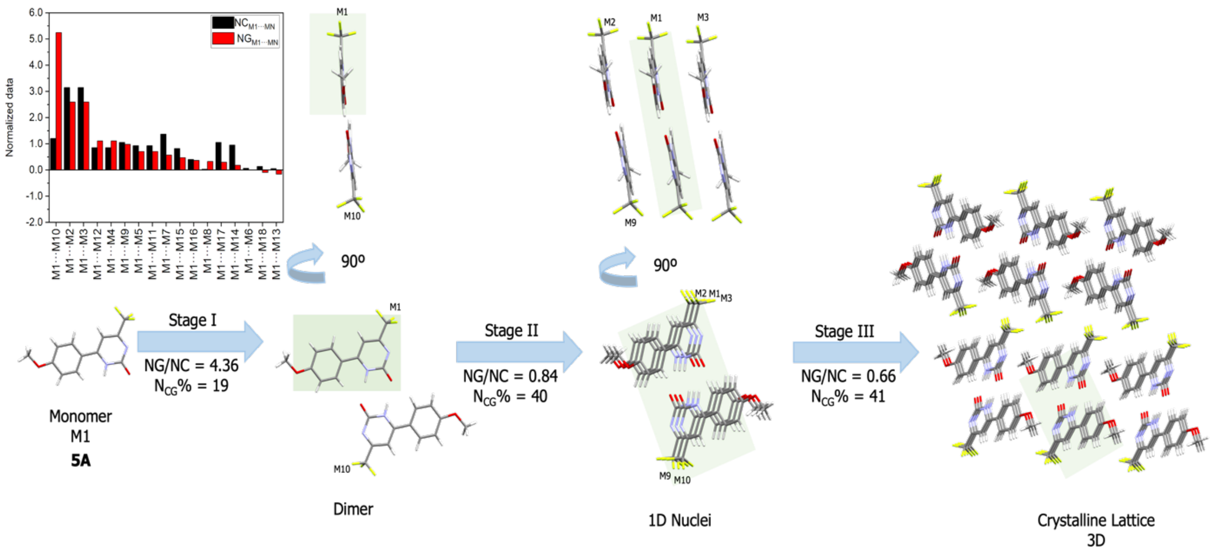

Figure S33. Normalized data and proposed crystallization mechanism for compound **5A**.

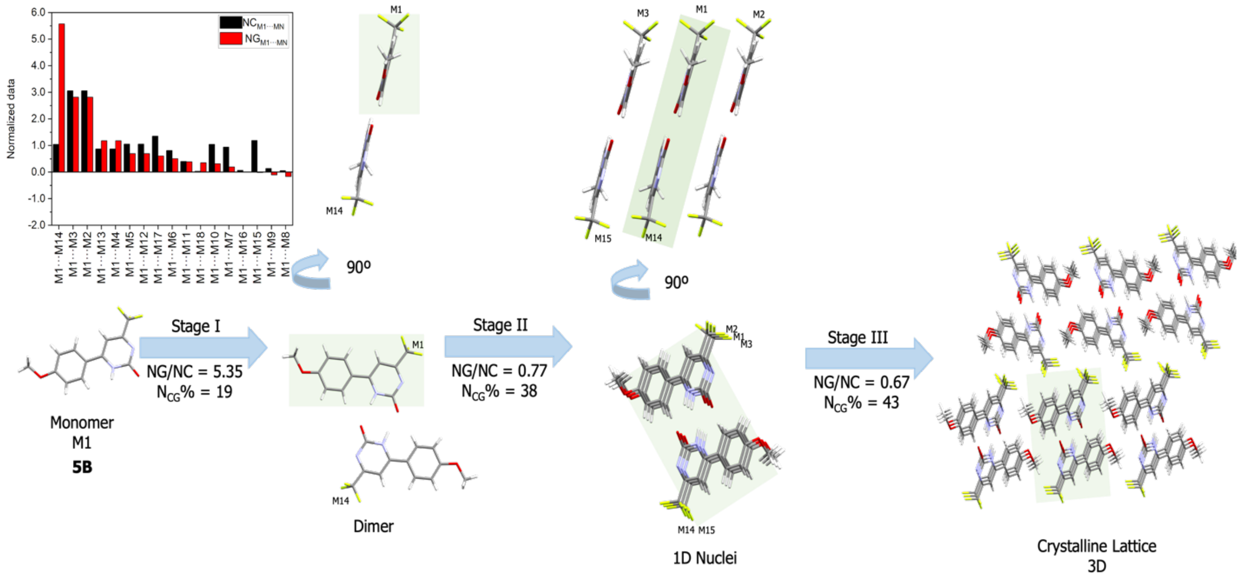

Figure S34. Normalized data and proposed crystallization mechanism for compound **5B**.

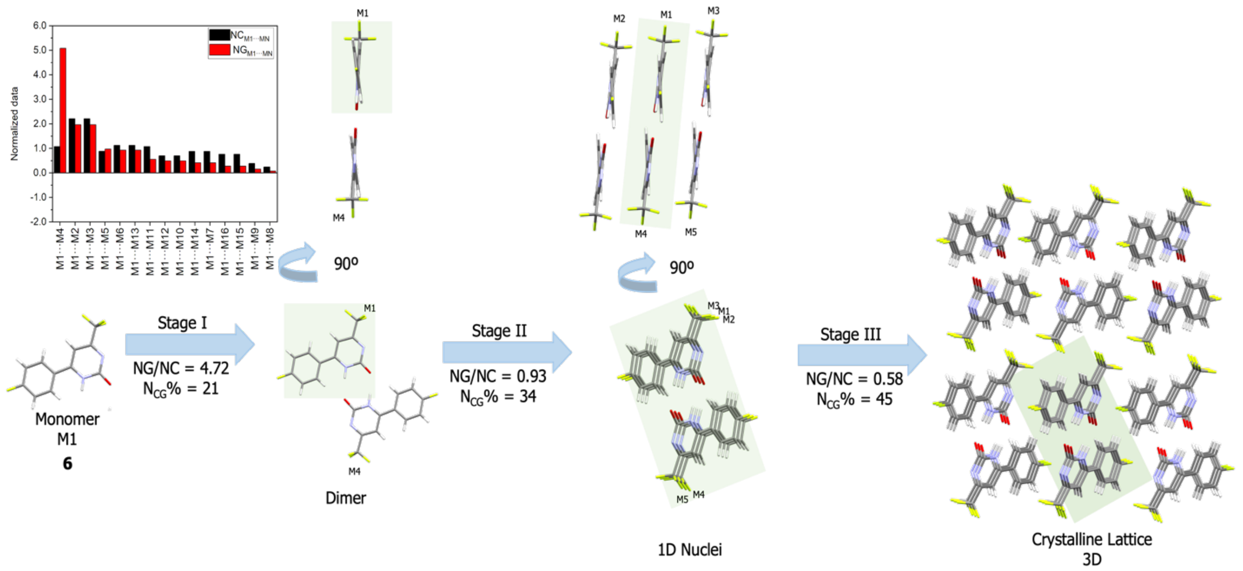

Figure S35. Normalized data and proposed crystallization mechanism for compound **6**.

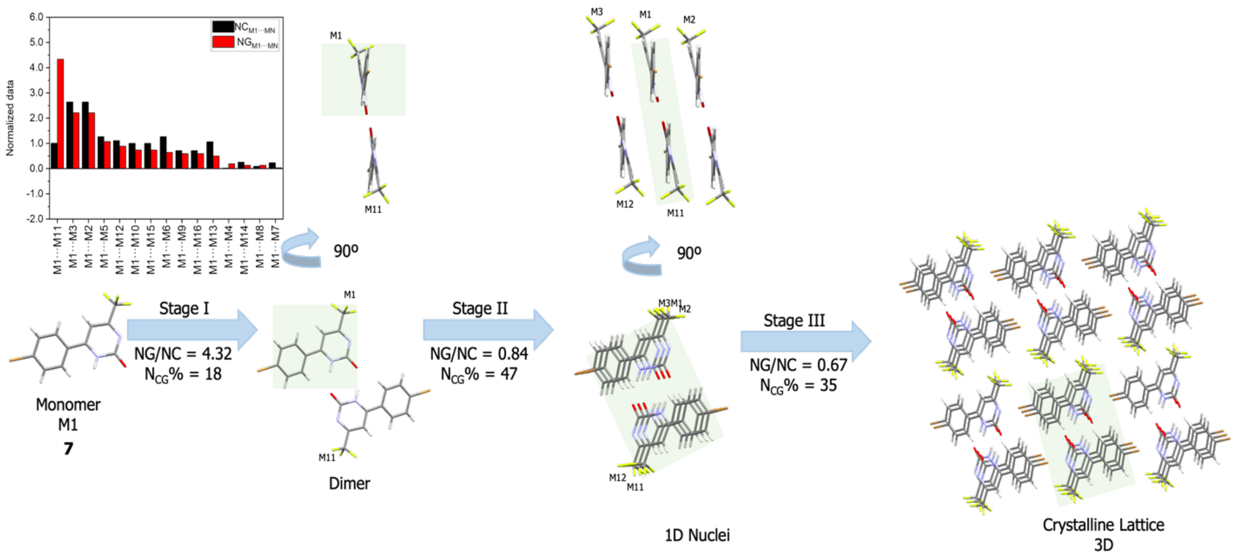

Figure S36. Normalized data and proposed crystallization mechanism for compound **7**.

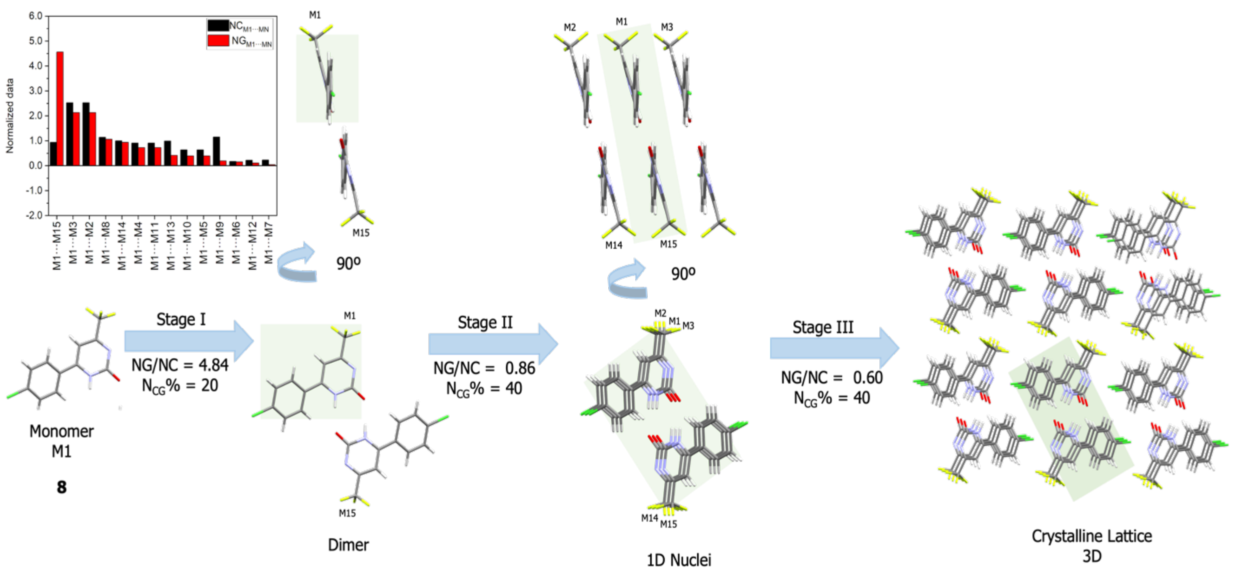

Figure S37. Normalized data and proposed crystallization mechanism for compound **8**.

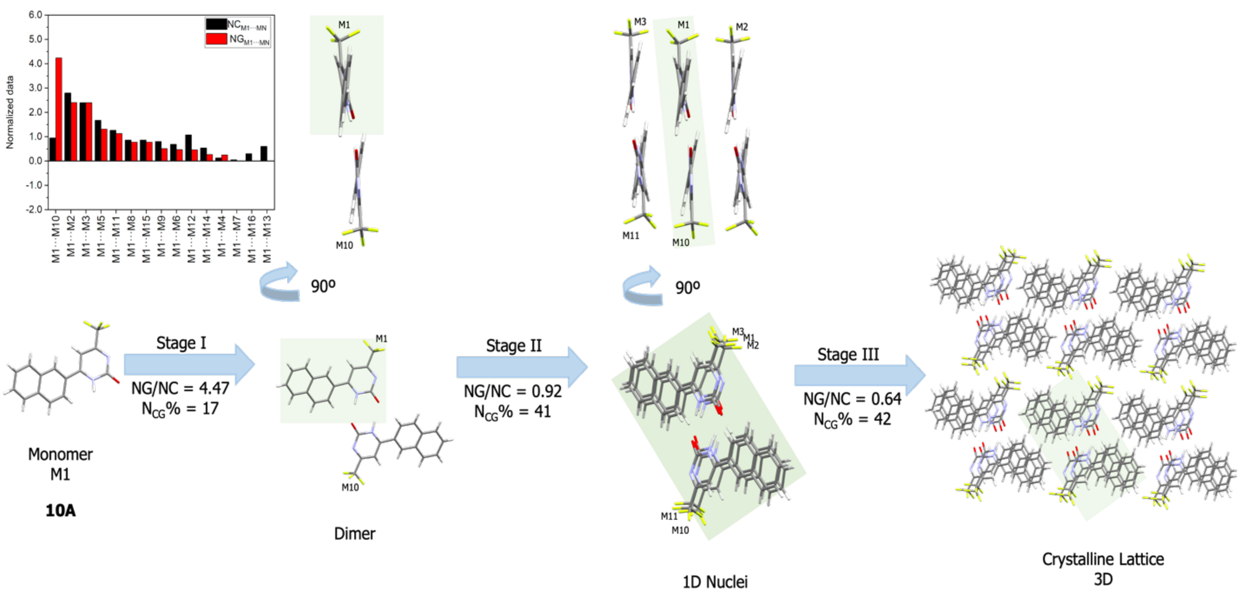

Figure S38. Normalized data and proposed crystallization mechanism for compound **10A**.

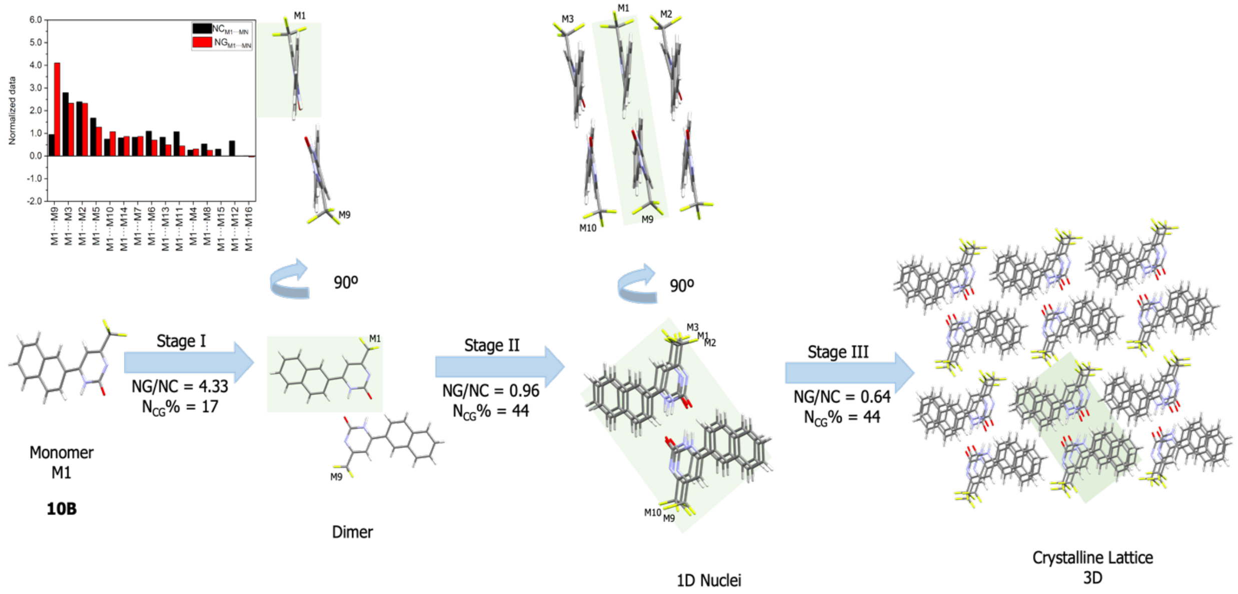

Figure S39. Normalized data and proposed crystallization mechanism for compound **10B**.

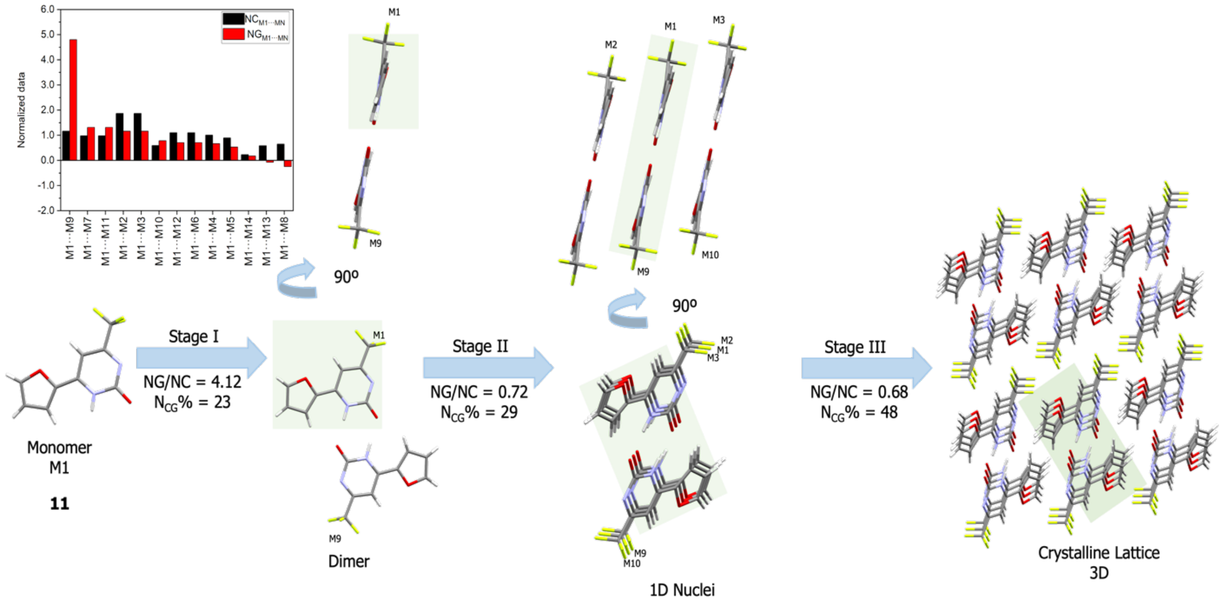

Figure S40. Normalized data and proposed crystallization mechanism for compound **11**.

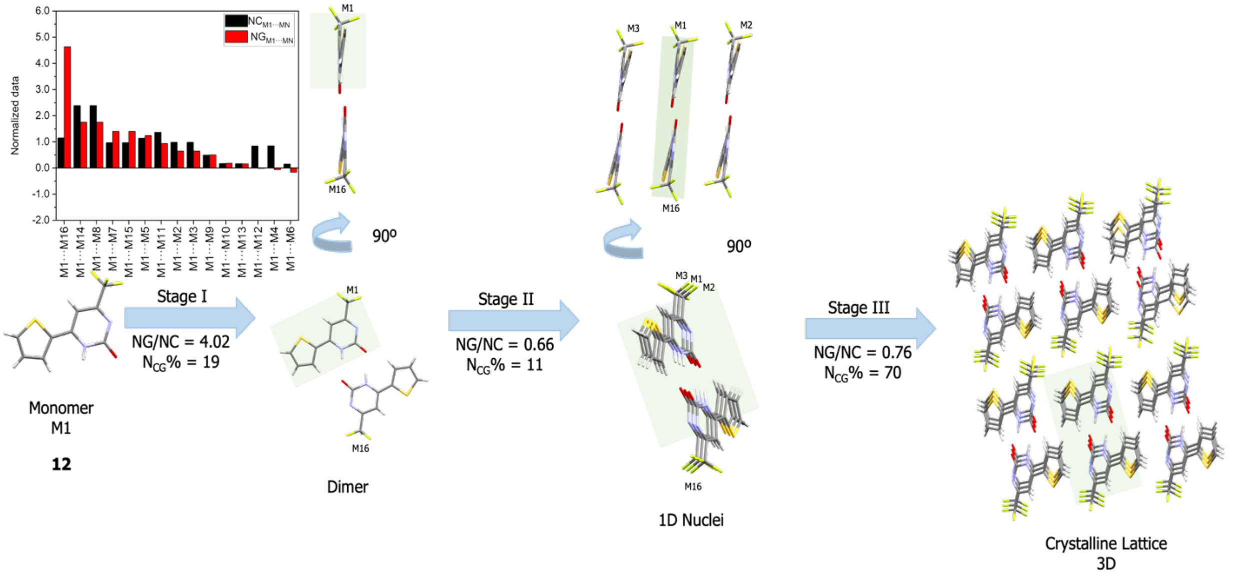

Figure S41. Normalized data and proposed crystallization mechanism for compound **12**.

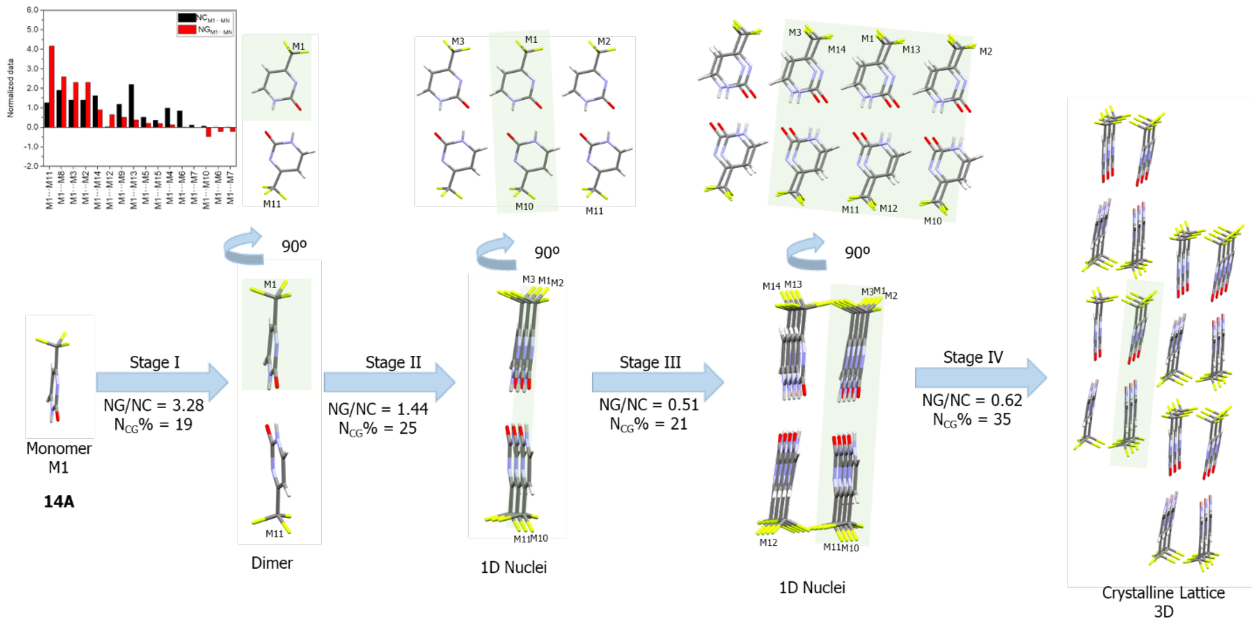

Figure S42. Normalized data and proposed crystallization mechanism for compound **14A**.

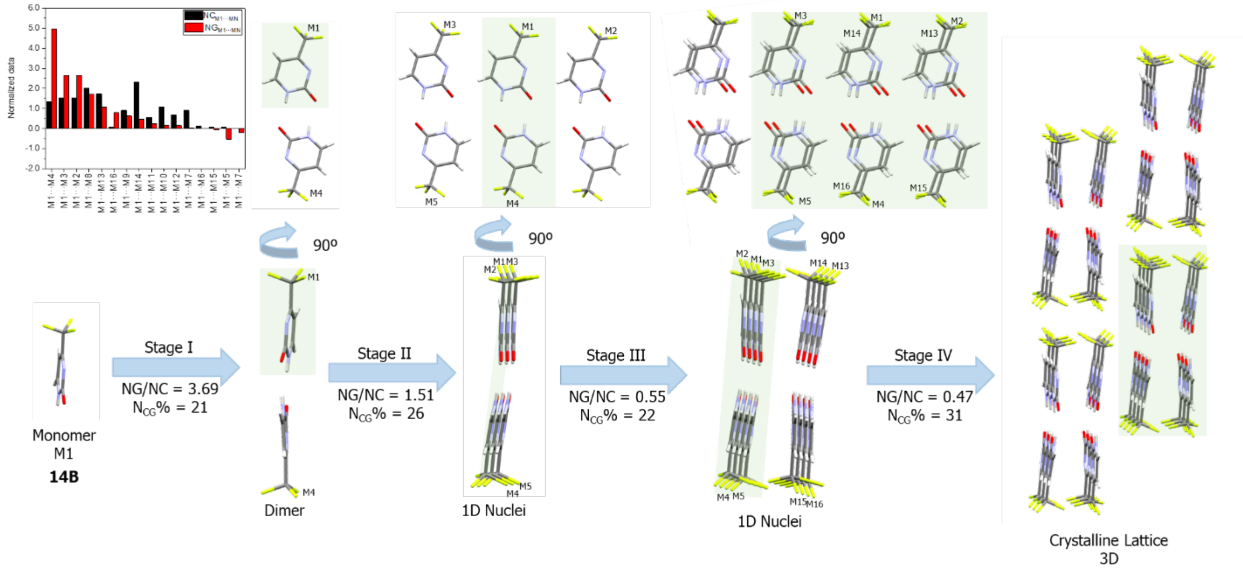

Figure S43. Normalized data and proposed crystallization mechanism for compound **14B**.

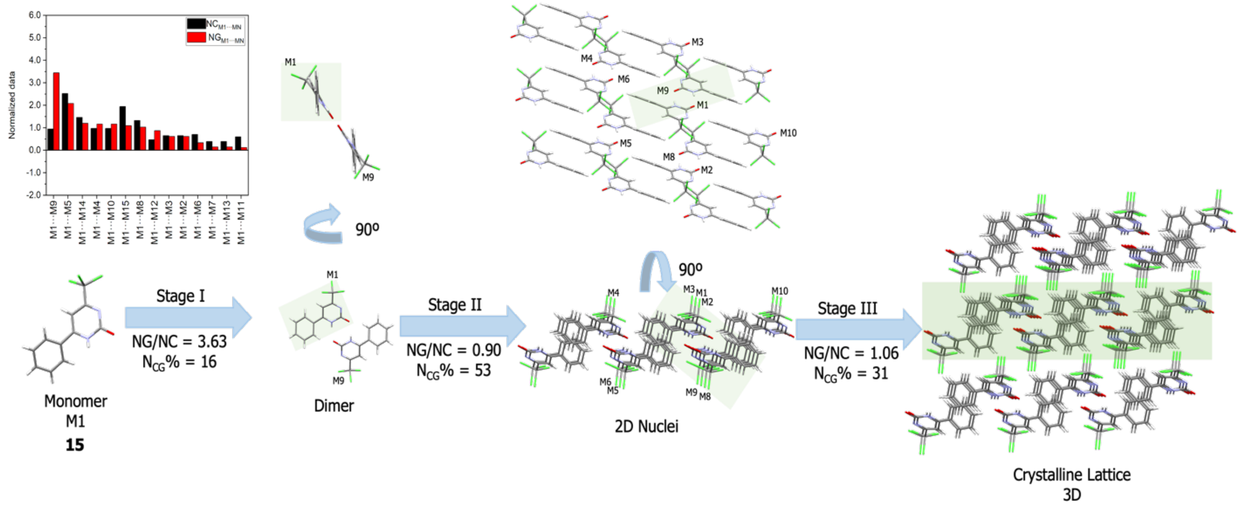

Figure S44. Normalized data and proposed crystallization mechanism for compound **15**.

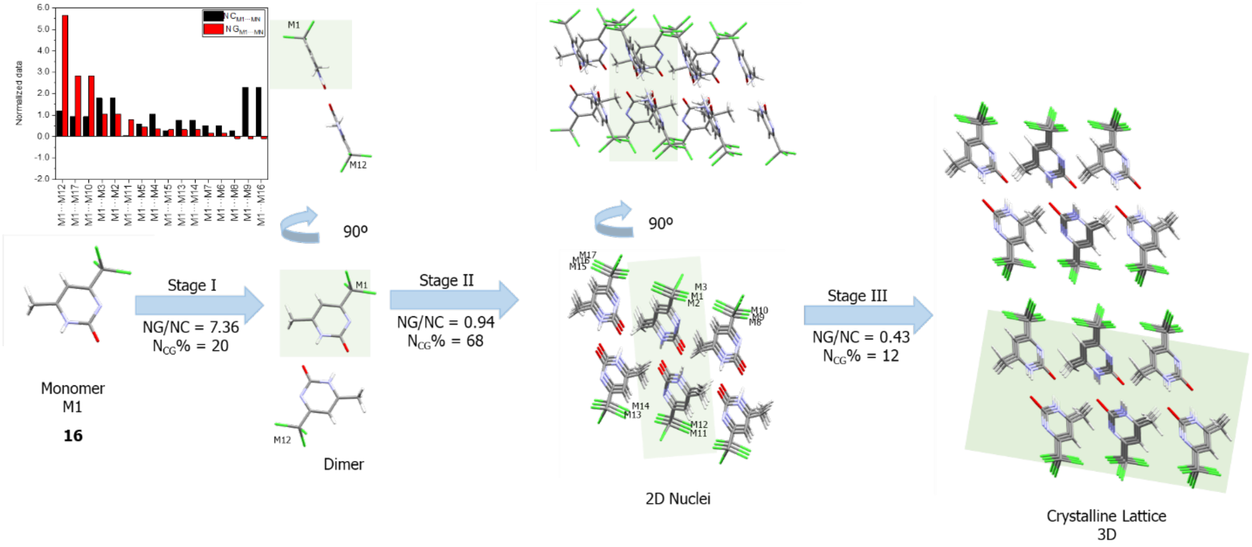

Figure S45. Normalized data and proposed crystallization mechanism for compound **16**.

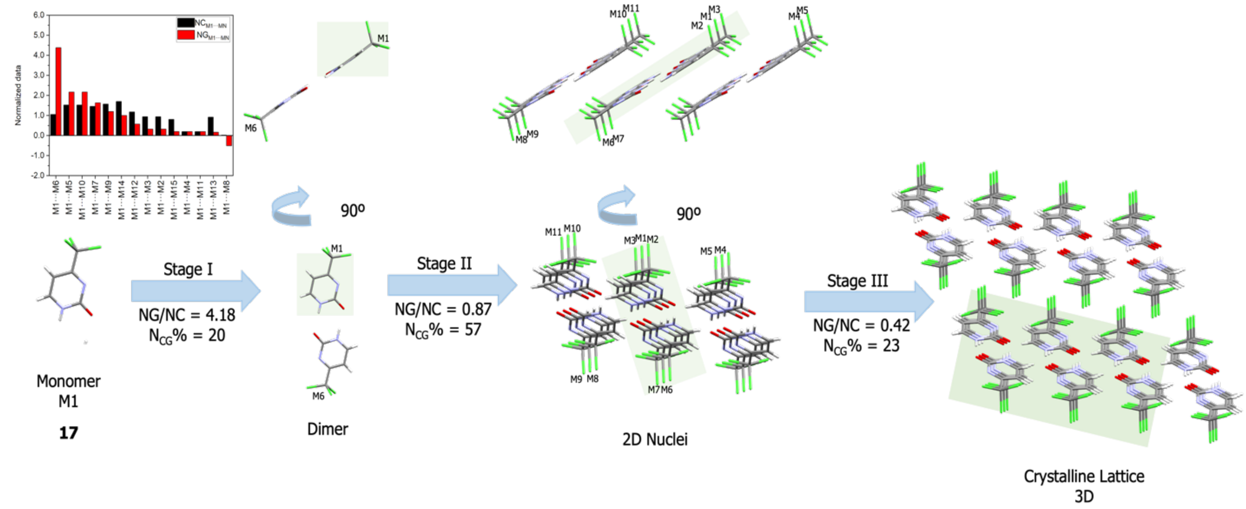

Figure S46. Normalized data and proposed crystallization mechanism for compound **17**.

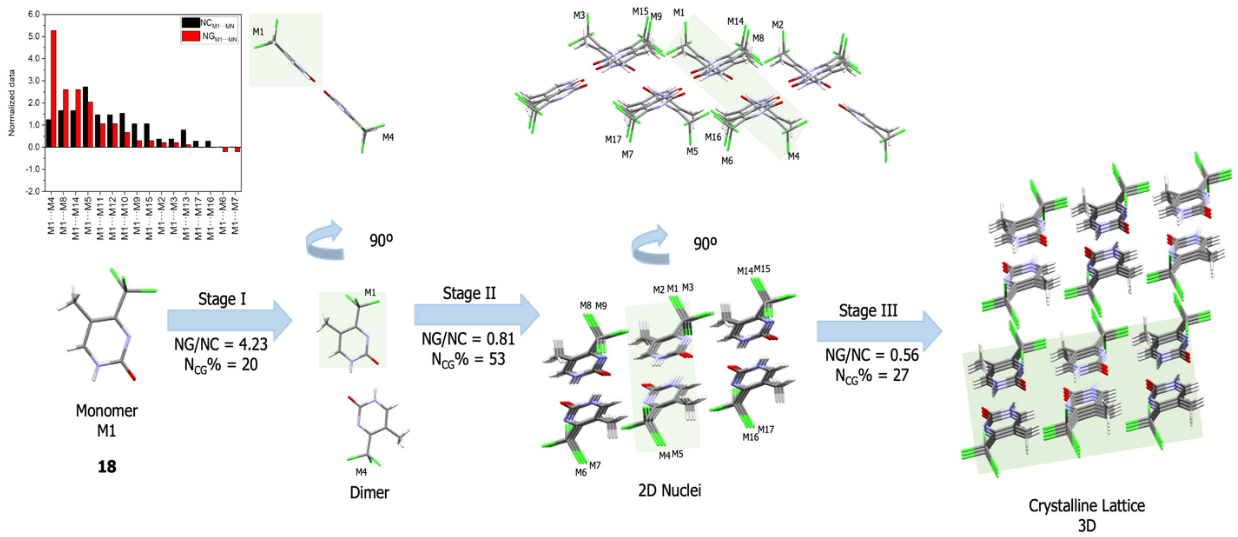

Figure S47. Proposed crystallization mechanism for compound **18**.

6. QTAIM Data

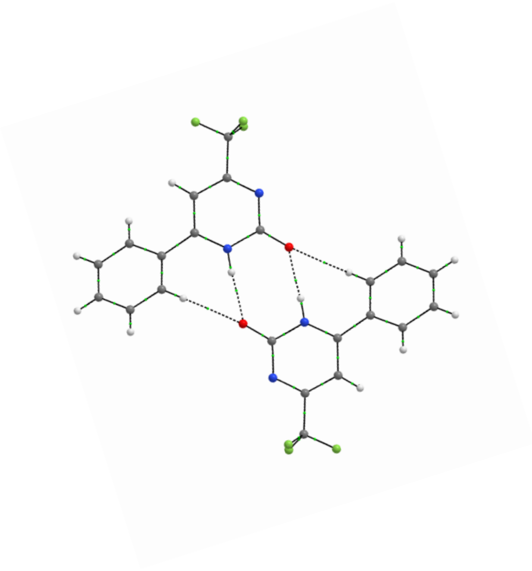

Figure S48. Intermolecular interactions for compound **1**, dimer M1···M10.

Table S27. QTAIM data for compound **1**, dimer M1···M10.

Atoms	Interaction	ρ[a]	DelSqRho[b]	Ellipticity[c]	K[d]	V[e]	G[f]	BPL[g]	G_{AI}[h]
C7-H7···O1-C1	CH···O	0.008023	0.031644	0.093500	-0.000988	-0.005936	0.006923	4.810028	-2.60
N1-H1···O1-C1	NH···O	0.025377	0.094066	0.018227	-0.002380	-0.018757	0.021137	3.793081	-8.25
C1-O1···H1-N1	NH···O	0.025377	0.094066	0.018227	-0.002380	-0.018757	0.021137	3.793081	-8.25
C1-O1···H7-C7	CH···O	0.008023	0.031644	0.093500	-0.000988	-0.005936	0.006923	4.810028	-2.60
Total	4	0.066800							-21.72

[a] ρ, represents electron density at a bond critical point (BCP). [b] $\nabla^2\rho$, represents the Laplacian of the electron density (DelSqRho). [c] ε, represents the ellipticity. [d,e,f] K, V, G, represent the different energy densities. [g] BPL, represents length. [h] G_{AI} represents interaction energy in kcal mol^{-1}.

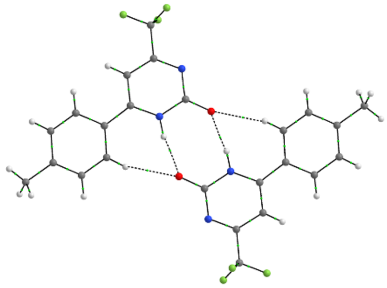

Figure S49. Intermolecular interactions for compound **2**, dimer M1···M4.

Table S28. QTAIM data for compound **2**, dimer M1···M4.

Atoms	Interaction	ρ[a]	DelSqRho[b]	Ellipticity[c]	K[d]	V[e]	G[f]	BPL[g]	G_{AI}[h]
C7-H7···O1-C1	CH···O	0.009451	0.034654	0.127365	-0.00078	-0.00711	0.007886	4.6489	-3.02
N1-H1···O1-C1	NH···O	0.025329	0.092504	0.017190	-0.00223	-0.01866	0.020893	3.7862	-8.10
C1-O1···H1-N1	NH···O	0.025329	0.092504	0.017190	-0.00223	-0.01866	0.020893	3.7862	-8.10
C1-O1···H7-C7	CH···O	0.009451	0.034654	0.127365	-0.00078	-0.00711	0.007886	4.6489	-3.02
Total	4	0.069560							-22.25

[a] ρ, represents electron density at a bond critical point (BCP). [b] $\nabla^2\rho$, represents the Laplacian of the electron density (DelSqRho). [c] ε, represents the ellipticity. [d,e,f] K, V, G, represent the different energy densities. [g] BPL, represents length. [h] G_{AI} represents interaction energy in kcal mol^{-1}.

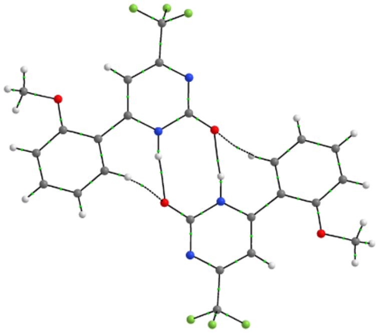

Figure S50. Intermolecular interactions for compound **3**, dimer M1···M9.

Table S29. QTAIM data for compound **3**, dimer M1···M9.

Atoms	Interaction	ρ[a]	DelSqRho[b]	Ellipticity[c]	K[d]	V[e]	G[f]	BPL[g]	G_{AI}[h]
C7-H7···O1-C1	CH···O	0.006366	0.030167	0.881333	-0.00162	-0.00431	0.005925	5.2876	-1.87
N1-H1···O1-C1	NH···O	0.032845	0.120027	0.022701	-0.00269	-0.02463	0.027317	3.5563	-9.63
C1-O1···H1-N1	NH···O	0.032845	0.120027	0.022701	-0.00269	-0.02463	0.027317	3.5563	-9.63
C1-O1···H7-C7	CH···O	0.006366	0.030167	0.881333	-0.00162	-0.00431	0.005925	5.2876	-1.87
Total	4	0.078422							-23.00

[a] ρ, represents electron density at a bond critical point (BCP). [b] $\nabla^2\rho$, represents the Laplacian of the electron density (DelSqRho). [c] ε, represents the ellipticity. [d,e,f] K, V, G, represent the different energy densities. [g] BPL, represents length. [h] G_{AI} represents interaction energy in kcal mol^{-1}.

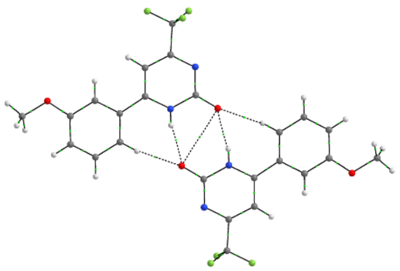

Figure S51. Intermolecular interactions for compound **4**, dimer M1···M9.

Table S29. QTAIM data for compound **4**, dimer M1···M9.

Atoms	Interaction	ρ[a]	DelSqRho[b]	Ellipticity[c]	K[d]	V[e]	G[f]	BPL[g]	G_{AI}[h]
C7-H7···O1-C1	CH···O	0.010710	0.039207	0.066908	-0.00082	-0.00817	0.008983	4.5403	-2.99
N1-H1···O1-C1	NH···O	0.021601	0.082998	0.018451	-0.00205	-0.01665	0.018698	3.9546	-6.03
C1-O1···H1-N1	NH···O	0.021601	0.082998	0.018451	-0.00205	-0.01665	0.018698	3.9546	-6.03
C1-O1···H7-C7	CH···O	0.010710	0.039207	0.066908	-0.00082	-0.00817	0.008983	4.5403	-2.99
C1-O1···O1-C1	O···O	0.006104	0.023005	38.34997	-0.00068	-0.00440	0.005076	6.1907	-1.71
Total	5	0.070726							-19.76

[a] ρ, represents electron density at a bond critical point (BCP). [b] $\nabla^2\rho$, represents the Laplacian of the electron density (DelSqRho). [c] ε, represents the ellipticity. [d,e,f] K, V, G, represent the different energy densities. [g] BPL, represents length. [h] G_{AI} represents interaction energy in kcal mol^{-1}.

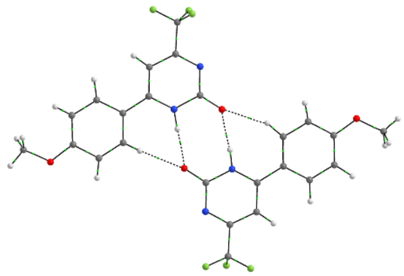

Figure S52. Intermolecular interactions for compound **5**, dimer M1···M10.

Table S30. QTAIM data for compound **5**, dimer M1···M10.

Atoms	Interaction	ρ[a]	DelSqRho[b]	Ellipticity[c]	K[d]	V[e]	G[f]	BPL[g]	G_{AI}[h]
C7'-H7'···O1-C1	CH···O	0.012791	0.047294	0.106072	-0.00099	-0.00985	0.010839	4.3518	-3.80
N1'-H1'···O1-C1	NH···O	0.028436	0.102883	0.009294	-0.00249	-0.02075	0.023236	3.6776	-8.45
C1'-O1'···H1-N1	NH···O	0.025199	0.089085	0.010247	-0.00203	-0.01822	0.020244	3.7880	-7.49
C1'-O1'···H7-C7	CH···O	0.013951	0.047911	0.085302	-0.00065	-0.01068	0.011326	4.2959	-4.15
Total	4	0.080377							-23.89

[a] ρ, represents electron density at a bond critical point (BCP). [b] $\nabla^2\rho$, represents the Laplacian of the electron density (DelSqRho). [c] ε, represents the ellipticity. [d,e,f] K, V, G, represent the different energy densities. [g] BPL, represents length. [h] G_{AI} represents interaction energy in kcal mol^{-1}.

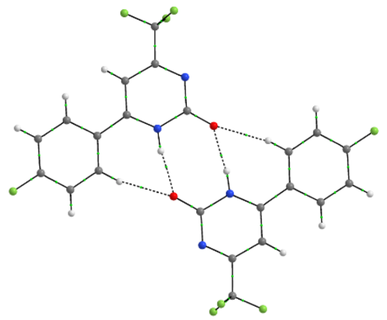

Figure S53. Intermolecular interactions for compound **6**, dimer M1···M4.

Table S31. QTAIM data for compound **6**, dimer M1···M4.

Atoms	Interaction	ρ[a]	DelSqRho[b]	Ellipticity[c]	K[d]	V[e]	G[f]	BPL[g]	G_{AI}[h]
C7-H7···O1-C1	CH···O	0.012709	0.044955	0.103789	-0.00075	-0.00974	0.010491	4.3703	-3.95
N1-H1···O1-C1	NH···O	0.028047	0.103844	0.011991	-0.00259	-0.02078	0.023371	3.6932	-8.71
C1-O1···H1-N1	NH···O	0.028047	0.103844	0.011991	-0.00259	-0.02078	0.023371	3.6932	-8.71
C1-O1···H7-C7	CH···O	0.012709	0.044955	0.103789	-0.00075	-0.00974	0.010491	4.3703	-3.95
Total	4	0.081512							-25.30

[a] ρ, represents electron density at a bond critical point (BCP). [b] $\nabla^2\rho$, represents the Laplacian of the electron density (DelSqRho). [c] ε, represents the ellipticity. [d,e,f] K, V, G, represent the different energy densities. [g] BPL, represents length. [h] G_{AI} represents interaction energy in kcal mol^{-1}.

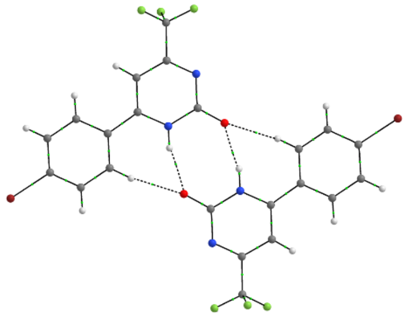

Figure S54. Intermolecular interactions for compound **7**, dimer M1···M11.

Table S32. QTAIM data for compound **7**, dimer M1···M11.

Atoms	Interaction	ρ[a]	DelSqRho[b]	Ellipticity[c]	K[d]	V[e]	G[f]	BPL[g]	G_{AI}[h]
C7-H7···O1-C1	CH···O	0.011326	0.040496	0.117530	-0.00074	-0.00864	0.009381	4.4815	-3.59
N1-H1···O1-C1	NH···O	0.026042	0.099211	0.013717	-0.00256	-0.01968	0.022243	3.7679	-8.26
C1-O1···H1-N1	NH···O	0.026042	0.099211	0.013717	-0.00256	-0.01968	0.022243	3.7679	-8.26
C1-O1···H7-C7	CH···O	0.011326	0.040496	0.117530	-0.00074	-0.00864	0.009381	4.4815	-3.59
Total	4	0.074736							-23.69

[a] ρ, represents electron density at a bond critical point (BCP). [b] $\nabla^2\rho$, represents the Laplacian of the electron density (DelSqRho). [c] ε, represents the ellipticity. [d,e,f] K, V, G, represent the different energy densities. [g] BPL, represents length. [h] G_{AI} represents interaction energy in kcal mol^{-1}.

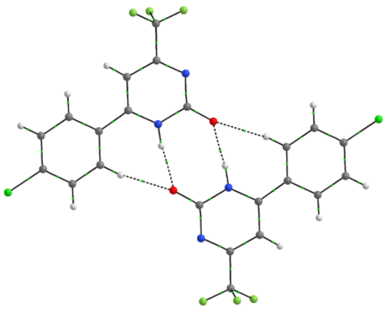

Figure S55. Intermolecular interactions for compound **8**, dimer M1···M15.

Table S33. QTAIM data for compound **8**, dimer M1···M15.

Atoms	Interaction	ρ[a]	DelSqRho[b]	Ellipticity[c]	K[d]	V[e]	G[f]	BPL[g]	G_{AI}[h]
C7-H7···O1-C1	CH···O	0.011227	0.040440	0.102433	-0.00077	-0.00857	0.009342	4.4899	-3.47
N1-H1···O1-C1	NH···O	0.027675	0.103801	0.016071	-0.00267	-0.02062	0.023285	3.7108	-8.57
C1-O1···H1-N1	NH···O	0.027675	0.103801	0.016071	-0.00267	-0.02062	0.023285	3.7108	-8.57
C1-O1···H7-C7	CH···O	0.011227	0.040440	0.102433	-0.00077	-0.00857	0.009342	4.4899	-3.47
Total	4	0.077804							-24.08

[a] ρ, represents electron density at a bond critical point (BCP). [b] $\nabla^2\rho$, represents the Laplacian of the electron density (DelSqRho). [c] ε, represents the ellipticity. [d,e,f] K, V, G, represent the different energy densities. [g] BPL, represents length. [h] G_{AI} represents interaction energy in kcal mol^{-1}.

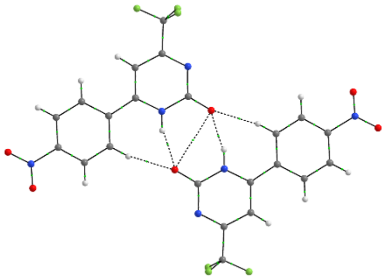

Figure S56. Intermolecular interactions for compound **9**, dimer M1···M8.

Table S34. QTAIM data for compound **9**, dimer M1···M8.

Atoms	Interaction	ρ[a]	DelSqRho[b]	Ellipticity[c]	K[d]	V[e]	G[f]	BPL[g]	G_{AI}[h]
C7'-H7'···O1'-C1'	CH···O	0.013117	0.045817	0.131154	-0.00073	-0.01000	0.010725	4.3437	-3.68
N1'-H1'···O1'-C1'	NH···O	0.028914	0.115287	0.010320	-0.00314	-0.02255	0.025687	3.6746	-8.10
C1'-O1'···H1'-N1'	NH···O	0.007196	0.026450	8.885788	-0.00058	-0.00546	0.006034	6.0214	-2.02
C1'-O1'···H7'-C7'	CH···O	0.028914	0.115287	0.010320	-0.00314	-0.02255	0.025687	3.6746	-8.10
C1'-O1'···O1'-C1'	O···O	0.013117	0.045817	0.131154	-0.00073	-0.01000	0.010725	4.3437	-3.68
Total	5	0.091258							-25.58

[a] ρ, represents electron density at a bond critical point (BCP). [b] $\nabla^2\rho$, represents the Laplacian of the electron density (DelSqRho). [c] ε, represents the ellipticity. [d,e,f] K, V, G, represent the different energy densities. [g] BPL, represents length. [h] G_{AI} represents interaction energy in kcal mol^{-1}.

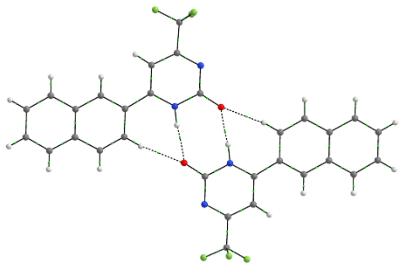

Figure S57. Intermolecular interactions for compound **10**, dimer M1···M10.

Table S35. QTAIM data for compound **10**, dimer M1···M10.

Atoms	Interaction	ρ[a]	DelSqRho[b]	Ellipticity[c]	K[d]	V[e]	G[f]	BPL[g]	G_{AI}[h]
C7-H7···O1'-C1'	CH···O	0.010425	0.037215	0.095795	-0.00072	-0.00786	0.008584	4.5743	-3.12
N1-H1···O1'-C1'	NH···O	0.029294	0.107845	0.017000	-0.00266	-0.02164	0.024299	3.6532	-8.77
C1-O2···H1'-N1'	NH···O	0.028367	0.104006	0.012041	-0.00257	-0.02087	0.023434	3.6847	-8.49
C1-O2···H7'-C7'	CH···O	0.011652	0.041566	0.103564	-0.00073	-0.00893	0.009662	4.4469	-3.49
Total	4	0.079738							-23.86

[a] ρ, represents electron density at a bond critical point (BCP). [b] $\nabla^2\rho$, represents the Laplacian of the electron density (DelSqRho). [c] ε, represents the ellipticity. [d,e,f] K, V, G, represent the different energy densities. [g] BPL, represents length. [h] G_{AI} represents interaction energy in kcal mol^{-1}.

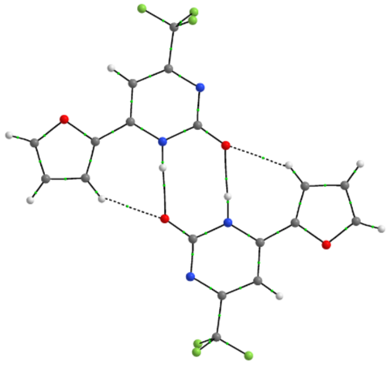

Figure S58. Intermolecular interactions for compound **11**, dimer M1···M9.

Table S36. QTAIM data for compound **11**, dimer M1···M9.

Atoms	Interaction	ρ[a]	DelSqRho[b]	Ellipticity[c]	K[d]	V[e]	G[f]	BPL[g]	G_{AI}[h]
C7-H7···O1-C1	CH···O	0.010144	0.037016	0.090203	-0.00080	-0.00766	0.008455	4.5730	-3.20
N1-H1···O1-C1	NH···O	0.032951	0.120451	0.024744	-0.00265	-0.02481	0.02746	3.5722	-10.39
C1-O1···H1-N1	NH···O	0.032951	0.120451	0.024744	-0.00265	-0.02481	0.02746	3.5722	-10.39
C1-O1···H7-C7	CH···O	0.010144	0.037016	0.090203	-0.00080	-0.00766	0.008455	4.5730	-3.20
Total	4	0.086190							-27.17

[a] ρ, represents electron density at a bond critical point (BCP). [b] $\nabla^2\rho$, represents the Laplacian of the electron density (DelSqRho). [c] ε, represents the ellipticity. [d,e,f] K, V, G, represent the different energy densities. [g] BPL, represents length. [h] G_{AI} represents interaction energy in kcal mol^{-1}.

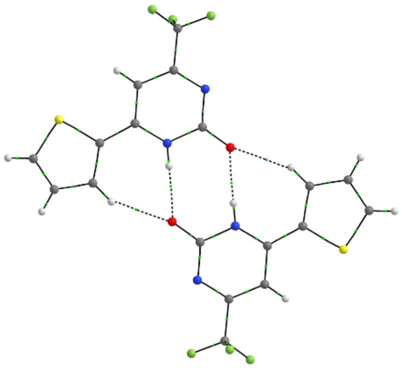

Figure S59. Intermolecular interactions for compound **12**, dimer M1···M16.

Table S37. QTAIM data for compound **12**, dimer M1···M16.

Atoms	Interaction	ρ[a]	DelSqRho[b]	Ellipticity[c]	K[d]	V[e]	G[f]	BPL[g]	G_{AI}[h]
C7-H7···O005-C1	CH···O	0.011137	0.039888	0.066679	-0.00077	-0.00843	0.009199	4.4846	-3.72
N1-H1···O005-C1	NH···O	0.022558	0.082934	0.025845	-0.00192	-0.01689	0.018813	3.9252	-7.53
C1-O005···H1-N1	NH···O	0.022558	0.082934	0.025845	-0.00192	-0.01689	0.018813	3.9252	-7.53
C1-O005···H7-C7	CH···O	0.011137	0.039888	0.066679	-0.00077	-0.00843	0.009199	4.4846	-3.72
Total	4	0.067390							-22.50

[a] ρ, represents electron density at a bond critical point (BCP). [b] $\nabla^2\rho$, represents the Laplacian of the electron density (DelSqRho). [c] ε, represents the ellipticity. [d,e,f] K, V, G, represent the different energy densities. [g] BPL, represents length. [h] G_{AI} represents interaction energy in kcal mol^{-1}.

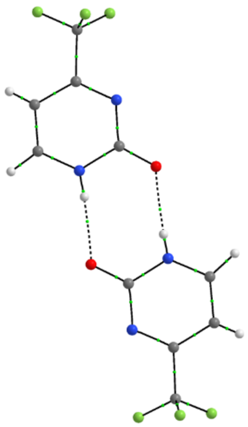

Figure S60. Intermolecular interactions for compound **14**, dimer M1···M5.

Table S38. QTAIM data for compound **14**, dimer M1···M5.

Atoms	Interaction	ρ[a]	DelSqRho[b]	Ellipticity[c]	K[d]	V[e]	G[f]	BPL[g]	G_{AI}[h]
N1-H1···O1'-C1'	NH···O	0.022614	0.090306	0.041530	-0.00251	-0.01755	0.020064	3.9650	-8.21
C1-O1···H1'-N1'	NH···O	0.023189	0.086259	0.035745	-0.00203	-0.01750	0.019532	3.9297	-8.42
Total	2	0.045803							-16.63

[a] ρ, represents electron density at a bond critical point (BCP). [b] $\nabla^2\rho$, represents the Laplacian of the electron density (DelSqRho). [c] ε, represents the ellipticity. [d,e,f] K, V, G, represent the different energy densities. [g] BPL, represents length. [h] G_{AI} represents interaction energy in kcal mol^{-1}.

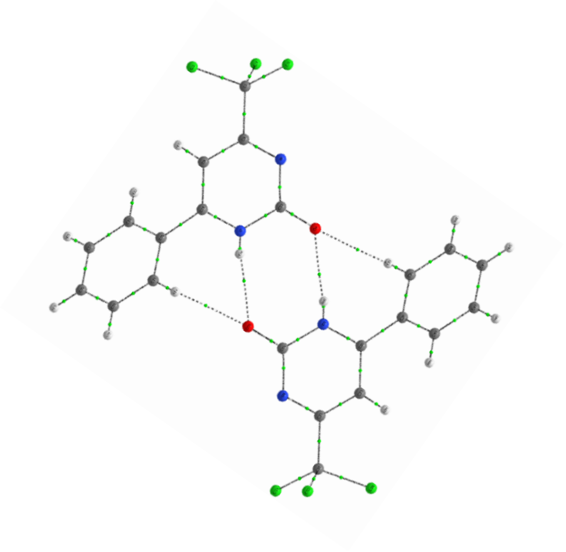

Figure S61. Intermolecular interactions for compound **15**, dimer M1···M9.

Table S39. QTAIM data for compound **15**, dimer M1···M9.

Atoms	Interaction	ρ[a]	DelSqRho[b]	Ellipticity[c]	K[d]	V[e]	G[f]	BPL[g]	G_{AI}[h]
C7-H7···O1-C1	CH···O	0.008491	0.035204	0.046145	-0.00127	-0.00627	0.007535	4.8186	-2.74
N1-H1···O1-C1	NH···O	0.017062	0.069901	0.048787	-0.00205	-0.01338	0.015429	4.2117	-5.51
C1-O1···H1-N1	NH···O	0.017062	0.069901	0.048787	-0.00205	-0.01338	0.015429	4.2117	-5.51
C1-O1···H7-C7	CH···O	0.008491	0.035204	0.046145	-0.00127	-0.00627	0.007535	4.8186	-2.74
Total	4	0.051106							-16.49

[a] ρ, represents electron density at a bond critical point (BCP). [b] $\nabla^2\rho$, represents the Laplacian of the electron density (DelSqRho). [c] ε, represents the ellipticity. [d,e,f] K, V, G, represent the different energy densities. [g] BPL, represents length. [h] G_{AI} represents interaction energy in kcal mol^{-1}.

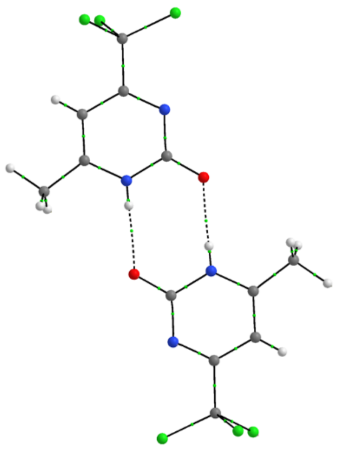

Figure S62. Intermolecular interactions for compound **16**, dimer M1···M12.

Table S40. QTAIM data for compound **16**, dimer M1···M12.

Atoms	Interaction	ρ[a]	DelSqRho[b]	Ellipticity[c]	K[d]	V[e]	G[f]	BPL[g]	G_{AI}[h]
C1-O1···N1-H1	NH···O	0.021899	0.086156	0.050067	-0.00227	-0.01701	0.019272	3.9960	-8.90
N1-H1···O1-C1	NH···O	0.021899	0.086156	0.050067	-0.00227	-0.01701	0.019272	3.9960	-8.90

Total	2	0.006431							-17.81

[a] ρ, represents electron density at a bond critical point (BCP). [b] $\nabla^2\rho$, represents the Laplacian of the electron density (DelSqRho). [c] ε, represents the ellipticity. [d,e,f] K, V, G, represent the different energy densities. [g] BPL, represents length. [h] G_{AI} represents interaction energy in kcal mol^{-1}.

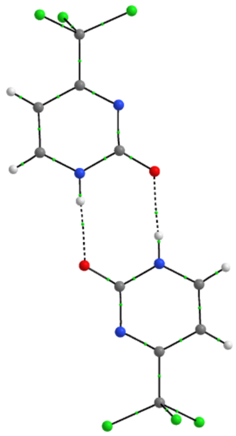

Figure S63. Intermolecular interactions for compound **17**, dimer M1···M6.

Table S41. QTAIM data for compound **17**, dimer M1···M6.

Atoms	Interaction	ρ[a]	DelSqRho[b]	Ellipticity[c]	K[d]	V[e]	G[f]	BPL[g]	G_{AI}[h]
N1-H1···O1-C1	NH···O	0.02532	0.09272	0.033463	-0.00216	-0.01885	0.021017	3.8313	-9.13
C1-O1···H1-N1	NH···O	0.02532	0.09272	0.033463	-0.00216	-0.01885	0.021017	3.8313	-9.13
Total	2	0.050640							-18.25

[a] ρ, represents electron density at a bond critical point (BCP). [b] $\nabla^2\rho$, represents the Laplacian of the electron density (DelSqRho). [c] ε, represents the ellipticity. [d,e,f] K, V, G, represent the different energy densities. [g] BPL, represents length. [h] G_{AI} represents interaction energy in kcal mol^{-1}.

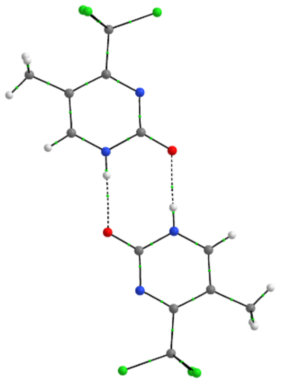

Figure S64. Intermolecular interactions for compound **18**, dimer M1···M4.

Table S42. QTAIM data for compound **18**, dimer M1···M4.

Atoms	Interaction	ρ[a]	DelSqRho[b]	Ellipticity[c]	K[d]	V[e]	G[f]	BPL[g]	G_{AI}[h]
N1-H1···O1-C1	NH···O	0.025768	0.09608	0.039417	-0.00226	-0.0195	0.021759	3.8338	-8.71
C1-O1···H1-N1	NH···O	0.025768	0.09608	0.039417	-0.00226	-0.0195	0.021759	3.8338	-8.71

Total	2	0.051536	-17.42

[a] ρ, represents electron density at a bond critical point (BCP). [b] $\nabla^2\rho$, represents the Laplacian of the electron density (DelSqRho). [c] ε, represents the ellipticity. [d,e,f] K, V, G, represent the different energy densities. [g] BPL, represents length. [h] G_{AI} represents interaction energy in kcal mol^{-1}.

7. MEP Surfaces

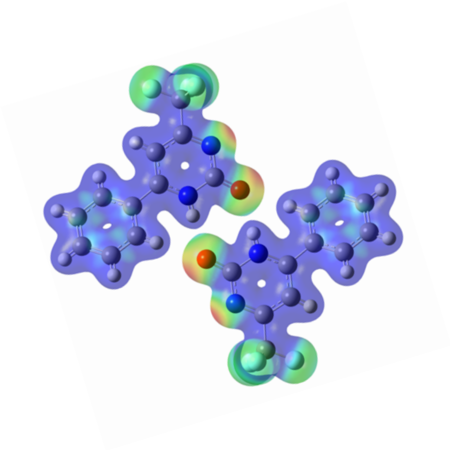

1

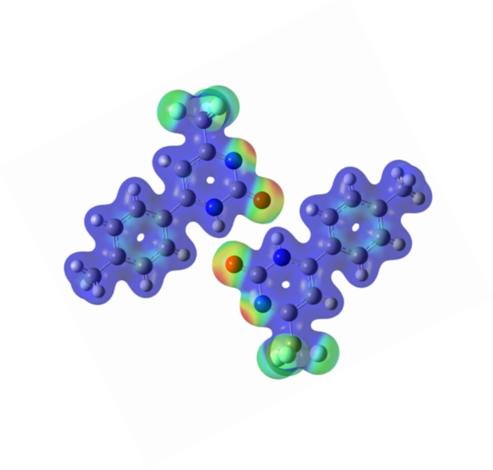

2

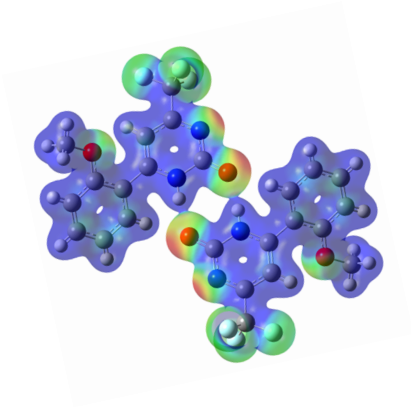

3

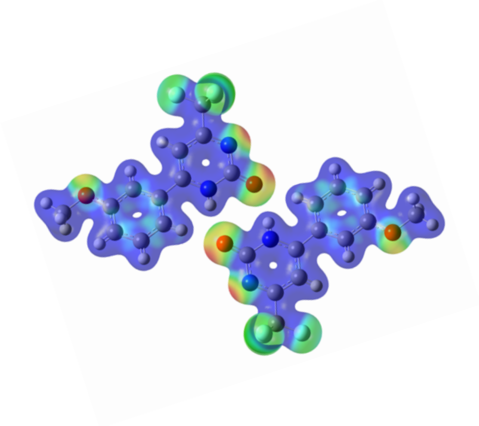

4

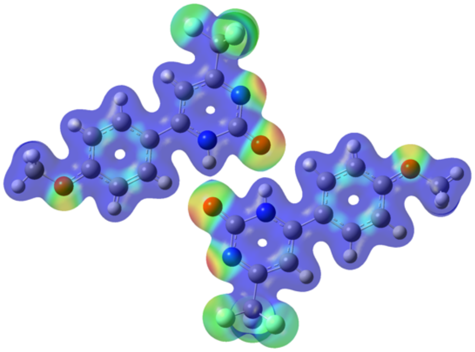

5

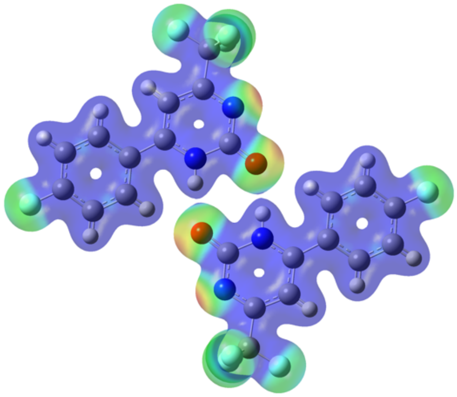

6

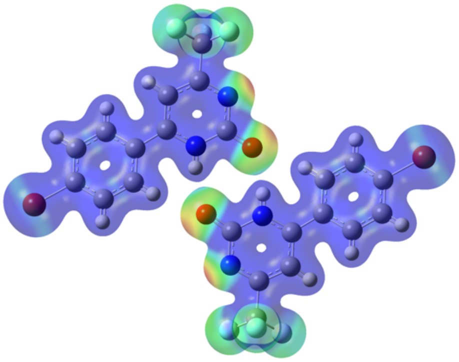

7

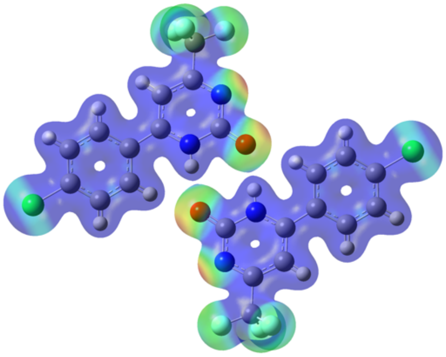

8

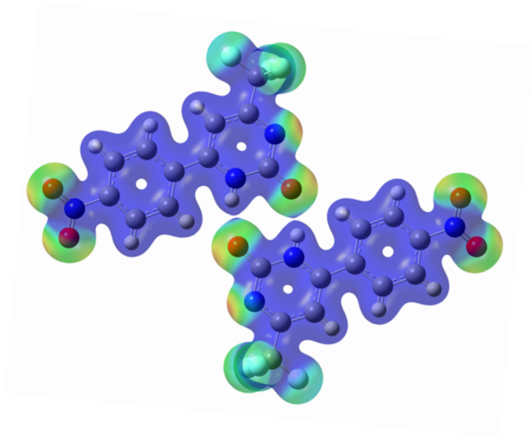

9

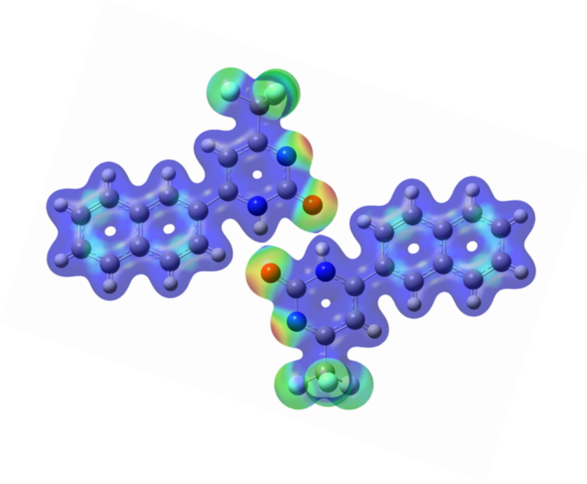

10

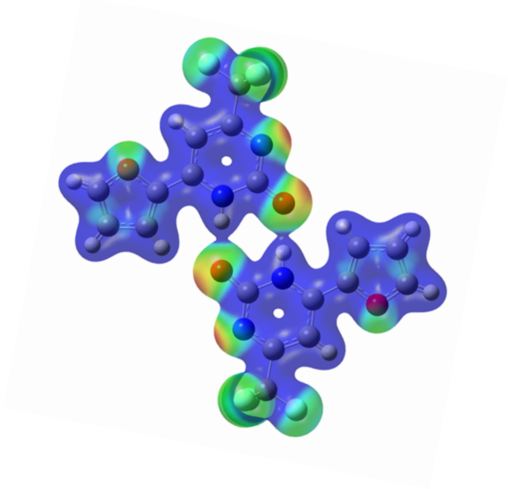

11

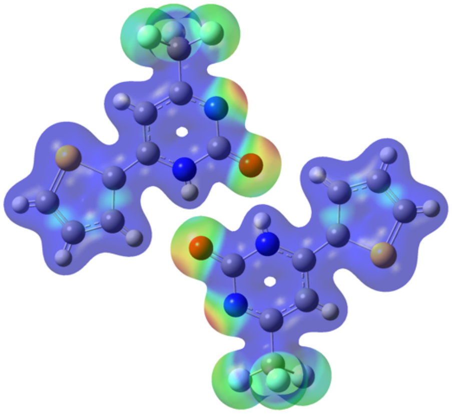

12

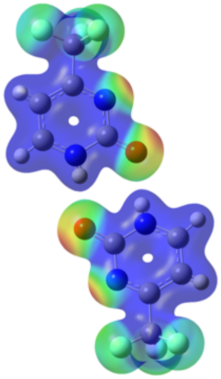

14

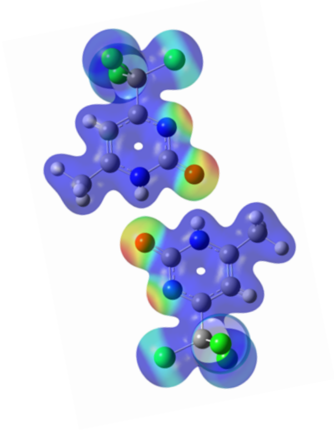

16

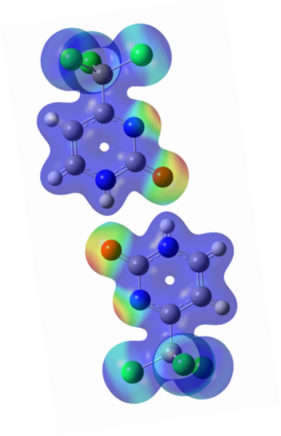

17

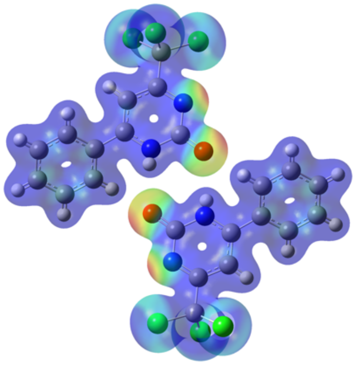

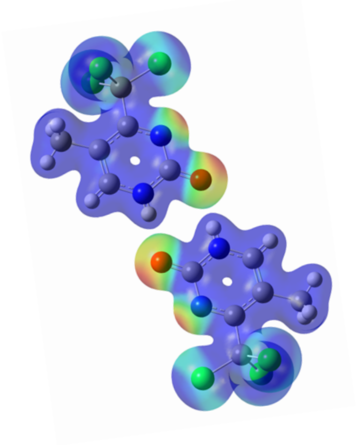

15 **18**

Figure S65. The MEP surfaces of the first dimer, the most energetic in stabilization energy terms, of compounds **1**–**18.** MEP surface: red and blue with -0.07 and 0.09 au, respectively.

8. CD NMR Data

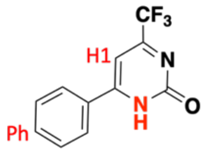

1

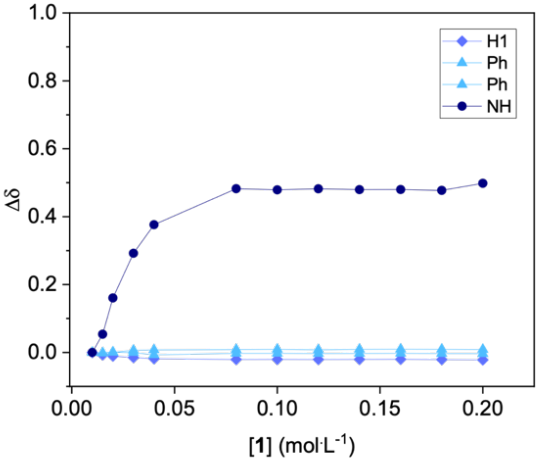

Figure S66. Correlation between the Δδ of the hydrogens of compound **1** and its concentration, data extracted from CD ^{1}H NMR at $CDCl_3$ at 298 K.

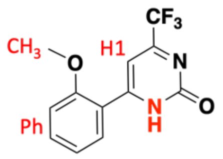

3

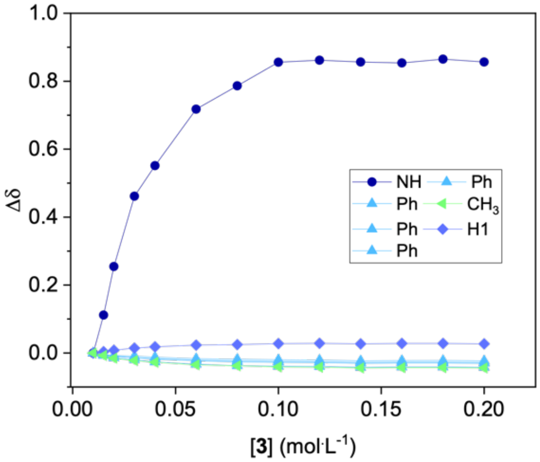

Figure S67. Correlation between the Δδ of the hydrogens of compound **3** and its concentration, data extracted from CD ^{1}H NMR at $CDCl_3$ at 298 K.

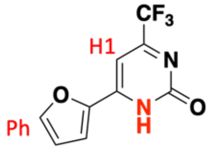

11

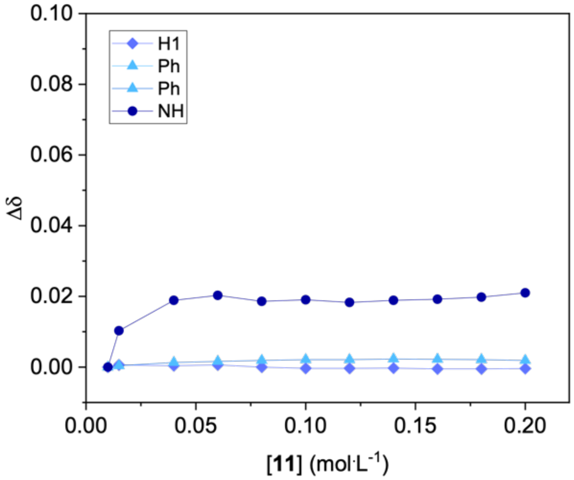

Figure S68. Correlation between the Δδ of the hydrogens of compound **11** and its concentration, data extracted from CD ^{1}H NMR at $CDCl_3$ at 298 K.

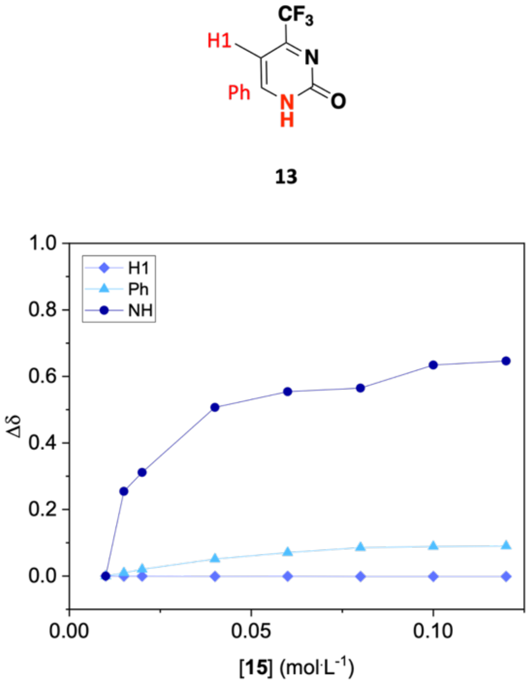

Figure S69. Correlation between the Δδ of the hydrogens of compound **13** and its concentration, data extracted from CD ^{1}H NMR at $CDCl_3$ at 298 K.

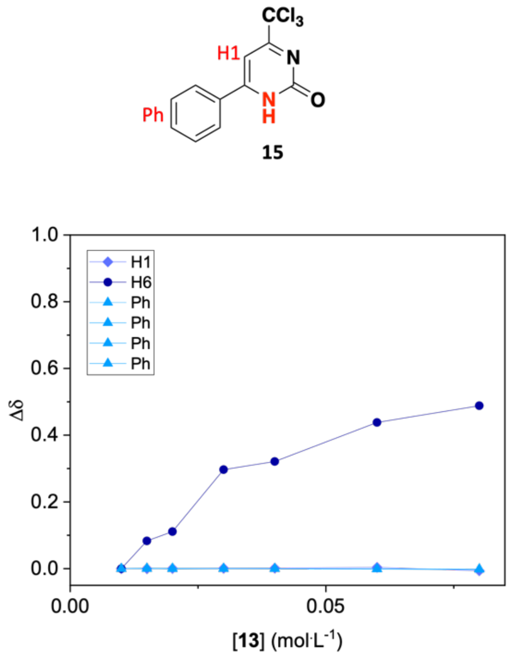

Figure S70. Correlation between the Δδ of the hydrogens of compound **15** and its concentration, data extracted from CD ^{1}H NMR at $CDCl_3$ at 298 K.

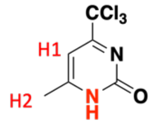

16

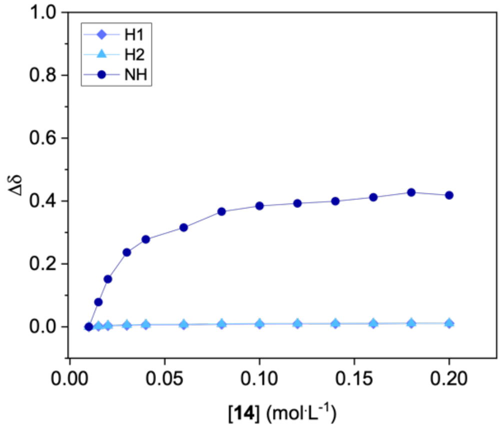

Figure S71. Correlation between the Δδ of the hydrogens of compound **16** and its concentration, data extracted from CD ^{1}H NMR at $CDCl_3$ at 298 K.

9. Solid State Nuclear Magnetic Resonance

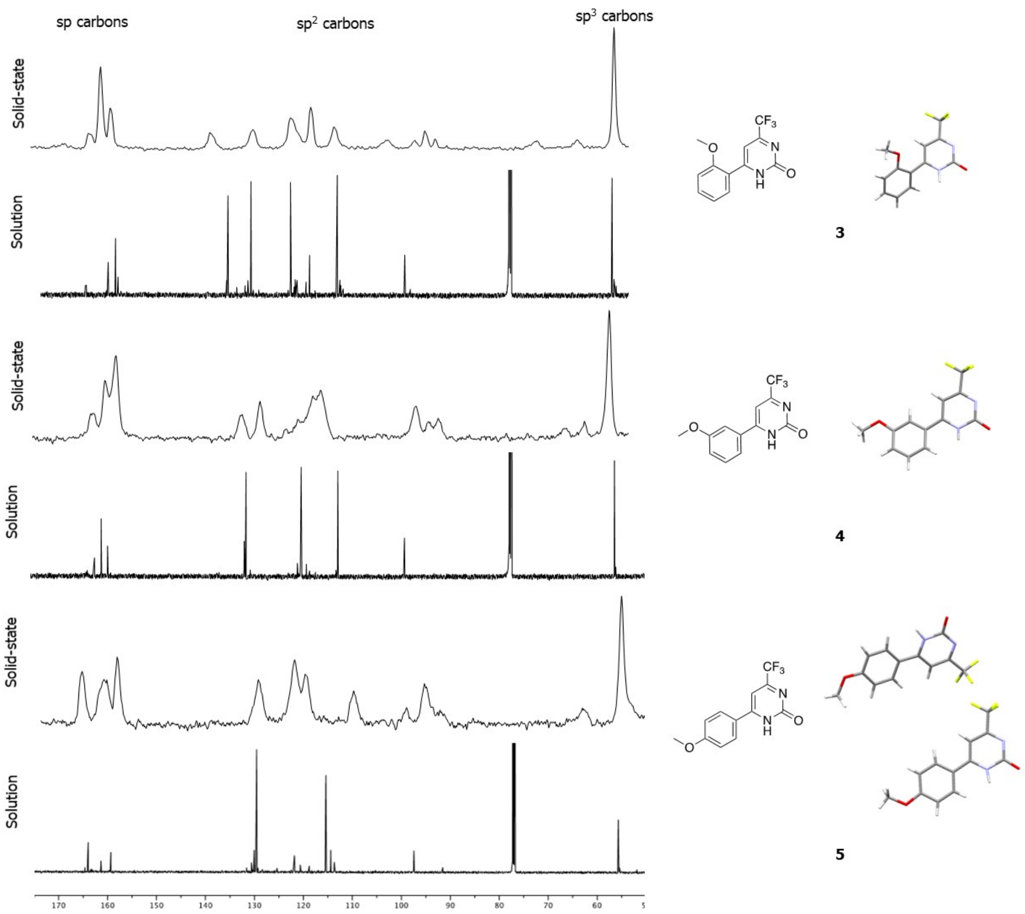

Figure S72. ^{13}C NMR spectra ($CDCl_3$, 298 K, 150 MHz) and ^{13}C CP/MAS SSNMR (298 K, 150 MHz) for compounds **3**, **4** and **5**. Symmetry-independent molecules from SC-XRD are also represented.

10. Thermal Displacement Ellipsoids

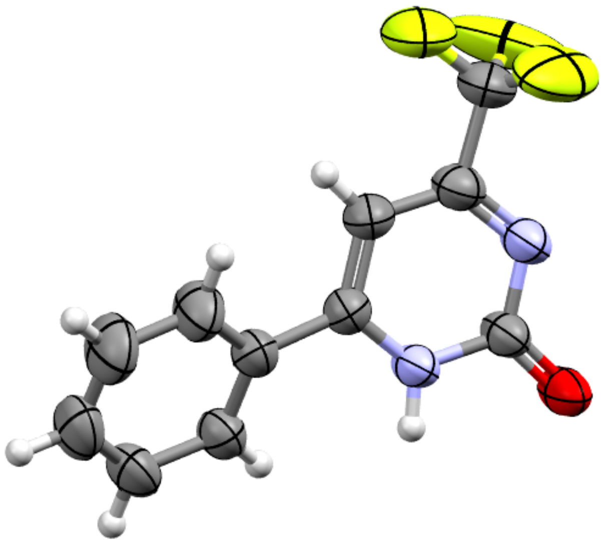

Figure S73. Thermal displacement ellipsoids of structure **1** at the 50% probability level. The hydrogen atoms are represented by spheres with arbitrary radii.

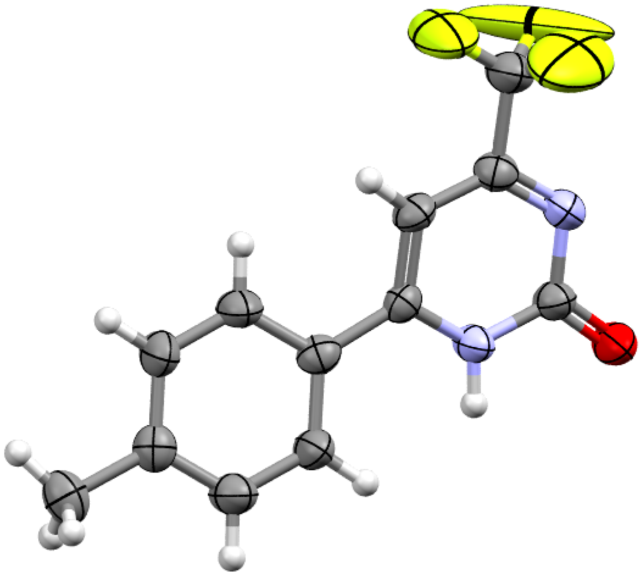

Figure S74. Thermal displacement ellipsoids of structure **2** at the 50% probability level. The hydrogen atoms are represented by spheres with arbitrary radii.

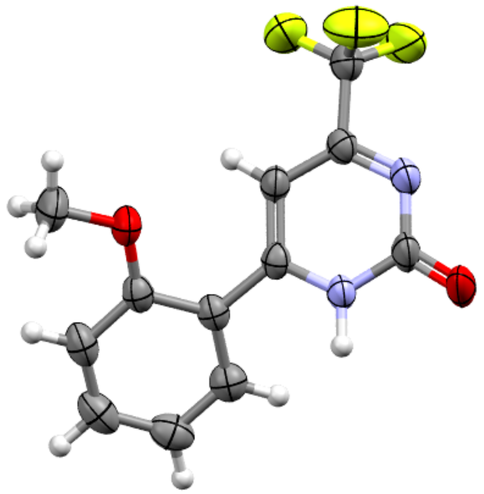

Figure S75. Thermal displacement ellipsoids of structure **3** at the 50% probability level. The hydrogen atoms are represented by spheres with arbitrary radii.

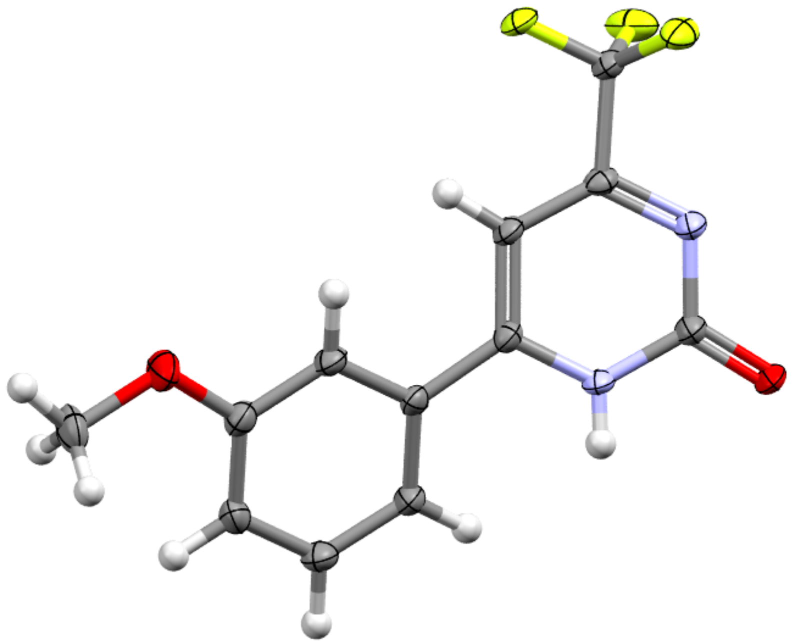

Figure S76. Thermal displacement ellipsoids of structure **4** at the 50% probability level. The hydrogen atoms are represented by spheres with arbitrary radii.

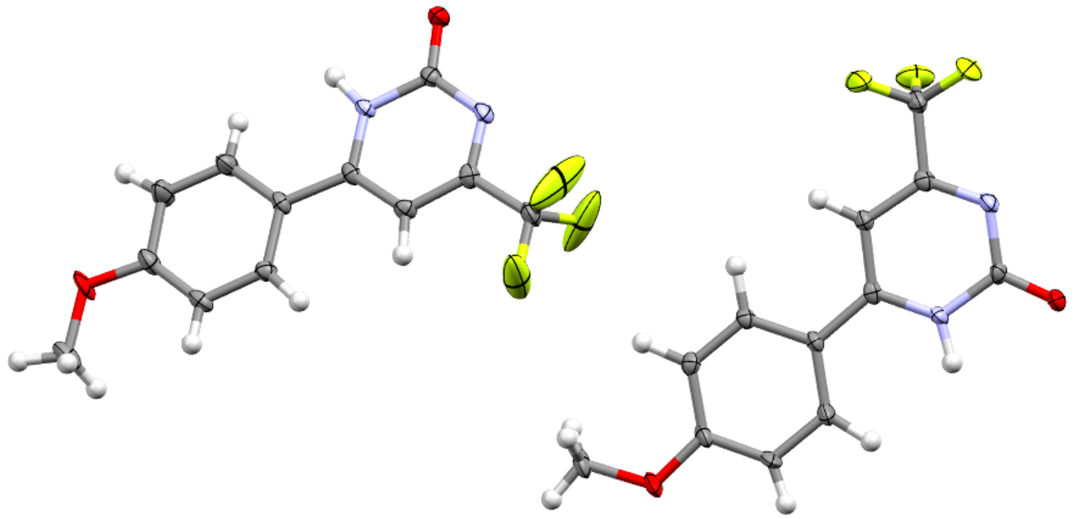

Figure S77. Thermal displacement ellipsoids of structure **5** at the 50% probability level. The hydrogen atoms are represented by spheres with arbitrary radii.

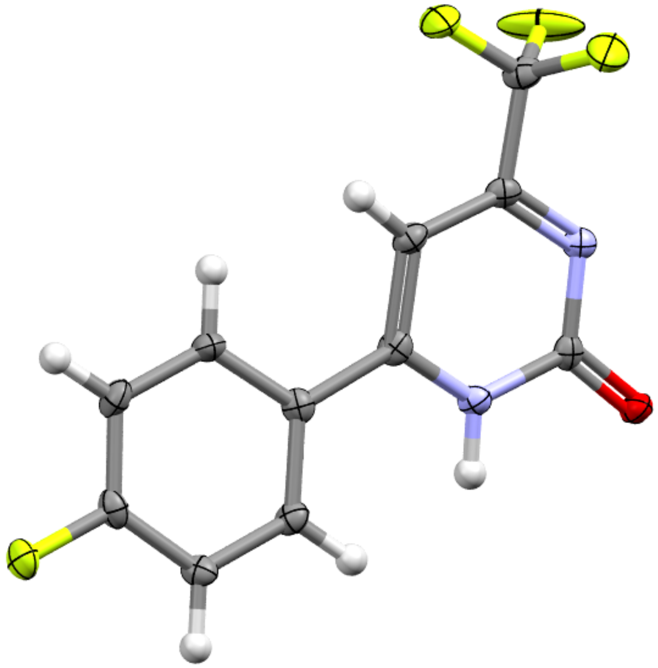

Figure S78. Thermal displacement ellipsoids of structure **6** at the 50% probability level. The hydrogen atoms are represented by spheres with arbitrary radii.

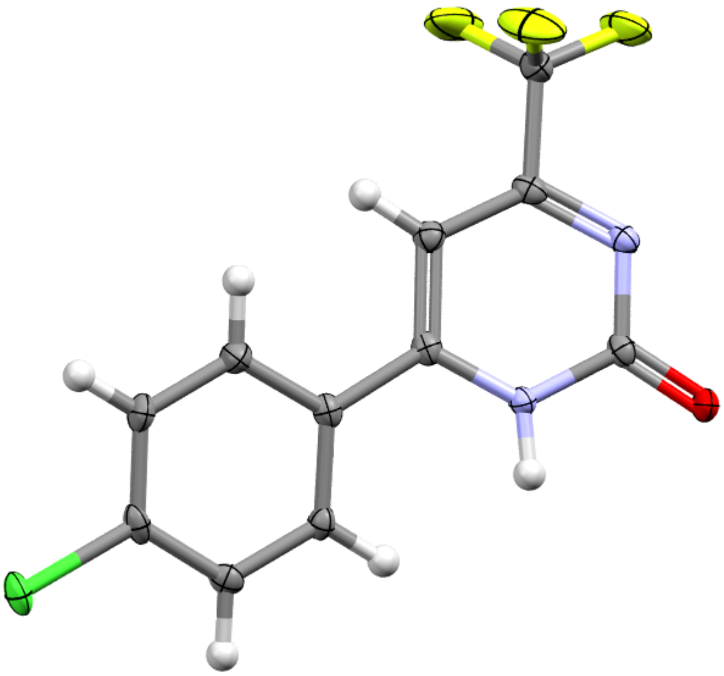

Figure S79. Thermal displacement ellipsoids of structure **7** at the 50% probability level. The hydrogen atoms are represented by spheres with arbitrary radii.

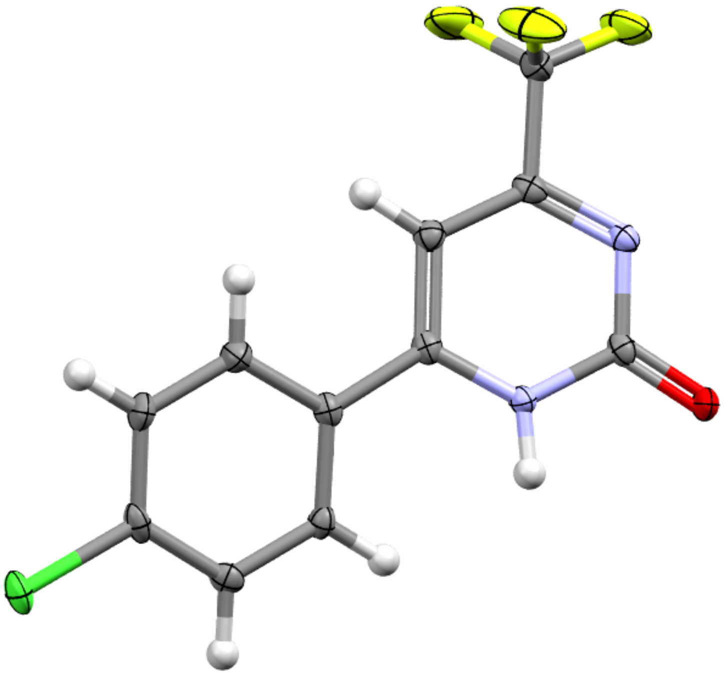

Figure S80. Thermal displacement ellipsoids of structure **8** at the 50% probability level. The hydrogen atoms are represented by spheres with arbitrary radii.

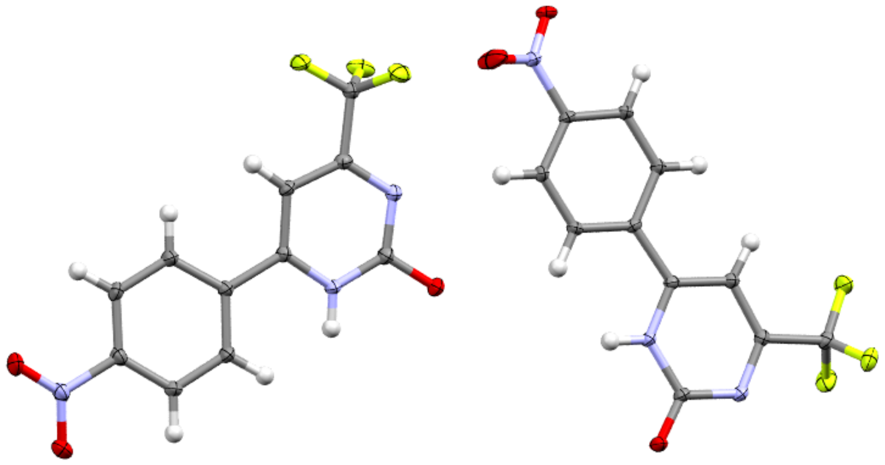

Figure S81. Thermal displacement ellipsoids of structure **9** at the 50% probability level. The hydrogen atoms are represented by spheres with arbitrary radii.

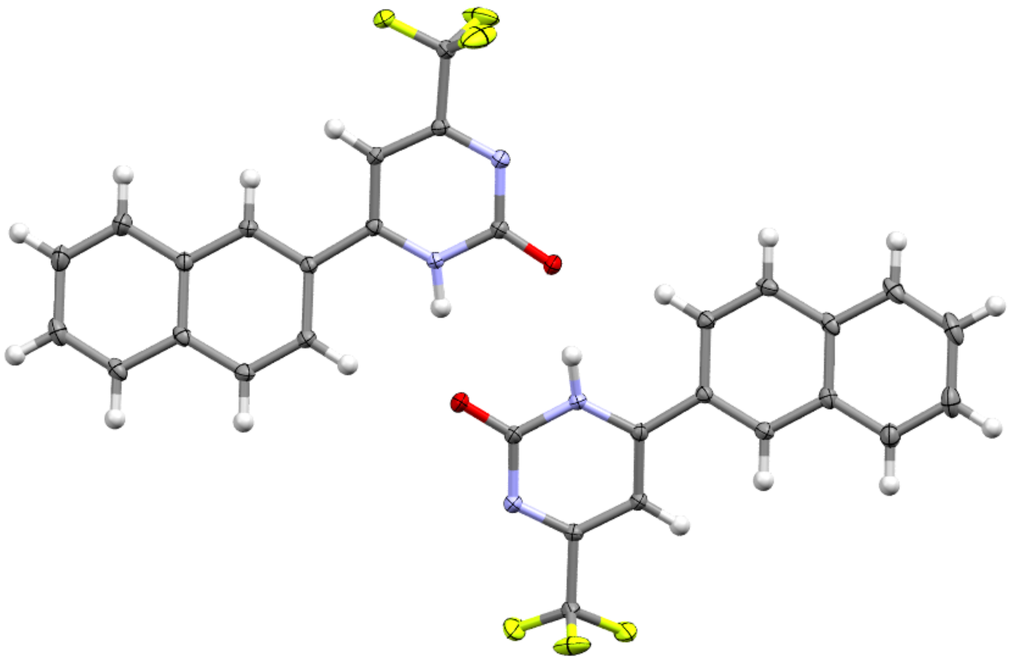

Figure S82. Thermal displacement ellipsoids of structure **10** at the 50% probability level. The hydrogen atoms are represented by spheres with arbitrary radii.

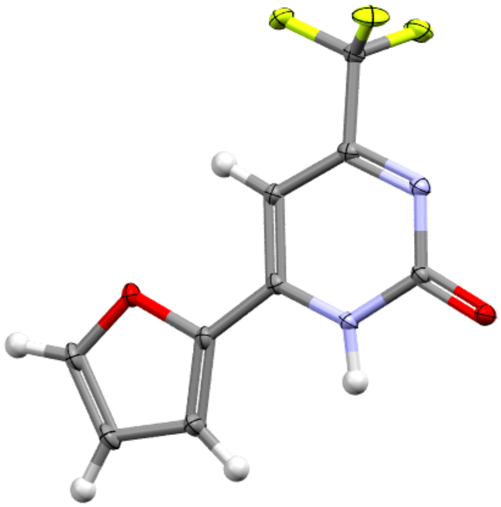

Figure S83. Thermal displacement ellipsoids of structure **11** at the 50% probability level. The hydrogen atoms are represented by spheres with arbitrary radii.

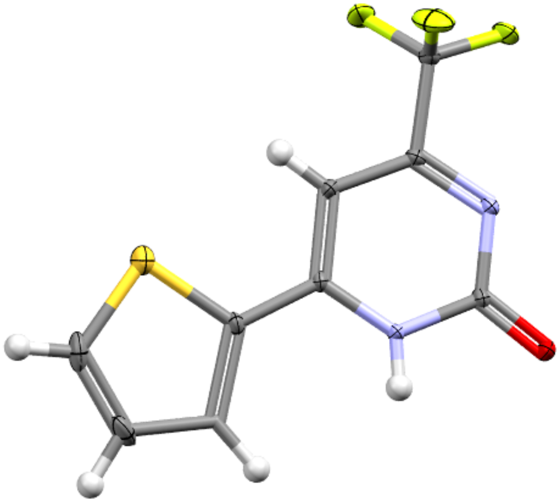

Figure S84. Thermal displacement ellipsoids of structure **12** at the 50% probability level. The hydrogen atoms are represented by spheres with arbitrary radii.

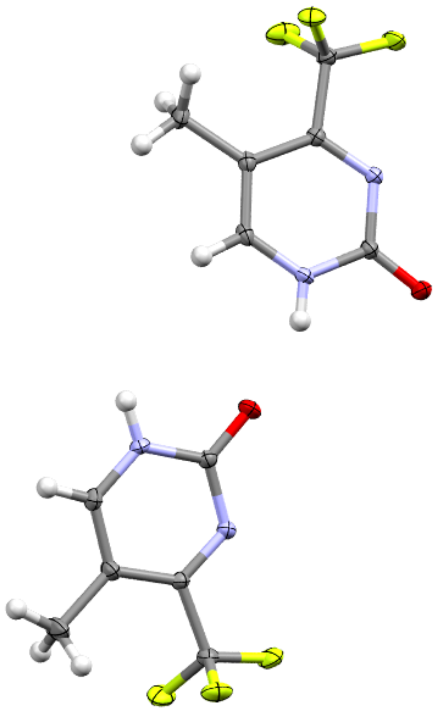

Figure S85. Thermal displacement ellipsoids of structure **13** at the 50% probability level. The hydrogen atoms are represented by spheres with arbitrary radii.

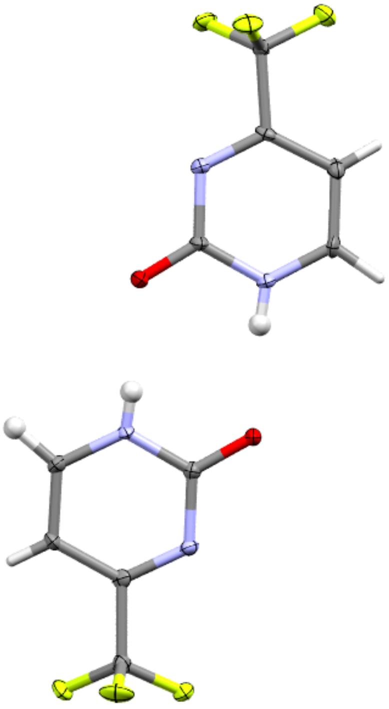

Figure S86. Thermal displacement ellipsoids of structure **14** at the 50% probability level. The hydrogen atoms are represented by spheres with arbitrary radii.

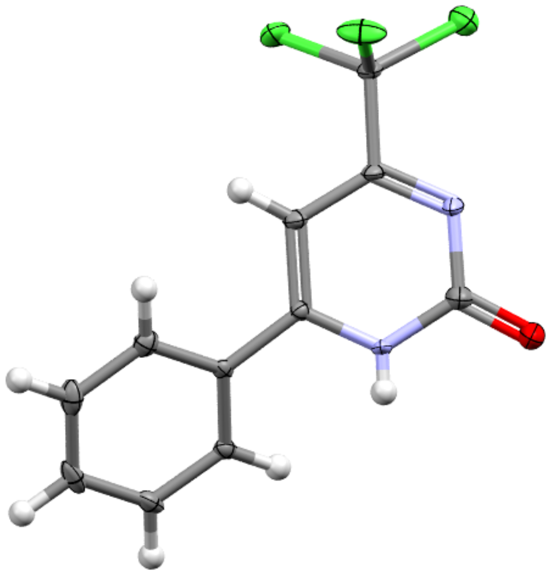

Figure S87. Thermal displacement ellipsoids of structure **15** at the 50% probability level. The hydrogen atoms are represented by spheres with arbitrary radii.

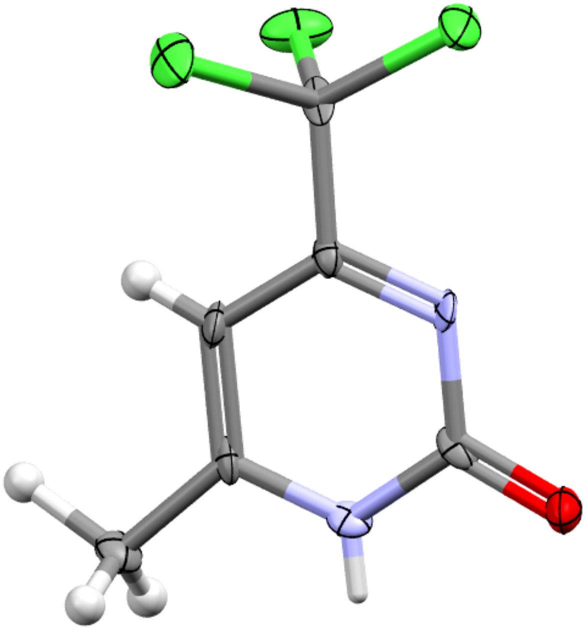

Figure S88. Thermal displacement ellipsoids of structure **16** at the 50% probability level. The hydrogen atoms are represented by spheres with arbitrary radii.

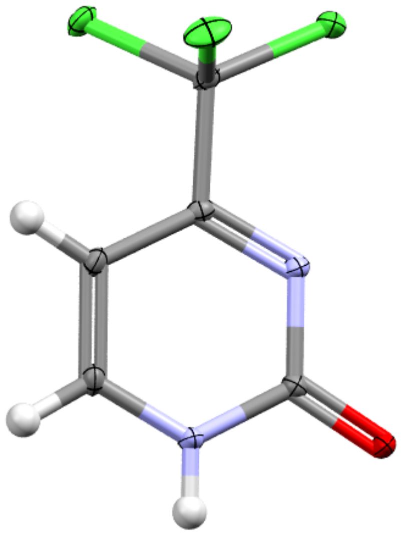

Figure S89. Thermal displacement ellipsoids of structure **17** at the 50% probability level. The hydrogen atoms are represented by spheres with arbitrary radii.

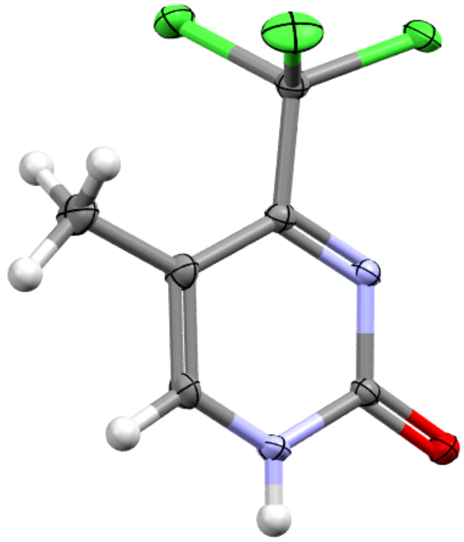

Figure S90. Thermal displacement ellipsoids of structure **18** at the 50% probability level. The hydrogen atoms are represented by spheres with arbitrary radii.
